# Error semitransparent universal control of a bosonic logical qubit

**DOI:** 10.1038/s41467-026-78317-y

**Published:** 2026-10-08

**Authors:** Saswata Roy, Owen C. Wetherbee, Valla Fatemi

**Affiliations:** 1 Department of Physics, Cornell University, Ithaca, NY USA; 2 Department of Physics, University of California, Berkeley, CA USA; 3 School of Applied and Engineering Physics, Cornell University, Ithaca, NY USA

**Keywords:** Quantum information, Qubits

## Abstract

Bosonic codes offer hardware-efficient quantum error correction, recently achieving beyond-breakeven quantum memory. However, fault-tolerant logical operations remain a critical bottleneck. While error transparent (ET) gates offer a pathway to resolve this, experimental demonstrations remain limited to phase gates. Here, we introduce a dynamic encoding subspaces framework that enables simple linear drives to accomplish universal logical gates that are error semi-transparent (EsT) to oscillator photon loss. Our EsT logical gate set of {*X*, *H*, *T*} exhibited a five-fold reduction in infidelity conditioned on photon loss, extended active-manipulation lifetimes with quantum error correction, and enabled a composite EsT non-Clifford operation using an eight-gate sequence. Our approach is compatible with methods for detectable ancilla errors and is readily extensible to other rotation-symmetric codes, offering an approach to error-mitigated universal control of bosonic logical qubits with the standard quantum control toolkit and a coherent framework for incorporating more sophisticated quantum control methods.

## Abstract (web summary)

Error transparency is an alternative approach to quantum fault-tolerance relying on multi-body Hamiltonians whose evolution is unaffected - to first order - by noise. Here, the authors show a dynamic encoding subspace framework achieving universal control of Bosonic logical qubits with error semitransparent gates, correcting most single-photon loss errors in the Bosonic mode during logical gate operations.

## Introduction

Realization of quantum computation faces the challenge of noise destroying encoded quantum information. Quantum error correction (QEC) can tackle this problem by encoding logical quantum information with redundancy to correct physical errors. Recent experiments demonstrated implementation of QEC to protect stored logical quantum information in large arrays of physical qubits^1–4^ and in single harmonic oscillators^5–11^. The latter approach, in which bosonic codes take advantage of the large Hilbert space of a harmonic oscillator, has emerged as a leading platform for hardware-efficient quantum information processing^12–14^ with multiple experiments crossing the break-even point^5–10^.

Realized experiments focus on logical qubits that are protected from errors while the qubit is idling. Fault-tolerant quantum computing also requires fault-tolerant processing of quantum information. In arrays of physical qubits, this is accomplished via a set of transversal gates and magic states^15^. For bosonic codes, the Hilbert space is not as easily separable, so the more general formalism of error-transparent (ET) gates^16,17^ or path-independent gates^18,19^ provides a path to fault-tolerant manipulation. The fault tolerance can be towards the errors of the oscillator and/or the ancilla. In particular, ET gates are designed to have similar trajectories in code and error subspaces within the large Hilbert space. Experiments demonstrated ET^20^ and path-independent^21^ phase gates in binomial logical qubits. However, universal quantum computation requires implementation of amplitude mixing gates such as the X or H gates. While conventional (non-ET) universal control was accomplished in Ref. ^11^, realization of ET universal control remains an outstanding challenge. Overcoming this bottleneck to achieve ET universal control would enable fault-tolerant universal computation and higher circuit depths necessary for quantum signal processing(QSP) and advanced algorithms^22^.

Theoretically, the implementation of amplitude mixing gates using non-linear drives has been proposed^20,23^. These operations achieve error transparency by matching the phase and trajectory of the evolution of the code and error subspaces while staying entirely within the static code and error subspaces. However, this approach presents significant experimental challenges due to the requirement of particularly strong nonlinear drives and extremely strict confinement to the static logical spaces, which are not currently natural or high-fidelity approaches for oscillator control^24,25^.

Here, we propose considering dynamic code and error subspaces as a framework to relax the static codespace requirements for error transparency. This allows us to achieve error semi-transparent (EsT) operations using only linear drives on both the oscillator and ancilla, which is the standard optimal control toolkit for circuit quantum electrodynamics (cQED)^26–28^. These simpler controls also ensure fast gates, high reliability, and fewer spurious parametric processes that can complicate the dynamics. The drawback is that, formally, exact error transparency for oscillator photon loss, the primary source of loss for bosonic codes, cannot be achieved because linear drive Hamiltonians cannot strictly commute with the photon loss errors on the codespace. Nonetheless, this tradeoff can be explored for obtaining error correction gain for actively controlled logical quantum information.

Using pulses numerically optimized for dynamic subspace error transparency to bosonic photon loss, we experimentally demonstrate universal control of a binomial logical qubit with *X*, *H*, and *T* gates. We compare the performance with that of ordinary, non-ET gates (henceforth named Ord gates), finding that EsT gates enhance performance in the presence of photon loss errors occurring during gate operations. We observe an average factor of five reduction in logical infidelity for the actively controlled logical states conditioned on a photon loss event during the gate. Commensurately, the EsT gates perform significantly better than the Ord gate after applying error correction following the operation. Further, we show that distinct gates from the set are compatible for digital construction of arbitrary unitary operations^29,30^ while preserving the EsT property.

## Results

### Theoretical concept

We begin by defining ideal ET operations. As described in Refs. ^16,17,20^, a logical unitary *U* is ET if it commutes with the error set {*E*_*j*_} throughout the operation, such that $U(T,t){E}_{j}U(t,0)\left.| {\psi }_{L}(0)\right\rangle={e}^{i{\phi }_{j}(t)}{E}_{j}U(T,0)\left.| {\psi }_{L}(0)\right\rangle$, $\forall j,t,\left.| {\psi }_{L}(0)\right\rangle$, where *U*(*t*_2_, *t*_1_) describes the unitary operation from time *t*_1_ to *t*_2_, *T* is the final time such that *U* = *U*(*T*, 0), *ϕ*_*j*_(*t*) is a global phase, and $\left.| {\psi }_{L}(0)\right\rangle$ denotes a state in the code space at time *t* = 0. If the code and error spaces are static, meaning *U*(*t*, 0) remains a logical unitary for all *t*, this ET condition can be satisfied by engineering the Hamiltonian to act identically within the code and error spaces: ${P}_{j}^{{{\dagger}} }H(t){P}_{j}={P}_{C}^{{{\dagger}} }H(t){P}_{C}+{d}_{j}(t){P}_{C},\forall j,t$, where, *P*_*j*_ ∝ *E*_*j*_*P*_*C*_ is the projector from the code space to the error space corresponding to the error *E*_*j*_, *P*_*C*_ is the codespace projector and *d*_*j*_(*t*) is a complex number.

To relax the requirement of static code and error spaces, we propose defining an instantaneous codespace

1 $${{{\mathcal{C}}}}(t)={{{\rm{span}}}}\{U(t,0)\left.| {\psi }_{L}(0)\right\rangle | \left.| {\psi }_{L}(0)\right\rangle \in {{{\mathcal{C}}}}(0)\}\,,$$

with instantaneous projectors *P*_*C*_(*t*) = *U*(*t*, 0)*P*_*C*_*U* ^†^(*t*, 0). We can define a corresponding instantaneous error space (${{{{\mathcal{E}}}}}_{j}(t)$) as

2 $${{{{\mathcal{E}}}}}_{j}(t)={{{\rm{span}}}}\{{E}_{j}\left.| {\psi }_{L}(t)\right\rangle | \left.| {\psi }_{L}(t)\right\rangle \in {{{\mathcal{C}}}}(t)\}\,,$$

with projector ${P}_{{{{{\mathcal{E}}}}}_{j}}(t)\propto {E}_{j}{P}_{C}(t){E}_{j}^{{{\dagger}} }$. Then we need to enforce three conditions on our engineered dynamics for error transparency.

First, the instantaneous code space must remain valid and correctable at all times, satisfying the QEC conditions (Knill-Laflamme conditions) throughout the trajectory. For a general error set {*E*_*k*_}, this requires that the projection of all error products into the instantaneous code space is proportional to the logical identity:

3 $${P}_{C}(t){E}_{i}^{{{\dagger}} }{E}_{j}{P}_{C}(t)={c}_{ij}(t){P}_{C}(t)\,,\quad \forall i,j,$$

where *c*_*ij*_(*t*) are complex coefficients.

Second, the evolved error space under the application of the unitary should coincide with the instantaneous error space defined above. This can be written as

4 $$U(t,0){{{{\mathcal{E}}}}}_{j}(0)={{{{\mathcal{E}}}}}_{j}(t)\,,\quad \forall j,t,$$

Third, the Hamiltonian must drive identical dynamics within these instantaneous code and error subspaces:

5 $${P}_{j}^{{{\dagger}} }(t)H(t){P}_{j}(t)={P}_{C}^{{{\dagger}} }(t)H(t){P}_{C}(t)+{d}_{j}(t){P}_{C}(t)\,,\forall j,t,$$

where *d*_*j*_(*t*) is a time-dependent complex number. Fulfilling these three conditions in the dynamic subspaces, the ET property can in principle be maintained throughout the operation.

To quantify the deviation from ideal ET in our dynamic subspaces, we introduce three time-dependent metrics: QEC violation, instantaneous leakage, and trajectory mismatch. Here we will define them analytically, and later we will assess the measures for the gates derived for our particular setup.

To evaluate deviations from ideal instantaneous correctability, we define the QEC violation metric, Δ_QEC_(*t*) ^31^ (see Supplementary Section I for details). Assuming a two-dimensional logical subspace, we define the 2 × 2 projected error matrices as ${M}_{ik}(t)={P}_{C}(t){E}_{i}^{{{\dagger}} }{E}_{k}{P}_{C}(t)$. From this matrix *M*_*ik*_(*t*), the QEC violation metric isolates and sums the magnitude of the uncorrectable Pauli components (logical bit, phase, and joint bit-phase errors) generated across all pairs of errors {*E*_*i*_, *E*_*k*_}:

6 $${\Delta }_{{{{\rm{QEC}}}}}(t)={\sum}_{i,k}{\left\Vert {M}_{ik}(t)-\dfrac{1}{2}{{\mbox{Tr}}}\left[{M}_{ik}(t)\right]{P}_{C}(t)\right\Vert }_{2}\,,$$

where ∥ ⋅ ∥_2_ denotes the 2-norm when applied to matrices, and the standard Euclidean norm when applied to vectors.

The instantaneous leakage, ${L}_{{{{{\mathcal{E}}}}}_{j}}(t)$, measures the weight in states outside the target error manifold associated with a specific error *E*_*j*_. It is defined as the probability that the state, having undergone an error *E*_*j*_, drifts outside the instantaneous error subspace ${{{{\mathcal{E}}}}}_{j}(t)$:

7 $${L}_{{{{{\mathcal{E}}}}}_{j}}(t)=1-\,{{\mbox{Tr}}}\,\left[{P}_{{{{{\mathcal{E}}}}}_{j}}(t){\rho }_{j}(t)\right]\,,$$

where *ρ*_*j*_(*t*) is the density matrix at time *t*, when initialized as the maximally mixed state in the error space ${{{{\mathcal{E}}}}}_{j}(0)$ and evolved unitarily under the driven Hamiltonian without losses.

To assess the preservation of coherence within the subspace, we define the state-dependent trajectory mismatch, ${\eta }_{{{{{\mathcal{E}}}}}_{j},\psi }(t)$. This metric quantifies the unitary rotation error by calculating the Euclidean distance between the Bloch vectors of the code state and the projected error state in the instantaneous subspaces:

8 $${\eta }_{{{{{\mathcal{E}}}}}_{j},\psi }(t)={\left\Vert {{{{\bf{r}}}}}_{C}(t)-{{{{\bf{r}}}}}_{{E}_{j}}(t)\right\Vert }_{2}\,,$$

where the Bloch vectors are given by ${{{{\bf{r}}}}}_{C}(t)=\,{\mbox{Tr}}\,[{{{{\mathbf{\sigma }}}}}_{C}(t){\rho }_{\left.| \psi \right\rangle }(t)]$ and ${{{{\bf{r}}}}}_{{E}_{j}}(t)=\,{\mbox{Tr}}\,[{{{{\mathbf{\sigma }}}}}_{{E}_{j}}(t){\tilde{\rho }}_{| {\psi }_{{E}_{j}}\rangle }(t)]$. Here, **σ**_*C*_(*t*) and ${{{{\mathbf{\sigma }}}}}_{{E}_{j}}(t)\propto {E}_{j}{{{{\mathbf{\sigma }}}}}_{C}(t){E}_{j}^{{{\dagger}} }$ are vectors of the logical Pauli operators in the instantaneous code and error subspaces. The density matrices ${\rho }_{\left.| \psi \right\rangle }(0)$ and ${\rho }_{| {\psi }_{{E}_{j}}\rangle }(0)$ are initialized as a pure logical state $\left.| \psi \right\rangle \in {{{\mathcal{C}}}}(0)$ and the corresponding error space state $|{\psi }_{{E}_{j}}\rangle \propto {E}_{j}\left.| \psi \right\rangle$, respectively. After the unitary evolution by the system Hamiltonian and Schrodinger equation, the evolved states are projected onto the instantaneous code and error spaces, respectively, and normalized to obtain ${\tilde{\rho }}_{\left.| \psi \right\rangle }$ and ${\tilde{\rho }}_{| {\psi }_{{E}_{j}}\rangle }(t)$ (note that ${\tilde{\rho }}_{\left.| \psi \right\rangle }(t)={\rho }_{\left.| \psi \right\rangle }(t)$ by definition of instantaneous code space).

These three metrics quantify deviations from error-transparent evolution, with optimal performance achieved as each approaches zero. A vanishing QEC violation, Δ_QEC_(*t*) = 0, ensures the evolving code words remain correctable throughout the operation. A vanishing leakage, ${L}_{{{{{\mathcal{E}}}}}_{j}}(t)=0$ implies the evolving error space and instantaneous error space coincide. Finally, a vanishing trajectory mismatch, ${\eta }_{{{{{\mathcal{E}}}}}_{j},\psi }(t)=0$ for all initial states $\left.| \psi \right\rangle$, indicates that the instantaneous error subspace evolution is identical to the code subspace evolution throughout the gate operation. These three metrics being simultaneously satisfied at all times ensures the evolution satisfies the condition for dynamic error transparency.

### Application to a binomial code

**Fig. 1:**
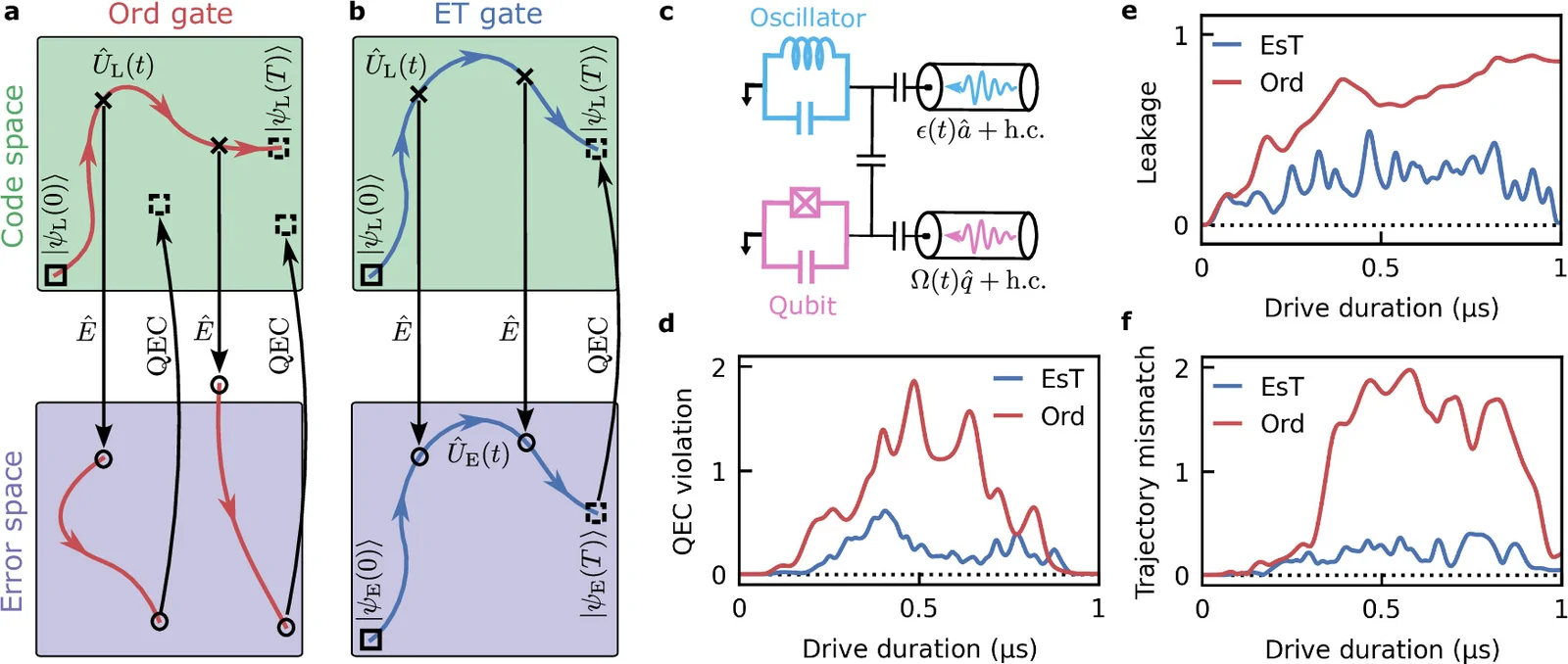
Concept and operation of the ET gate. During a logical operation *U*_*L*_(*t*) that takes the logical code space state $\left.| {\psi }_{L}(0)\right\rangle$ at time *t* = 0 to $\left.| {\psi }_{L}(T)\right\rangle$ at time *t* = *T*, an error may occur at any time *t*, taking the state from code space to an orthogonal error space. **a** For an ordinary (Ord) gate, an error at any point destroys the effect of the operation in the error space. Importantly, the final state depends on the exact time of the error occurring, which is a probabilistic event. **b** For an error-transparent (ET) operation, the trajectories in error and code space are identical. Hence, an error happening at any time takes the state through a deterministic path in error space and ends at the same final state, preserving the effect of the gate in error space. Therefore, a quantum error correction (QEC) process can be used to detect and correct the error at the end of the operation, making the ET operation tolerant of errors during it. **c** An ancilla transmon qubit and an oscillator coupled to it are driven with numerically optimized pulses for realization of ET operations on an encoded logical qubit in the oscillator. **d** QEC violation for the evolved code space basis states during a logical X operation on a binomial logical qubit encoded in the oscillator. The numerically optimized error semi-transparent gate (EsT gate, blue trace) has minimal violation compared to the Ord gate (red trace). **e** Leakage out of the instantaneous error space and (**f**) trajectory mismatch in code and error subspaces also show smaller violation for EsT gate compared to the Ord gate. The trajectory mismatch metric is calculated for an initial state $\left.| {0}_{L}\right\rangle$.

We now turn to applying this concept to bosonic codes, specifically the binomial ‘kitten’ code. To realize such EsT operations leveraging a dynamic subspace framework, we encode a logical qubit in a superconducting cavity (harmonic oscillator), dispersively coupled to a transmon qubit ancilla^32^ as shown in Fig. 1c. The system Hamiltonian at low order is given by:

9 $$H/\hbar={\omega }_{a}{\hat{a}}^{{{\dagger}} }\hat{a}+{\omega }_{q}{\hat{q}}^{{{\dagger}} }\hat{q}+\chi {\hat{a}}^{{{\dagger}} }\hat{a}{\hat{q}}^{{{\dagger}} }\hat{q}+\dfrac{{K}_{a}}{2}{\hat{a}}^{{{\dagger}} 2}{\hat{a}}^{2}+\dfrac{{K}_{q}}{2}{\hat{q}}^{{{\dagger}} 2}{\hat{q}}^{2}\,,$$

where $\hat{a}$ and $\hat{q}$ are the annihilation operators for the cavity and transmon respectively, *χ*/2*π* = -3.66MHz is the dispersive coupling of the transmon and cavity, *K*_*a*_/2*π *= -22KHz is the self-Kerr of the cavity, *K*_*q*_/2*π *= -180MHz is the self-Kerr of the qubit (Supplementary Section II describes details of experimental package). The cavity has *T*_1_= 180 *μ*s and *T*_2_=290 *μ*s whereas the ancilla transmon has *T*_1_= 70 *μ*s and *T*_2_=30 *μ*s. This is the same package as used in Roy, et al^25^.

In the cavity, we encode the binomial ‘kitten’ code^33,34^, which allows correction of single-photon loss errors in the cavity. The codespace for this binomial code is defined on the even parity subspace as ${{{\mathcal{C}}}}={{{\rm{span}}}}\{\left.| {0}_{L}\right\rangle,\left.| {1}_{L}\right\rangle \}$, where $\left.| {0}_{L}\right\rangle=\frac{\left.| 0\right\rangle+\left.| 4\right\rangle }{\sqrt{2}},\left.| {1}_{L}\right\rangle=\left.| 2\right\rangle$ are the two logical codewords. When a single-photon loss error happens in the cavity, the logical state jumps into the orthogonal odd-parity error space ${{{\mathcal{E}}}}={{{\rm{span}}}}\{\left.| {0}_{E}\right\rangle,\left.| {1}_{E}\right\rangle \}$, where $\left.| {0}_{E}\right\rangle=\left.| 3\right\rangle,\left.| {1}_{E}\right\rangle=\left.| 1\right\rangle$ are the two error words.

The flexibility of dynamic subspaces allows for the use of simpler linear drives that traverse trajectories outside this static code and error spaces to achieve fast, non-adiabatic operations while optimizing for ET (Fig. 1b) through the gate. We consider the drive Hamiltonian

10 $${H}_{{{{\rm{drive}}}}}(t)/\hslash=\epsilon (t)\hat{a}+\Omega (t)\hat{q}+\,{\mbox{h.c.}}\,$$

However, evolutions of the codespace under linear drive on the oscillator does not generically commute with photon loss operator $\hat{a}$, the primary loss channel for the binomial code ${{{\mathcal{C}}}}$, on the codespace. Consequently, exact ET is not expected to be achieved in this setup, but trading exactness for a simpler and more scalable experimental setup may offer practical advantage and higher experimental fidelities by improving reliability and reducing spurious error channels. We refer to operations that deviate from exact error transparency as error semi-transparent (EsT). We also note that, compared to previous ET and path-independent phase gate implementations^20,21^, these EsT gates can be much faster and achieve higher fidelity as there is no constraint of adiabaticity.

We construct the target EsT unitary by numerically engineering the time-dependent drive Hamiltonian (*H*_drive_(*t*)). We optimize complex drive amplitudes simultaneously applied on the cavity (*ϵ*(*t*)) and the ancilla qubit (*Ω*(*t*)) (Fig. 1c), using GRAPE^26,27,35^ for accomplishing logical *X*, *H*, and *T* gates on the binomial code. The Ord gates are optimized with the single objective of obtaining the highest-fidelity operation in the code space. For EsT gates, an appropriately weighted objective of error transparency (see Eq. (C2), (C3)) is added in addition to obtaining high-fidelity operation in the code and error subspaces. To facilitate differentiability, we utilize a cost function based on state overlaps and fidelities rather than commutator norms and projectors for defining error transparency. Further details of the numerical optimization are described in Supplementary Section III. Logical *X* and *H* gates are implemented with a duration of 1 *μ*s with simultaneous qubit and cavity drives, while the *T* gate is realized with only qubit drives in 0.6 *μ*s. To appropriately compare the performance of EsT and Ord gates, we select pulses that reach the same maximum active Fock state level, defined as the highest Fock state $\left.| n\right\rangle$ to achieve a transient probability of occupation greater than 0.01.

We simulate the dynamics of the oscillator under the Ord and EsT gates and calculate the violation metrics defined in Eqs. (6)–(8). We observe that optimization for the EsT gate successfully minimizes violation of the QEC conditions in the instantaneous code space, reducing leakage out of the instantaneous error space due to photon loss, and enforcing trajectory and coherence matching in the instantaneous code and error spaces. The Ord gate has no such constraint and therefore exhibits substantial QEC violation, leakage, and trajectory mismatch due to photon loss. We compare the QEC violation, leakage of the evolved error space out of the instantaneous error space during the operation, and the trajectory mismatch in Fig. 1d–f respectively. Trajectory mismatch is plotted for the evolution of the initial state $\left.| {0}_{L}\right\rangle$. Values closer to zero suggest a lower violation of the instantaneous conditions required to satisfy ET. These plots confirm how the optimizer enables the pulse to be EsT.

While these metrics quantify ET at a specific moment, the final gate fidelity will depend on both the immediate impact of the error and the subsequent evolution under the remaining control pulse. To capture this full effect, we can evaluate the gate fidelity conditioned on a single photon loss event occurring at an arbitrary time during the operation. As detailed in Supplementary Section IV, the EsT gate maintains a higher conditional fidelity across the pulse duration compared to the Ord gate.

### Experimental results

**Fig. 2:**
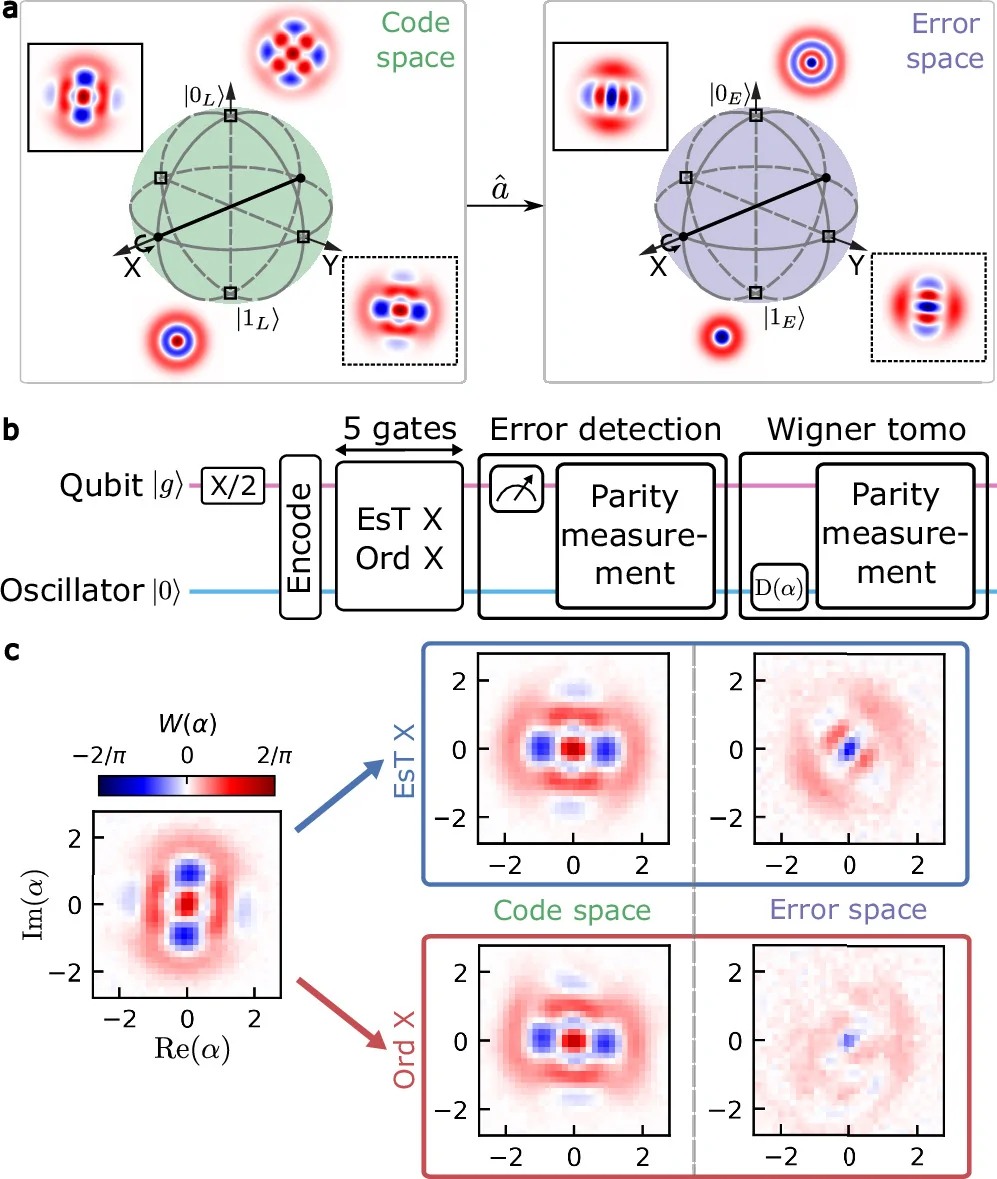
Wigner visualization of error transparency. **a** Logical Bloch sphere of code space (left) and error space (right) corresponding to single photon loss errors in the oscillator for binomial 'kitten' code. The cardinal points and their theoretical Wigner functions are marked on the Bloch sphere. Starting from the state $\left\vert -{Y}_{L}\right\rangle=(\left.| {0}_{L}\right\rangle -i\left.| {1}_{L}\right\rangle )/\sqrt{2}$ (Wigner function in solid square box), a logical X operation is performed along the X axis (marked black) to end in the state $\left.|+{Y}_{L}\right\rangle=(\left.| {0}_{L}\right\rangle+i\left.| {1}_{L}\right\rangle )/\sqrt{2}$ (Wigner function in dashed square box). **b** Experimental pulse sequence for characterizing EsT gates. After the gate operation, we read out the qubit state and measure the parity of the oscillator state to detect errors and perform Wigner tomography. Based on this parity measurement, we post-process the Wigner functions of states in code and error space. **c** Experimentally measured Wigner functions of binomial code cardinal point $\left\vert -{Y}_{L}\right\rangle=(\left.| {0}_{L}\right\rangle -i\left.| {1}_{L}\right\rangle )/\sqrt{2}$ and the state after applying five EsT or Ord X gates are shown. The left-side plots of the code-space Wigner function are conditioned on detection of no error (~90% of shots) and show similar performance for the EsT and Ord gates. The right-side plot of the error-space Wigner function is conditioned on detection of single-photon loss error (~10%) and shows EsT gate significantly preserves the phase coherence and state amplitudes of the target state compared to Ord gate. Error space Wigners show slightly different frame rotation than codespace because of qubit being in excited state for longer during the measurement.

To verify the ET property in experiments, we performed Wigner tomography on the states following the application of 5 *X* gates on the prepared state $\left\vert -{Y}_{L}\right\rangle=(\left.| {0}_{L}\right\rangle -i\left.| {1}_{L}\right\rangle )/\sqrt{2}$. Fig. 2a shows ideal Wigner functions of the initial (solid box) and final state (dashed box) after application of *X* gate in the code and error space logical Bloch spheres. We show the pulse sequence for the experiment in Fig. 2b. After applying the gate, we read out the qubit state, followed by parity measurement of the cavity state, and finally perform Wigner tomography^36^. If there is a photon loss error during the operation, the state ends in the error subspace. Based on the parity measurement before the Wigner tomography, we post-select the final state in code and error subspaces. We show the measured Wigner functions for prepared $\left\vert -{Y}_{L}\right\rangle$ states and the states in code (left) and error (right) space after EsT and ord gates in Fig. 2c. Code and error-space Wigners have different fixed global frame rotations due to the qubit being in the excited state for longer during the parity measurement process, which can be fixed with a frame rotation in software.

The Wigner functions measured in the codespace indicate that the *X* gate is accomplished and the logical states exhibit similar coherence within this subspace for both the EsT and Ord gates. Crucially, phase coherence and state amplitudes in the error space are preserved for the EsT gate, whereas they are significantly corrupted for the Ord gate.

**Fig. 3:**
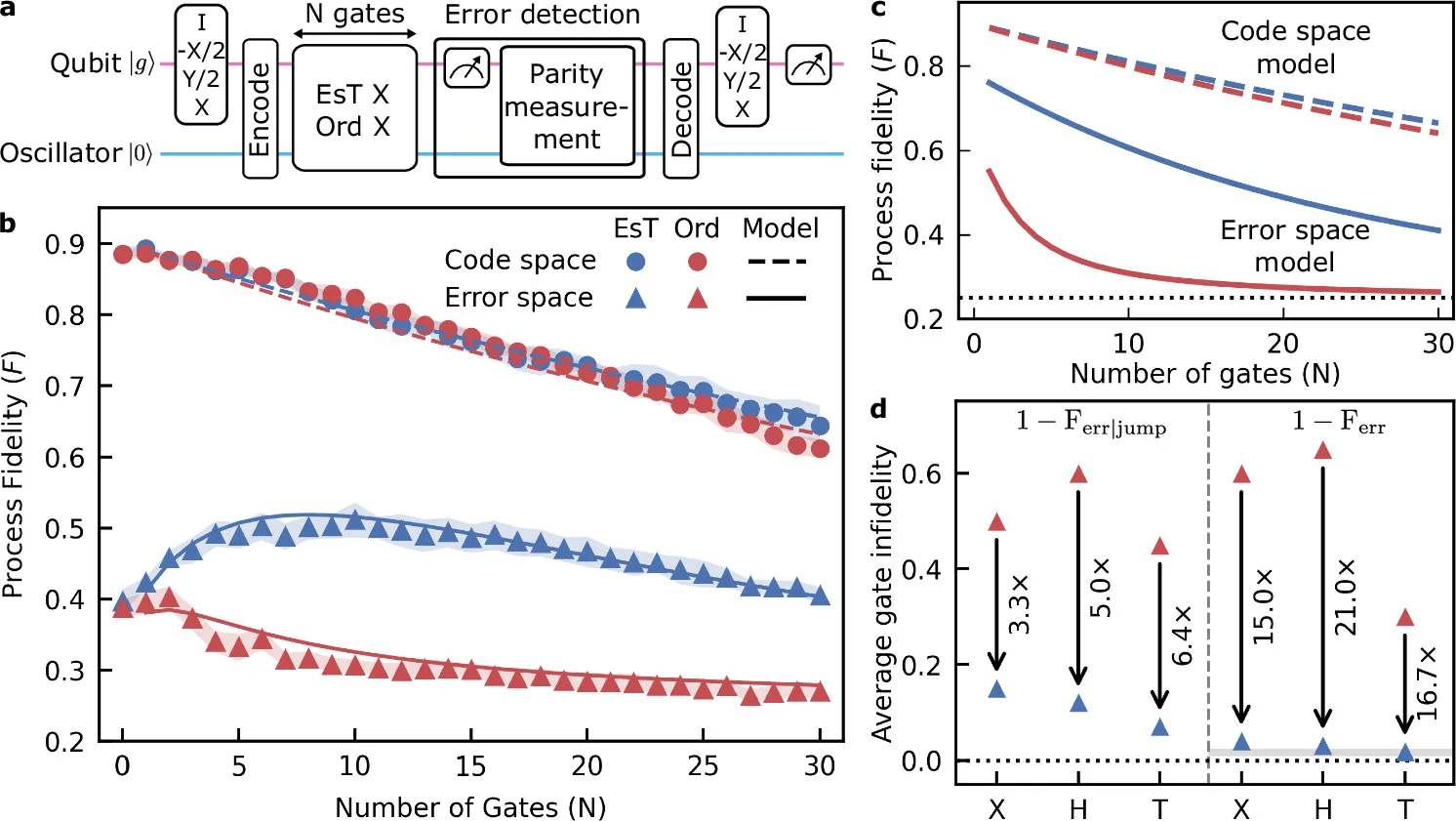
Code and error space process tomography. **a** Experimental pulse sequence to perform code and error space process tomography. Following the gate operations, we perform qubit readout and oscillator parity measurement for error detection, based on which we post-select our states into code and error space. Separate decode operations are used that respectively map the code and error space states to the ancilla qubit state for performing process tomography. **b** Experimentally measured code (dots) and error space (triangles) process fidelities for EsT and Ord X gates with number of applied gates are shown (see Supplementary Section III for details). The shaded region around the data points signifies 1 standard deviation of measured fidelities in repeated experiments. Dashed lines for code space and solid lines for error space show modeled gate performance with the number of gates. In codespace, both gates perform similarly, while in the error space, the EsT gate performs significantly better than the Ord gate. The horizontal dashed line at process fidelity of 0.25 indicates a completely incoherent process. **c** We plot the modeled code and error space process fidelities without non-idealities of the error detection process. This shows a clear difference in error space process fidelity of EsT and Ord gates. **d** We summarize the infidelities for X, H, and T gates extracted from our model for (left side of plot) error during the operation that takes a state from code space to error space (F_err∣jump_) and (right side of plot) operations in the error space (F_err_). The factor of reduction in infidelity is marked by the arrow for all the cases. The gray shading indicates the average infidelity of the gates when starting in code space.

We quantify the performance in the code and error space conditioned on single-photon loss by performing quantum process tomography (QPT)^30,37^ of the states following the sequence shown in Fig. 3a and post-selecting on the code and error space states based on the parity measurement. Different decode operations are used that respectively map code and error space states to ancilla qubit states for extracting the fidelities in those subspaces. To focus only on the oscillator photon loss error to which the gate construction is EsT, we also post-select only the cases where the qubit is in the ground state at the end of the gate operations. Details of this analysis and further experimental data are given in Supplementary Section V.

Experimentally measured fidelities for the code (circles) and error (triangles) space are shown for EsT and Ord *X* gates in Fig. 3b. To understand the dynamics and extract gate fidelities, we model the fidelity performance of the EsT and Ord gates as a function of the number of gates applied. The model assumes that a single photon loss event is equally likely to occur during any gate, ignores the small probability of two-photon loss errors, and accounts for state preparation and measurement errors by using a 91% experimentally observed encode-decode fidelity (see Supplementary Section VI for details). The modeled curves, shown as dashed lines for code space and solid lines for error space, match our experimental data well. This plot confirms that the fidelity in code space is comparable for EsT and Ord gates, whereas in the error space, EsT gates exhibit substantially higher fidelity compared to Ord gates.

The decay of error-space fidelity with gate depth appears non-intuitive at first glance. This is due to two processes that dominate over photon loss during the gates at short times (after only a few gates). The first is due to state preparation, which has an error that produces some weight in error subspaces. The second is that the parity measurement process itself has an error of ~5%. So, at short times, a state selected in the error subspace has a small probability of being there as a result of photon loss during the gate. We model these error channels to fit the observed decay curves, allowing us to estimate the expected decay profiles free of parity selection errors. (see Supplementary Section VI for details). These ‘intrinsic’ fidelities of the gates are presented in Fig. 3c. This plot further validates the similarity in code space performance and the improvement in error space performance for EsT gates compared to Ord gates.

From the model that fits experimental data, we can extract the per-gate fidelity in case of a photon loss during the operation (*F*_err∣jump_) and the fidelity of the operation in the error space (*F*_err_). For the *X* gate, *F*_err∣jump_ increases from 0.45 to 0.85, and *F*_err_ increases from 0.4 to 0.95 for the EsT gate compared to the Ord gate. We summarize these numbers in Fig. 3d for *X*, *H*, and *T* gates. Averaged across the set of gates, we observe 5 times reduction in infidelity for the case of photon loss during the operation for the EsT gate compared to the Ord gate. This improvement is achieved because of the error transparency optimization. The per-gate infidelity in error space is reduced 17 times for the EsT gate. This improvement is because the Ord gate optimization does not optimize for the fidelity of operation in error space, whereas the EsT gate is optimized for that as well. The gray shading indicates the average infidelity of the gates when starting in code space. This also raises the question of whether optimizing for operation fidelity in both code and error subspaces is sufficient, without explicit error transparency optimization. To investigate this, we introduce the LE gate, where ‘LE’ stands for “Logical and Error space" gate. The LE gate maximizes operation fidelity in the code and error subspaces separately, but does not optimize for error transparency (see Supplementary Section III for details of pulse construction). This gate shows intermediate performance that surpasses the Ord gate but remains inferior to the EsT gate. Additional experimental characterization and simulations are provided in Supplementary Section V and Supplementary Section IV, respectively.

EsT gates also enable sequencing of gates, in particular because logical gate fidelity is nearly the same for the cavity starting in either the error space or the code space. This is shown in Fig. 3d. Therefore, an error correction operation need not be applied after each gate operation, which is attractive as the error correction operations will have their own infidelities.

**Fig. 4:**
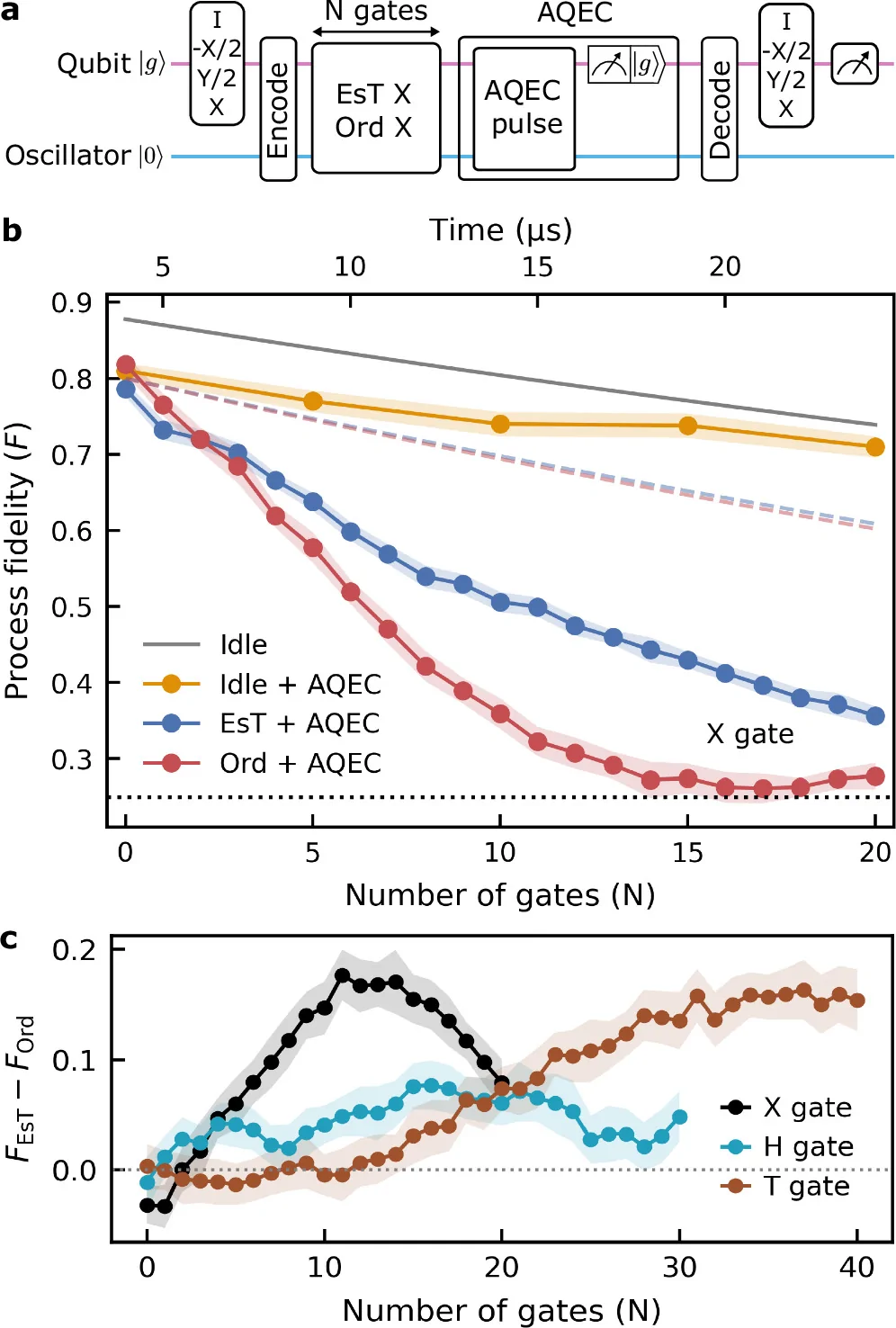
EsT gates protected by AQEC. **a** Experimental sequence to check gate performance with autonomous quantum error correction (AQEC). We perform a single AQEC step after *N* gates and perform process tomography. **b** We show the performance of EsT and Ord X gates with the number of gates applied before doing error correction. EsT gate outperforms the Ord gate, and the improvement increases with the number of gates and then decreases as uncorrectable qubit errors, two-photon loss errors in the oscillator, and control errors start to dominate. The plot also shows curves for process fidelity decay for idling and for applying an AQEC pulse after idling for an equivalent duration of N gates. The blue and red dashed lines provide a visual reference for simulated idling decay curves, calculated at an average photon number equal to the average photon number during the gate operation. The horizontal dashed line at process fidelity of 0.25 indicates a completely incoherent process, and shaded regions around the data points signify 1 standard deviation of fidelities for repeated measurements. The top X axis shows the total duration of the experiment that includes encode, decode, AQEC, and active reset of the qubit. **c** Summary of improvement in gate fidelity for the EsT gate compared to the Ord gate by applying AQEC as a function of the number of gates for X, H, and T gates.

We first take advantage of this to demonstrate that the EsT gate performance improvement in error space remains valid after recovery to code space. We investigate the performance of gates under autonomous quantum error correction (AQEC) protection by applying the experimental sequence in Fig. 4a. The AQEC pulse is numerically optimized with a duration of 1 *μ*s to recover error states to code states and transfer the error entropy to the ancilla qubit (see Supplementary Section III B). The ancilla qubit is then conditionally reset to the ground state, followed by a measurement, which takes another 1.2 *μ*s^20^. We note that other AQEC methods have also been demonstrated in recent experiments, which can potentially perform better than this AQEC technique^9,10,38,39^.

This experiment also tested the potential for concatenation, because it repeatedly applied a gate *N* times followed by a single AQEC step (see again Fig. 4a). Experimentally measured process fidelities for the EsT and Ord *X* gates are shown in Fig. 4b. As the gate depth *N* increases, the difference in process fidelity between the EsT and Ord gates widens. The improvement comes from both being EsT and better performance of the EsT gate in the error subspace. As the number of gates increases, uncorrectable two-photon loss errors in the oscillator, ancilla qubit errors, and control errors start to dominate, reducing the performance improvement. Experimentally measured process fidelities for applying a single AQEC operation after waiting for a time equivalent to the gate operations are also plotted in Fig. 4b alongside a process fidelity decay curve for idling. These curves demonstrate that while our AQEC pulse lowers the absolute measured fidelity, it actively recovers quantum information from the error space and thereby slows the decay of encoded, actively manipulated quantum information in code space. We have also added red and blue dashed lines as a visual reference for simulated idling decay curves, calculated at an average photon number equal to the average photon number during the gate operation.

We perform this experiment for *H* and *T* gates and summarize the improvement in fidelity for the EsT gates compared to the Ord gates in Fig. 4c. For *X* and *H* gates, the fidelity improvement increases with the number of gates and then decreases due to the aforementioned reasons. For the T gate, we see performance improvement up to 40 gates and expect it to decrease after that. We note here that the EsT *H* gate has a lower fidelity improvement with AQEC even though it had better error-space performance fidelities (*F*_err∣jump_ and *F*_err_) compared to the *X* gate. We attribute this to the following two facts. Due to fluctuations of device parameters, EsT gate fidelity without the AQEC was lower for the *H* gate, and the probability of oscillator photon loss during the gate is higher for the *H* gate compared to the *X* gate (see Supplementary Section V for details). This could be resolved in the future by engineering robustness objectives into the numerical optimization^40,41^. Control errors mentioned above may also be mitigated by calibration of pulses with hardware feedback using reinforcement learning methods^42,43^.

**Fig. 5:**
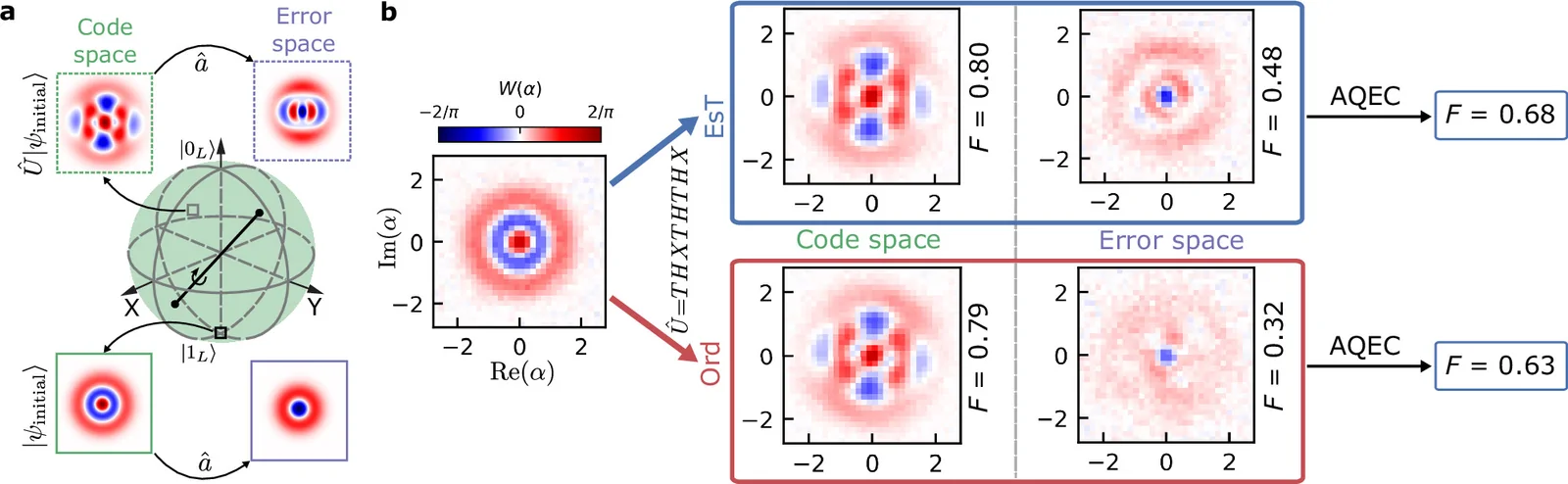
EsT protection for a non-trivial sequence. **a** Logical code space Bloch sphere showing the ideal Wigner functions before (solid box) and after (dashed) the non-trivial *THXTHTHX* operation sequence that effectively rotates the state around the marked non-trivial rotation axis (black line). The ideal error-space Wigner functions are also shown. **b** We show the experimentally measured Wigner functions of the prepared $\left.| {1}_{L}\right\rangle$ state, the final state post-selected in the code and error subspace after applying the pulse sequence comprising error EsT and Ord gates. We measure the process fidelities as reported on the side of the measured Wigner functions. Finally, we apply the AQEC pulse and measure the process fidelity. We observe a 0.05 improvement in process fidelity for the EsT gate sequence compared to the Ord gate sequence.

Finally, we demonstrate that the EsT property is preserved when gates are concatenated by performing a non-trivial unitary with the gate sequence (*THXTHTHX*) on a prepared $\left.| {1}_{L}\right\rangle$ state. This gate performs an effective clockwise rotation of 158 degrees around the rotation axis (0.47, 0.75, 0.47), as shown on the code-space Bloch sphere in Fig. 5. Ideal Wigner functions of the initial state (solid box) and final state (dashed box) in code and error space are also shown. Fig. 5b shows the experimentally measured Wigner function of the prepared $\left.| {1}_{L}\right\rangle$ state. After applying the gate sequence, following the protocol shown in Fig. 2b, we post-select our states in code and error spaces based on parity measurement and measure Wigner functions of states in those subspaces. Both gates perform the desired operation with similar fidelity in code space. In the error space, the EsT gate preserves the phase coherence and state amplitudes of the target state better than the Ord gate. Fidelities besides the Wigners are experimentally measured process fidelities in code and error subspaces. Upon applying AQEC, we recovered process fidelities of 0.68 and 0.63, respectively, for the EsT and Ord gates, representing a net fidelity improvement of 0.05 for the operation sequence.

We also simulate this sequence to understand the fidelities without SPAM and control errors. We get code space fidelities of 0.94 and 0.92 for the EsT and Ord gate sequences, respectively. In the error space conditioned on single-photon loss of the oscillator, the EsT sequence achieves a fidelity of 0.82 compared to 0.35 for the Ord sequence. This shows a 3.6-fold reduction in infidelity in the error space. Following AQEC, the final fidelities are 0.86 for EsT and 0.82 for the Ord sequence. This confirms that the optimized gates are mutually compatible, enabling synthesis of universal control over oscillator encodings with discrete EsT operations.

## Discussion

In this work, we design an error semi-transparent (EsT) operation with simple linear drives leveraging dynamic subspaces to have universal control on a binomial bosonic logical qubit. We show that trading off exact ET for simpler control can enable substantial performance improvement for actively manipulated bosonic quantum information. Our design framework for EsT gates can be readily extended to other rotation-symmetric codes, such as four-legged cat codes^5,44^ and higher-order binomial codes^33^. Our optimization framework also provides a pathway to explore the trade-offs for an approximate Eastin-Knill theorem^45^ in standard qubit architectures and construction of fast, approximately bias-preserving operations for erasure qubits^46,47^.

However, because the linear drive may not fully mitigate the nonlinear self-Kerr and cross-Kerr effects, which do not commute with the photon loss operation on the codespace, an exactly ET operation is not likely to be achievable. In Supplementary Section I, we describe further implications of using a purely linear drive. Improvements in ET performance can also be obtained with optimization for additional objectives of minimizing mean photon number difference of evolving code words and leakage (see Supplementary Section I for details). An optimization involving dynamics obtained from solving the master equation, taking into account the loss rates of the system, will be able to account for the probability of photon jump and no-jump errors and therefore show further performance improvement. One could leverage dynamic subspace ET construction and obtain a theoretically exact ET with strong nonlinear drives (such as squeezing) using additional or different circuit elements. One possible Hamiltonian engineering technique for achieving exact ET with nonlinear drives in the adiabatic limit has been described in Ref. ^23^. However, the utility of such schemes must be balanced with the increase in circuit and protocol complexity that potentially leads to more error channels.

The remaining dominant error source in the circuit is the decoherence of the transmon ancilla. Historically, the ancilla has had higher decoherence rates than the oscillator. However, due to the use of higher oscillator levels during a fast amplitude-mixing gate, the effective error probability during the gate is comparable for both the oscillator and the qubit as measured in experiments (Supplementary Section V) and validated by simulations (Supplementary Section IV). In principle, qubit errors could be mitigated by replacing the qubit drives with a few simultaneous off-resonant Photon-Assisted Stark Shift (PASS) drives^20^, optimizing their amplitude to impart the necessary geometric phase to the cavity states without exciting the ancilla. However, this approach necessitates inefficiently long gate times to maintain adiabaticity or risks higher decoherence rates and ancilla population with stronger drives. Alternatively, a multi-level ancilla could be utilized to numerically enforce ancilla path independence alongside oscillator error transparency^18,21,48^. Utilizing the *g*-*f* transition of the ancilla for control would facilitate the detection of ancilla relaxation errors by monitoring the leakage to the state $\left.| e\right\rangle$, thereby heralding ancilla errors^49,50^. Such heralding of error is advantageous as it converts generic errors into erasure errors with known location.

In summary, we have introduced the concept of dynamic subspaces as a framework for realizing Error semi-transparent (EsT) operations. By traversing beyond static code and error spaces, this approach enables fast universal control of bosonic encodings using simpler linear drives. Our results demonstrate that EsT operations significantly enhance the fidelity of actively controlled bosonic qubits. The EsT gate set can be readily used for enhancing circuit depth for sensing and algorithmic applications and quantum signal processing^22^. Our approach is also compatible with methods for ancilla error detection ^49,50^, offering a practical pathway towards error-mitigated universal control of bosonic quantum information.

## Methods

### Pulse optimization

We optimize the complex drive amplitudes *ϵ*(*t*) and *Ω*(*t*) using GRAPE with 1 ns time steps. The cost function simultaneously optimizes for higher fidelity operation and high error transparency in the dynamic subspace with appropriate weights. Physical hardware constraints are also imposed by restricting the maximum drive amplitudes to 4 MHz and a 50 MHz bandwidth limit. Further, we enforce a 48 ns Gaussian rise and fall duration of the pulse to ensure smooth ramp-on and ramp-off of the pulse from and to zero drive amplitude.

### Experimental details

The logical qubit is encoded in a 3D *λ*/4 aluminum cavity coupled with a transmon, made with Nb capacitor pads and an Al/AlO_*x*_/Al Josephson junction, with its on-chip readout resonator.

For process tomography, we prepare the qubit in four cardinal points of the Bloch sphere: $\{\left.| g\right\rangle,\left.| e\right\rangle,(\left.| g\right\rangle+\left.| e\right\rangle )/\sqrt{2},(\left.| g\right\rangle+i\left.| e\right\rangle )/\sqrt{2}\}$. A 600 ns-long encode operation then maps the qubit state to the logical Bloch sphere. After desired logical operations (or processes), another 600 ns-long decode operation is applied to map the logical Bloch sphere state to the physical qubit state. Then we measure the qubit in all three Pauli bases to perform qubit state tomography. From this, we obtain the 4 × 4 process matrix *χ*_*G*_. The fidelity is defined as the overlap of the process matrix with the target process matrix *χ*_target_ as *F* = tr(*χ*_*G*_*χ*_target_). The encode-decode process (which should be an identity operation) has a process fidelity of  ~ 91%.

The autonomous quantum error correction (AQEC) pulse was developed in Ref. ^20^. It is numerically optimized to perform the unitary operation *U* such that $U\left.| {\psi }_{{{{\rm{E}}}}}\right\rangle \left.| g\right\rangle=\left.| {\psi }_{{{{\rm{L}}}}}\right\rangle \left.| e\right\rangle \,,U\left.| {\psi }_{{{{\rm{L}}}}}\right\rangle \left.| g\right\rangle=\left.| {\psi }_{{{{\rm{L}}}}}\right\rangle \left.| g\right\rangle \,$. So the error entropy is transferred from the logical qubit to the ancilla qubit. The ancilla is then reset conditioned on the readout following the AQEC pulse. This reset has a latency of 300 ns for determining the qubit state based on the preceding readout.

Further details of the theory, pulse optimization, experimental setup, additional simulation and experimental data supporting the findings of this work, and error model for code and error space process fidelities are presented in the supplementary information.

## Supplementary information

Supplementary Information

Transparent Peer Review file

## Data Availability

Data that support the findings of this article are openly available in Ref. ^51^.

## Code availability

Code and analysis that support the findings of this article are openly available in Ref. ^51^.
