# Supplementary material for: Error semitransparent universal control of a bosonic logical qubit
Source: Nat Commun. 2026 Oct 8;17:11441. doi: 10.1038/s41467-026-78317-y (PMC13737002; doi:10.1038/s41467-026-78317-y)
Supplement: Supplementary file 1 [file 41467_2026_78317_MOESM1_ESM.pdf]

Error Semitransparent Universal Control of a Bosonic Logical Qubit

Saswata Roy,[1,*] Owen C. Wetherbee,[1,2] and Valla Fatemi[3,†]

[1] *Department of Physics, Cornell University, Ithaca, NY, 14853, USA*
[2] *Department of Physics, University of California, Berkeley, USA*
[3] *School of Applied and Engineering Physics, Cornell University, Ithaca, NY, 14853, USA*

Abstract

Bosonic codes offer hardware-efficient quantum error correction, recently achieving beyond-breakeven quantum memory. However, fault-tolerant logical operations remain a critical bottleneck. While error transparent (ET) gates offer a pathway to resolve this, experimental demonstrations remain limited to phase gates. Here, we introduce a dynamic encoding subspaces framework that enables simple linear drives to accomplish universal logical gates that are error semi-transparent (EsT) to oscillator photon loss. Our EsT logical gate set of $\{X, H, T\}$ exhibited a five-fold reduction in infidelity conditioned on photon loss, extended active-manipulation lifetimes with quantum error correction, and enabled a composite EsT non-Clifford operation using an eight-gate sequence. Our approach is compatible with methods for detectable ancilla errors and is readily extensible to other rotation symmetric codes, offering an approach to error-mitigated universal control of bosonic logical qubits with the standard quantum control toolkit and a coherent framework for incorporating more sophisticated quantum control methods.

Supplementary Information

I. THEORY

Implications of linear drive: Here we analyze the implications of using purely linear drives, specifically the obstructions to their achieving gates ET to oscillator photon loss in the presence of self-Kerr and cross-Kerr effects. In the dispersive regime, the coupled cavity-qubit Hamiltonian when driven can be written as

$$H(t) = H_c + H_q + H_{\text{int}} + H_{\text{drive}}(t) \ , \tag{1}$$

$$H_a/\hbar = \omega_a \hat{a}^\dagger \hat{a} + \frac{K}{2}(\hat{a}^\dagger)^2 \hat{a}^2 + \frac{K'}{6}(\hat{a}^\dagger)^3 \hat{a}^3 \ , \tag{2}$$

$$H_q/\hbar = \omega_q \hat{q}^\dagger \hat{q} + \frac{K_q}{2}(\hat{q}^\dagger)^2 \hat{q}^2 \ , \tag{3}$$

$$H_{\text{int}}/\hbar = \chi \hat{a}^\dagger \hat{a} \hat{q}^\dagger \hat{q} + \frac{\chi'}{2} \hat{q}^\dagger \hat{q} (\hat{a}^\dagger)^2 \hat{a}^2 \ , \tag{4}$$

$$H_{\text{drive}}(t)/\hbar = \epsilon(t)\hat{a} + \Omega(t)\hat{q} + \text{h.c.} \tag{5}$$

where, ω_a and ω_q are the angular frequencies and $\hat{a}$ and $\hat{q}$ are the corresponding lowering operators of the oscillator and the qubit, respectively; K is the self-Kerr nonlinearity of the oscillator, K' is the higher order self-Kerr of the oscillator, and K_q represents the anharmonicity of the qubit. The system interaction is characterized by the dispersive coupling strength χ and the higher-order coupling term χ'. Finally, $\epsilon(t)$ and $\Omega(t)$ denote the time-dependent complex drive amplitudes applied to the oscillator and qubit ports. Even though the higher order terms were not described in the main text, it is important to take them into account for an accurate description of the system.

Considering the full Hamiltonian in Eq. (1)-(5), we see that the commutator of terms like $\omega_a \hat{a}^\dagger \hat{a}$, $\omega_q \hat{q}^\dagger \hat{q}$ and $\chi \hat{a}^\dagger \hat{a} \hat{q}^\dagger \hat{q}$ with the oscillator photon jump operator ($\hat{a}$) are proportional to $\hat{a}$. Consequently, a photon loss event during evolution under these terms of the Hamiltonian maps the final logical state $|\psi_L\rangle$ to the corresponding error space state $\hat{a}|\psi_L\rangle$, with a global phase on the error space states that depends on the time of photon loss. More generally, any terms of the Hamiltonian whose commutators with $\hat{a}$ are correctable will map a photon loss event to a correctable error under evolution by those terms (this is known as error-closure [1], a generalization of error-transparency).

However, we identify two terms whose commutators with the photon loss operator ($\hat{a}$) are not correctable: the oscillator self-Kerr non-linearity ($K\hat{a}^\dagger \hat{a}^\dagger \hat{a} \hat{a}/2$) and the higher order dispersive shift ($\chi' \hat{a}^\dagger \hat{a}^\dagger \hat{a} \hat{a} \hat{q}^\dagger \hat{q}/2$):

$$\left[\frac{K}{2}\hat{a}^\dagger \hat{a}^\dagger \hat{a} \hat{a} + \frac{\chi'}{2}\hat{a}^\dagger \hat{a}^\dagger \hat{a} \hat{a} \hat{q}^\dagger \hat{q}, \hat{a}\right] = -(K + \chi' \hat{q}^\dagger \hat{q})\hat{a}^\dagger \hat{a}^2 \ . \tag{6}$$

The nonlinear terms K and χ' introduce an amplitude-dependent differential rotation between the code and error subspaces. This can somewhat be mitigated by an appropriate linear drive $\epsilon(t)$ at each time point, but can not be fully mitigated as the linear drive lacks the non-linear operations required to fully correct the shearing error.

Eq. (6) dictates a fundamental trade-off. To improve ET fidelity, we can engineer hardware with minimal K and χ' by decreasing the dispersive coupling χ of the cavity and the qubit. One can also minimize $\epsilon(t)$ and optimize the trajectory to lower intermediate photon number ($\bar{n}$) during the evolution. However, such weaker coupling and drive strengths necessitate longer gate duration (t_g), which increases the integrated probability of photon loss ($\kappa_c \bar{n} t_g$) and exposes the system to errors from other sources (e.g., qubit decoherence), where $\kappa_c = 1/T_{1,c}$ is the oscillator photon loss decay rate.

Possible improvements: Further the linear drive is insufficient to guarantee fidelity under deterministic no-jump evolution. In the Lindblad master equation, the continuous non-unitary back-action is governed by the

* Corresponding author: sr938@cornell.edu
† Corresponding author: vf82@cornell.edu

effective non-Hermitian Hamiltonian $\hat{H}_{\text{eff}} = \hat{H} - i\frac{\kappa_c}{2}\hat{a}^\dagger\hat{a}$. Because our code words span superposition of different Fock states, the state-dependent amplitude damping deterministically distorts the code space, even in the absence if discrete jump errors. This continuous distortion can be captured and optimized by simulating the full master equation or stochastic Schrodinger equation to improve performance of the gate operation. However, such pulse optimization is computationally more intensive than using the Schrodinger equation and remains vulnerable to experimental fluctuations in κ_c. These no-jump errors can also be addressed by expanding the ET error set to include the $\hat{n}$-type errors arising from the no-jump evolution if they are correctable within the encoding [2], but this is only relevant for larger codes which can strictly correct these higher-order errors.

A secondary limitation of our current protocol is that it does not directly minimize Δ_{QEC}. In the absence of this logical states will undergo amplitude damping at different rates. More importantly, during the photon loss, the environment can gain information about 'from which state the photon loss is occurring' and thereby dephase the logical information. This is an irreversible error that the standard ET protocol can not correct at the end of the operation. However, we can take this into account with another optimization objective in the optimal control problem and improve the gate fidelity. We can minimize Δ_{QEC} directly or specifically for the binomial 'kitten' code by minimizing $\Delta\bar{n}_L(t)$, defined as

$$\begin{aligned}\Delta\bar{n}_L(t) =& \langle 0_L|\hat{U}^\dagger(t,0)\hat{a}^\dagger\hat{a}\hat{U}(t,0)|0_L\rangle \\ &- \langle 1_L|\hat{U}^\dagger(t,0)\hat{a}^\dagger\hat{a}\hat{U}(t,0)|1_L\rangle\ .\end{aligned} \tag{7}$$

Similarly, minimizing the leakage out of instantaneous error subspace can also be added as an additional objective to improve error transparency of the operation.

QEC violation metric derivation: To quantify violation of the Knill-Laflamme condition in our dynamic code space, we adapt the quantum error-correction (QEC) matrix formalism used by Albert *et al.* in Ref. [3]. For a general error channel characterized by Kraus operators $\{E_k\}$, the Knill-Laflamme QEC conditions necessitate that the projection of any error product $E_i^\dagger E_j$ onto the code space is proportional to the logical identity. For a dynamic code space with instantaneous projector $P_C(t) = |0_C(t)\rangle\langle 0_C(t)| + |1_C(t)\rangle\langle 1_C(t)|$, the effect of these errors is captured by the 2×2 instantaneous QEC matrices:

$$M_{ij}(t) = P_C(t)E_i^\dagger E_j P_C(t)\ . \tag{8}$$

Because $P_C(t)$ serves as the identity operator within the two-dimensional logical subspace, $M_{ij}(t)$ can be uniquely expanded in the instantaneous logical Pauli basis $\{P_C(t), X_C(t), Y_C(t), Z_C(t)\}$:

$$\begin{aligned}M_{ij}(t) = c_{ij}(t)P_C(t) + x_{ij}(t)X_C(t) \\ + y_{ij}(t)Y_C(t) + z_{ij}(t)Z_C(t)\ ,\end{aligned} \tag{9}$$

where the complex coefficients are extracted via the trace inner product: $[c,x,y,z]_{ij}(t) = \frac{1}{2}\text{Tr}\{[P,X,Y,Z]_C(t)M_{ij}(t)\}$.

Exact error correction requires $M_{ij}(t) = c_{ij}(t)P_C(t)$ for all error combinations. The non-identity coefficients $x_{ij}(t)$, $y_{ij}(t)$, and $z_{ij}(t)$ quantify the magnitude of uncorrectable logical bit, phase, and joint bit-phase flips.

To construct a scalar metric $\Delta_{\text{QEC}}(t)$ that captures the total deviation from exact correctability, we isolate these uncorrectable parts. Because the logical Pauli matrices X_C, Y_C, Z_C are strictly traceless and $\text{Tr}[P_C(t)] = 2$, taking the trace of Eq. (9) yields $\text{Tr}[M_{ij}(t)] = 2c_{ij}(t)$. Subtracting $\frac{1}{2}\text{Tr}[M_{ij}(t)]P_C(t)$ from $M_{ij}(t)$ isolates the uncorrectable error generators:

$$\begin{aligned}M_{ij}(t) - \frac{1}{2}\text{Tr}\left[M_{ij}(t)\right]P_C(t) = x_{ij}(t)X_C(t) \\ + y_{ij}(t)Y_C(t) + z_{ij}(t)Z_C(t)\ .\end{aligned} \tag{10}$$

We quantify the magnitude of this uncorrectable component using the Frobenius norm, $\|A\|_2 = \text{Tr}(A^\dagger A)$. Utilizing the algebraic properties of the Pauli matrices, all cross-terms vanish under the trace. The norm evaluates directly to the sum of the squared error magnitudes:

$$\begin{aligned}\Delta_{\text{QEC}}(t) = \left\| M_{ij}(t) - \frac{1}{2}\text{Tr}\left[M_{ij}(t)\right]P_C(t)\right\|_2 \\ = 2\left(|x_{ij}(t)|^2 + |y_{ij}(t)|^2 + |z_{ij}(t)|^2\right).\end{aligned} \tag{11}$$

Summing this squared distance over all relevant error combinations i,j yields the total QEC violation metric $\Delta_{\text{QEC}}(t)$ presented in the main text.

For the bosonic codes, the relevant error generators are $\{\hat{a},\hat{n}\}$ which correspond to single photon loss in the oscillator and dephasing, backaction respectively. Thus the above condition can be summed for $\mathcal{O} \in \{\hat{a},\hat{n}\}$. Expressing the Pauli coefficients in terms of matrix elements $\mathcal{O}_{\mu\nu}(t) = \langle \mu_C(t)|\hat{\mathcal{O}}|\nu_C(t)\rangle$, the metric simplifies to the scalar summation

$$\Delta_{\text{QEC}}(t) = \sum_{\mathcal{O}\in\{\hat{a},\hat{n}\}} \left\| M_{\mathcal{O}}(t) - \frac{1}{2}\text{Tr}\left[M_{\mathcal{O}}(t)\right]P_C(t)\right\|_2 . \tag{12}$$

This formulation provides a computationally and analytically accessible cost function for bounding the QEC violation.

II. EXPERIMENTAL SETUP

Schematic design of the package is shown in Supplementary Fig. 1. In this section we describe the production and characterization of the package, with measured quantities shown in Supplementary Table. 1. This is the same package as used in Roy, *et al*[4] with minor changes in the cryogenic wiring.

Quantity	Symbol	Measured value	Hamiltonian term
Cavity frequency	$\omega_a/2\pi$	6.05 GHz	$\omega_c a^\dagger a$
Readout frequency	$\omega_r/2\pi$	8.92 GHz	$\omega_r r^\dagger r$
Qubit frequency	$\omega_q/2\pi$	5.60 GHz	$\omega_q q^\dagger q$
Qubit-readout dispersive coupling	$\chi_r/2\pi$	−600 kHz	$\chi_r r^\dagger r\|e\rangle\langle e\|$
Qubit-Cavity dispersive coupling	$\chi/2\pi$	−3.66 MHz	$\chi a^\dagger a\|e\rangle\langle e\|$
Qubit anharmonicity	$K_q/2\pi$	−180 MHz	$K_q q^\dagger q^\dagger qq/2$
Second order cavity-qubit dispersive coupling	$\chi'/2\pi$	39 kHz	$\chi' a^\dagger a^\dagger aa\|e\rangle\langle e\|/2$
Cavity Self-Kerr	$K/2\pi$	−22 kHz	$K a^\dagger a^\dagger aa/2$
Second order Cavity Self-Kerr	$K'/2\pi$	590 Hz	$K' a^{\dagger 3} a^3/6$
Qubit lifetime	T_{1q}	70 µs	
Qubit dephasing time, Ramsey	$T_{2q,R}$	30 µs	
Qubit dephasing time, Hahn-echo	$T_{2q,E}$	84 µs	
Cavity lifetime	T_{1a}	180 µs	
Cavity dephasing time	$T_{2a,R}$	290 µs	
Qubit thermal population		1.5%	
Cavity thermal population		0.9%	

Supplementary Table 1. **Device parameters:** Device parameters measured with standard spectroscopic and time-domain methods. The qubit has some TLS associated with it which makes it respond at two different frequencies about 50-60 kHz apart as seen in qubit spectroscopy and Ramsey experiments.

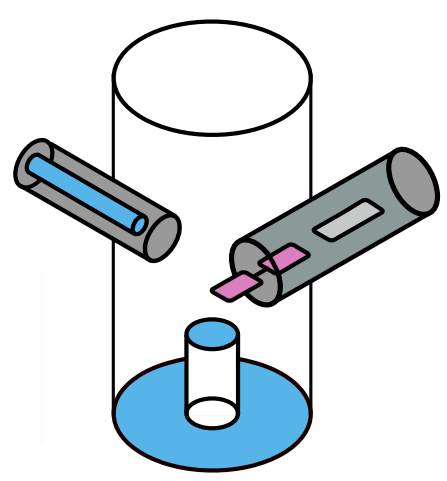

Supplementary Figure 1. **Schematic of the 3D package**: We use 3D $\lambda/4$ cavity made (light blue) of 4N Aluminum as the oscillator mode. An ancilla transmon qubit (pink) is coupled to the oscillator and used for controlling the oscillator. The transmon has a readout resonator (gray rectangle) coupled to it for readout of the transmon state.

We used a $\lambda/4$ cavity made of 4N Aluminum as the cavity mode [5] (harmonic oscillator) in which we encode our binomial logical qubit. The cavity was treated with an acid etch to remove surface impurities. While this process typically produces cavities with lifetimes approaching the millisecond scale, the surface appears to have degraded due to shipment, long shelf time exposed to air, resulting in the measured cavity lifetime of 180 µs.

The superconducting qubit used is a transmon with Nb capacitor pads, and an $Al/AlO_x/Al$ Josephson junction fabricated on a high-resistivity (> 10 kΩ cm) silicon chip. Following a strip of the native silicon oxide in 2% hydrofluoric acid, a 75 nm thick Nb film was sputtered at the rate of 50 nm min^{-1}. This Nb was patterned using photo-lithography to make the capacitor pads of transmon and the readout resonator. The Josephson junction for the qubit was fabricated in a Dolan bridge process [6] with bilayer MMA/PMMA resist. Double-angle evaporation for the Al-AlO_x-Al was accomplished to form the junction. Before the first Al deposition an in-situ ion mill was performed to clean the top surface of Nb. After the first Al deposition, AlO_x layer was formed by oxidizing the Al surface for 30 minutes at 7.25 Torr oxygen pressure. After pumping out the oxygen from the chamber, the second layer of Al was deposited . The area of the deposited Josephson junction is 0.047 µm^2. The chip with the transmon qubit and readout resonator is mounted in the 3D cavity with a copper clamp .

The whole package is mounted on an oxygen free high conductivity (OFHC) copper bracket and has multiple layers of shielding, in order from inner to outer: (1) a Berkeley Black [7] coated copper shim to absorb any stray mm-wave radiation, (2) an aluminum can with an indium seal to an upper flange, which itself has indium-sealed SMA feedthroughs, (3) a mixing chamber can. This is depicted schematically in Supplementary Fig. 2. The dilution fridge also hosts a room-temperature magnetic shield that lines the vacuum can.

The control pulses are generated using Quantum Machines OPX plus instrument and up-converted using local oscillator (LO) and IQ mixers in the Quantum Machines Octave. All pulses are digitally triggered with 100 ns buffer on the trigger on either side of the analog pulse. The qubit and storage drives share the same LO whereas the readout up and down conversion share the same LO. Output of the readout signal is first amplified with a Travelling Wave Parametric Amplifier (TWPA) at base temperature, followed by a High electron mobility transistor (HEMT) at 4 K and a room temperature amplifier (ZVA-1W-103+ Mini-Circuits). The TWPA is also pulsed with 100 ns buffer on both ends of the readout pulse. Each of the input lines have K & L low pass filter and Eccosorb filters. All the last attenuators, microwave filters, Eccosorb and HERD filters are connected to the copper

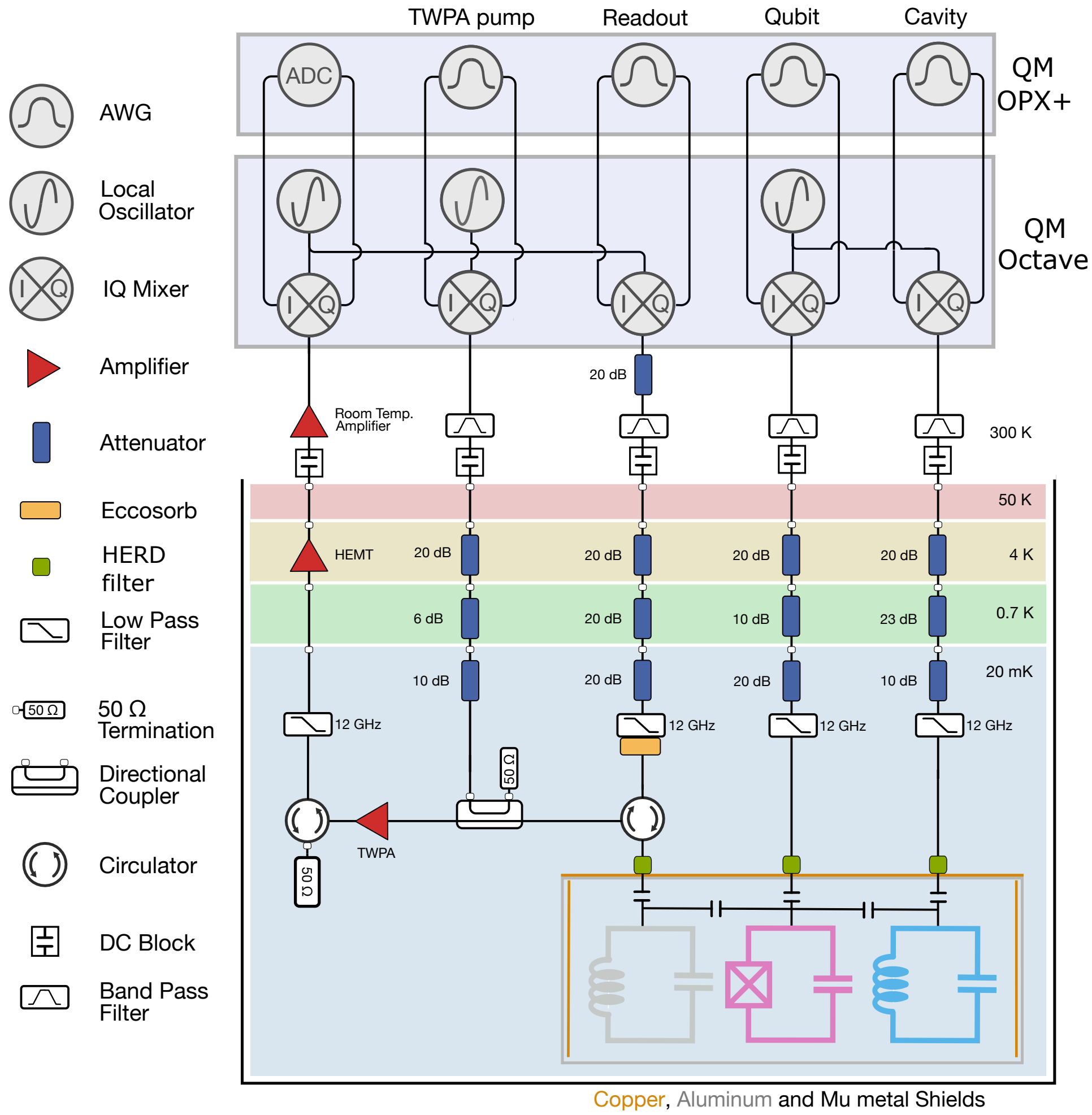

Supplementary Figure 2. **Experimental wiring diagram**: Schematic of room temperature and cryogenic microwave circuit.

bracket using a copper braid to improve thermalization.

III. PULSE OPTIMIZATION METHOD

For achieving any operation on the joint cavity-qubit system, we optimize the complex time-dependent drive amplitudes $\epsilon(t)$ and $\Omega(t)$ that is simultaneously applied to the cavity and the qubit on resonance. We simulate the system dynamics governed by the driven Hamiltonian $H(t)$ by numerically integrating the Schrodinger equation using the Dynamiqs package [8]. The pulse shapes are then optimized via the Gradient Ascent Pulse Engineering (GRAPE) method [9], utilizing JAX [10] for automatic differentiation and the Adam optimizer [11] from the Optax library [12] for gradient-based parameter updates.

We define a few different cost functions and use a single net weighted cost that the optimizer tries to minimize for parameter update. For easy computation of some cost functions, we simulate the evolution of states instead of simulating the propagator under the Hamiltonian. We start from six cardinal points in the Bloch sphere, evolve them and calculate fidelities and other costs based on the evolved state. We are interested in achieving high fidelity for all our operations. So starting from a set of initial states $|\psi_L\rangle$, we evolve the states under the time dependent Hamiltonian $H(t)$ to get the final states $|\psi_{\text{final}}\rangle$ and then calculate the overlap fidelity with the target set of states. The cost function is defined as the infidelity

$$C_1 = 1 - F_{\text{target}} = 1 - \frac{1}{6}\sum_{i=1}^{6} |\langle\psi_{\text{final},i}|\psi_{\text{target},i}\rangle|^2 \ , \quad (13)$$

where the sum is over the number of states used in the simulation. We use the six cardinal points of the logical Bloch sphere in our simulation.

Next, we consider enforcing dynamic error transparency. For this, we construct a second cost function based on the intermediate trajectory of the logical states based on the theory in Supplementary Sec. I. We evolve our states starting from cardinal points in the code and the error space, denoted by $\psi_C(t_0)$ and $\psi_{E_j}(t_0)$ respectively, where $t_0 = 0$. Constrained by the temporal resolution of the control hardware and computational overhead for

optimization, we discretize the evolution generate by $H(t)$ into N steps of resolution $\Delta t = t_n - t_{n-1} = 1\,\mathrm{ns}$. We evaluate the instantaneous states $\psi_C(t_n)$ and $\psi_{E_j}(t_n)$ at the n-th time step by solving the Schrodinger equation with $H(t)$. We define a per step normalized ET fidelity metric using these evolving states as

$$F_{\mathrm{ET}} = \frac{1}{N}\sum_{n=0}^{N-1}\frac{|\langle\psi_{E_j}(t_n)|\hat{a}|\psi_C(t_n)\rangle|^2}{\mathcal{N}}\ , \tag{14}$$

where, $\hat{a}$ is the bosonic annihilation operator and $\mathcal{N}$ is the normalization factor, $\mathcal{N} = \sqrt{\langle\psi_C(t_n)|a^\dagger a|\psi_C(t_n)\rangle}$. Note that calculating ET fidelity this way is equivalent to partially satisfying all three ET condition described in Eq. (3)-(5) in main text. This per step ET fidelity is then averaged over six set of starting points $(\psi_C(t_0), \psi_E(t_0))$ corresponding to cardinal points of the logical Bloch sphere. We call this $F_{\mathrm{ET,\ avg}} = \frac{1}{6}\sum_{i=1}^{6} F_{\mathrm{ET,\ i}}$. Then the corresponding cost function is written as

$$C_2 = 1 - F_{\mathrm{ET,\ avg}}\ . \tag{15}$$

Eq. (13) and (15) are the two main cost functions used in this work. For some initializations of $\epsilon(t)$ and $\Omega(t)$, we had to use a regularizer cost function to ensure that the optimizer does not simply minimize the pulse dynamics duration to artificially inflate F_{ET}. For this, we can calculate the Fubini-Study distance (FSD) metric between consecutive steps, which is defined as

$$\mathrm{FSD} = 2\arccos|\langle\psi_1|\psi_2\rangle|\ , \tag{16}$$

with $|\psi_1\rangle$ and $|\psi_2\rangle$ being the two states we want to calculate the distance between. We can then define a velocity metric (v_n) using the Fubini-Study distance between two states at two consecutive states during evolution of our driven system, i.e., between $|\psi_C(t_n)\rangle$ and $|\psi_C(t_{n+1})\rangle$,

$$v_i = (2/\Delta t)\arccos|\langle\psi_C(t_n)|\psi_C(t_{n+1})\rangle|\ , \tag{17}$$

We can then calculate the variance of this metric,

$$\sigma^2 = \frac{1}{N}\sum_{n=0}^{N-1}\left(v_n - \left(\frac{1}{N}\sum_{n=0}^{N-1} v_n\right)\right)^2\ , \tag{18}$$

Informally, we refer to this as 'velocity variance' as this is the variance of a metric that calculates a distance per unit time. This metric is then averaged over six cardinal points as the initial state. We define the cost function as

$$C_3 = \frac{1}{6}\sum_{i=1}^{6}\sigma_i^2\ . \tag{19}$$

This cost function ensures there is some dynamics at each step and thereby incentivizes the optimizer to not minimize the effective pulse duration. This regularizer is a convenient way to ensure that operations of different types (e.g., EsT and Ord) have similar duration so that the opportunity for incoherent errors to influence the operation is comparable. We also note that this cost function does not improve ET performance directly, and we acknowledge that other cost function metrics could accomplish a similar objective.

Finally we combine the cost functions with appropriate weights w_i (manually tuned) to feed a net cost function for the optimizer to minimize. This is done as

$$C_{\mathrm{tot}} = \sum_k w_k C_k\ . \tag{20}$$

We do the optimization at two stages. A first stage optimization is performed with weights typically as $\vec{w} = (w_1, w_2, w_3) = (1, 0.6 - 0.75, 5 - 10)$ for fidelity, ET and velocity variance. This allows us to reach ET fidelity of up to 0.83 and fidelity in the $0.9 - 0.99$ range. Beyond this a second stage optimization with weights $\vec{w} = (1, 0.1, 0)$ are used to maximize the fidelity of the operation. At this step we notice ET fidelity and velocity variance change at around 1% and less than 1%, respectively. We note that this weight optimization is crucial for obtaining EsT pulse with high gate fidelity and simultaneously high ET fidelity. In future works, finding the optimal weights can also be made part of the optimization process.

We also add additional restrictions on the pulse shape. Due to the finite bandwidth of our arbitrary wave generator, we Fourier transform the pulse and remove any frequency components beyond the bandwidth of 50 MHz. This helps to keep the pulses reliable and provide good matching of the simulation and experimental results. We also employ a maximum drive strength of $\Omega_{\mathrm{max}}/2\pi = \epsilon_{\mathrm{max}}/2\pi = 4\,\mathrm{MHz}$. Further, we enforce a 48 ns gaussian rise and fall duration of the pulse to ensure smooth ramp on and ramp off the pulse from and to zero drive amplitude.

By discretizing the control fields into 1 ns piecewise-constant intervals and applying these optimization methods, we obtain EsT gates with cost function in Eq. (20) and Ord gates with cost function defined in Eq. (13). X and H gates are accomplished in 1 µs by simultaneously driving the qubit and cavity on resonance whereas, T gate is achieved in 0.6 µs by driving the qubit only. Eq. (15) partially optimizes all three dynamic ET conditions described in Eq. (3)-(5) in main text. This cost function was used for its computational simplicity in gradient calculation and the minimal weight tuning required within multi-objective optimization framework used.

We can also use additional cost functions: (a) one that minimizes the difference of average photon number $(\bar{n})$ between the evolving codewords to satisfy the dynamic QEC condition; one that enforces the equality of instantaneous and evolved error spaces; (b) one that keeps the pulse shapes smooth; (c) one that improve the robustness of the pulse to parameter and drive amplitude fluctuations. These can be employed in the same framework, but careful tuning of the weights is needed to achieve desired goals. The weights can also be included as something to optimize but that requires a more sophisticated optimization

method. Techniques like projecting conflicting gradients, and conflict aware gradient optimization techniques may also be used for better optimization results. Further using multi-level ancilla, fault-tolerance with respect to ancilla errors can be similarly defined [2, 13, 14] and included in the optimization directly. These will be explored in future works.

A. LE gate

In the main text we have described a standard non-ET gate (named Ord) gate, that is optimized for only the fidelity of the operation in the code space. The EsT gate then optimizes for fidelity of operation in both code and error space, and also for error transparency. In between these two optimization schemes, we can optimize for another operation where the fidelity of the operation in code and error space is maximized but the ET condition is not enforced. We name this LE gate. It is expected that this gate will have a better error space gate fidelity F_{err} (as it is optimized for that) but will have worse fidelity error transparency for the case of photon loss during operation. So the performance of this gate will be somewhere between the Ord and EsT gate. This is verified by simulation in Supplementary Sec. IV and experimental results shown in Supplementary Sec. V for X gate.

B. AQEC pulse

Autonomous quantum error correction (AQEC) is equivalent to standard QEC consisting of error detection and correction operations. AQEC is designed to remove the parity measurement and related errors and rely on simpler qubit projection. The pulse is designed to be an unitary operation that maps error space state $|\psi_E\rangle$ to codespace and transfers the entropy to ancilla excitation and keeps the code space state $|\psi_L\rangle$ undisturbed [15].

$$U|\psi_{\mathrm{E}}\rangle|g\rangle = |\psi_{\mathrm{L}}\rangle|e\rangle \ , \tag{21}$$

$$U|\psi_{\mathrm{L}}\rangle|g\rangle = |\psi_{\mathrm{L}}\rangle|g\rangle \ . \tag{22}$$

After applying this pulse, the qubit state is measured and a conditional unselective pi pulse is applied to bring the qubit in ground state unselective of the oscillator state. This readout creates a phase difference between the code and error space which is taken into account in the pulse. We also take into account the evolution to self-Kerr, higher order self-Kerr, higher order dispersive shifts for the duration of the readout and ancilla reset to have a correct state at the end of the operation. We have numerically optimized the pulse shapes of drives simultaneously applied on the cavity and qubit to accomplish AQEC pulse in 1 µs. The pulse duration was chosen to be the shortest to minimize coherence related errors and control errors (that increase for shorter drive durations because of high amplitude, broad bandwidth pulses and complex dynamics of the system).

We can improve the performance of AQEC pulse by improving the following aspects: (1) Shorter readout with lower photon numbers by improving the coupling of the readout and the qubit, (2) Readout pulse shaping, such as CLEAR pulse [16], that decays all photon out of the resonator shortening the effective duration of active reset, (2) Adjusted χ of qubit and cavity that balances gate speed and finite bandwidth issues of the short pi pulse, (3) Improving on control errors with reinforcement learning based hardware-feedback controls [17, 18], (4) improving coherence times of the cavity and qubit and (5) Employing other autonomous error correction methods [19–22].

C. Encode-Decode operations

We designed encode operations that take any state in the qubit Bloch sphere to logical code space Bloch sphere. This pulse is used to create logical code space states. We also design decode operations that take logical Bloch sphere states to qubit Bloch sphere. Then the qubit state can be probed to generate the density matrix of the state and full process tomography can be performed in this way. For probing code and error space processes, we generate two different decode pulses for code and error space. These pulses are 0.6 µs long.

IV. SIMULATION RESULTS

In this section, we present additional results from numerical simulations that support the experimental data and provide more insight into the system dynamics. In Supplementary Fig. 3, we plot the QEC violation, leakage and trajectory mismatch conditions defined in Eq. (6), (7), (8) in main text for the EsT, LE and Ord X (a, b, c), H (d, e, f), T (g, h, i) gates. Leakage and trajectory mismatches are calculated for starting in code space state $|0_L\rangle$ for the X gate and for starting in code space state $|+Y_L\rangle$ for the H and T gates. The plot shows that the violation of these metrics by the LE gate is lower than the Ord gate but higher than the EsT gate. This similar hierarchy in fidelity for the three types of gates is observed in Supplementary Fig. 10. Further, the T gate shows no violation in QEC as the code states never go outside of the static code space. Shorter duration of the T gate combined with this no QEC violation makes it the best performing gate in this work.

In Supplementary Fig. 4, we present another comparison of the three types of X gate, by evaluating final state fidelity conditional on a photon loss error occurring at time t. We compute this by evolving an initial state to time t with Schrodinger equation, apply the photon loss operator and evolve the resulting state for the remaining pulse duration. The final fidelity with respect to the target state in the error space is then averaged over the six cardinal state in code space as our initial states. As

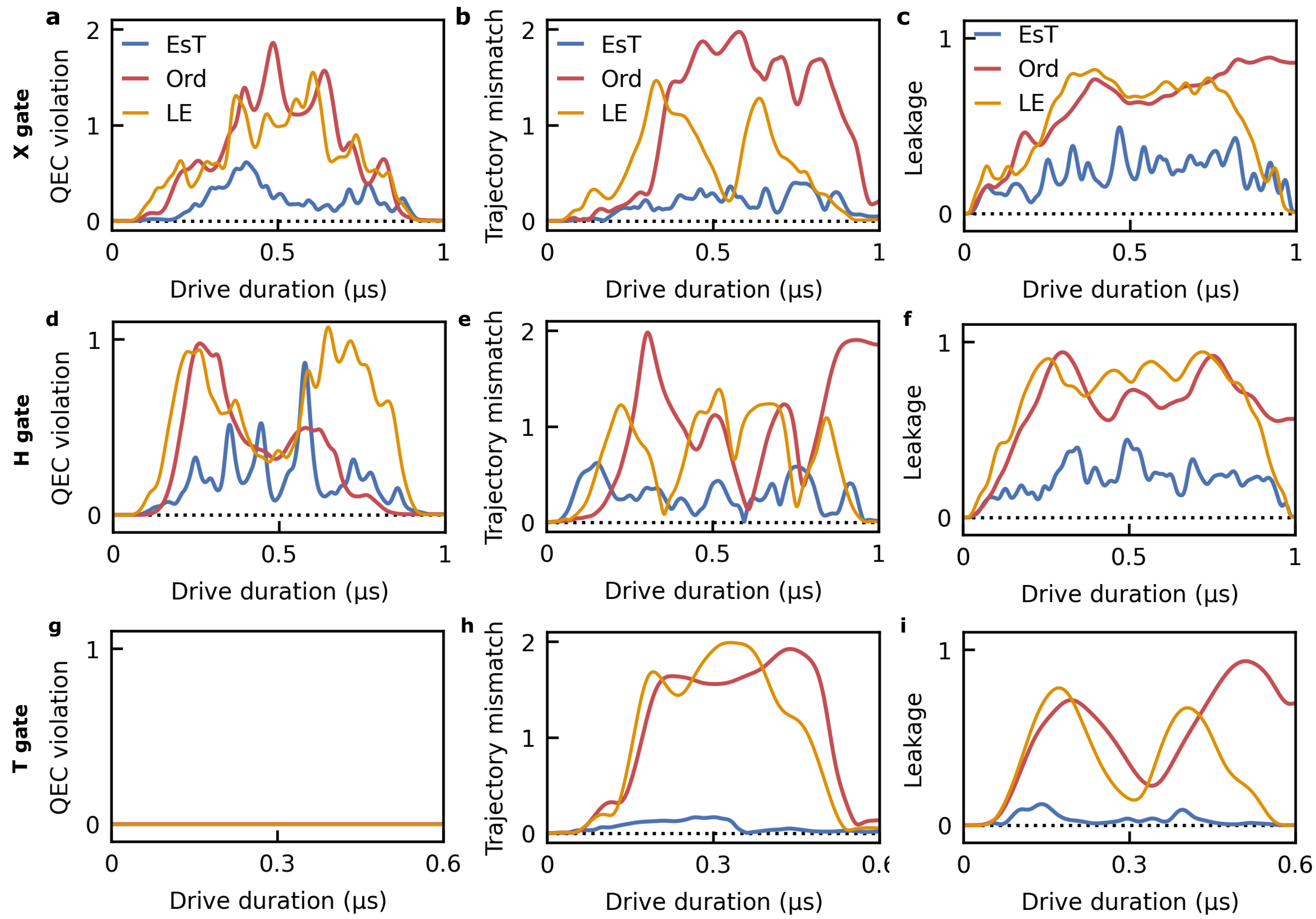

Supplementary Figure 3. **EsT, LE and Ord gate comparison:** QEC violation, leakage and trajectory mismatch in instantaneous subspaces are calculated from lossless simulations for EsT, LE and Ord X, H and T gates. The EsT and Ord gate metrics for the X gate are the same from Fig. 1d-f in main text, and are plotted again for ease of comparison with LE gate. For X gate, these metrics are plotted for an initial state $|0_L\rangle$, for H and T gates, these metrics are plotted for an initial state $|+Y_L\rangle$. T gate has the least violation of these metrics. Specifically, QEC violation is zero for T gate because it always stays in the codespace.

expected we observe average infidelity follows the hierarchy of Ord > LE > EsT. The infidelity also decreases for errors occurring later in the pulse, since a larger fraction of the gate undergoes ideal evolution.

We simulate the H gate operation with our device parameters and losses with a master equation solver and plot fidelity of the gate operations in code space and net fidelity after application of an ideal QEC operation with varying parameters in Supplementary Fig. 5. We observe that EsT gate is more sensitive to dispersive shift (χ) fluctuation but less sensitive to qubit frequency (ω_q) fluctuation compared to Ord gate. This is only specific to the pulse used in our experiment - other pulses starting from different seeds may have different robustness. In the future, robustness can also be included in the optimization as an additional cost function [23, 24].

We also present the optimized pulse shapes and dynamics for the Ord and EsT gate in Supplementary Fig. 6a and 6b respectively. The dynamics shows the probability of occupation of different Fock levels when starting from $|0_L\rangle$. The pulse shape and dynamics during the EsT gate is more complicated than the Ord gate. But both gates achieve the same final state and same maximum active Fock state level with transient population greater than 0.01.

In Supplementary Fig. 7a we present the pulse schematic used for the experimental sequence shown in Fig. 4 in main text. We simulate this sequence to obtain fidelity as a function of number of gates applied before AQEC operation to evaluate the performance in absence of control errors. The simulation results for three types of X gate are shown in Supplementary Fig. 7c. This validates our experimental observation the EsT gate significantly outperforms the Ord gate. Further, the LE gate yields an intermediate performance, outperforming Ord gate but not performing as good as the EsT gate.

Supplementary Fig. 7b shows a pulse sequence where an AQEC operation is applied after each gate. The simulation results for three gates, using experimental parameters, are shown in Supplementary Fig. 7d. The absolute fidelity decays rapidly, primarily due to the infidelities of the AQEC pulse itself. However, the relative per-gate fidelity improvement for the EsT gate is higher than the Ord gate.

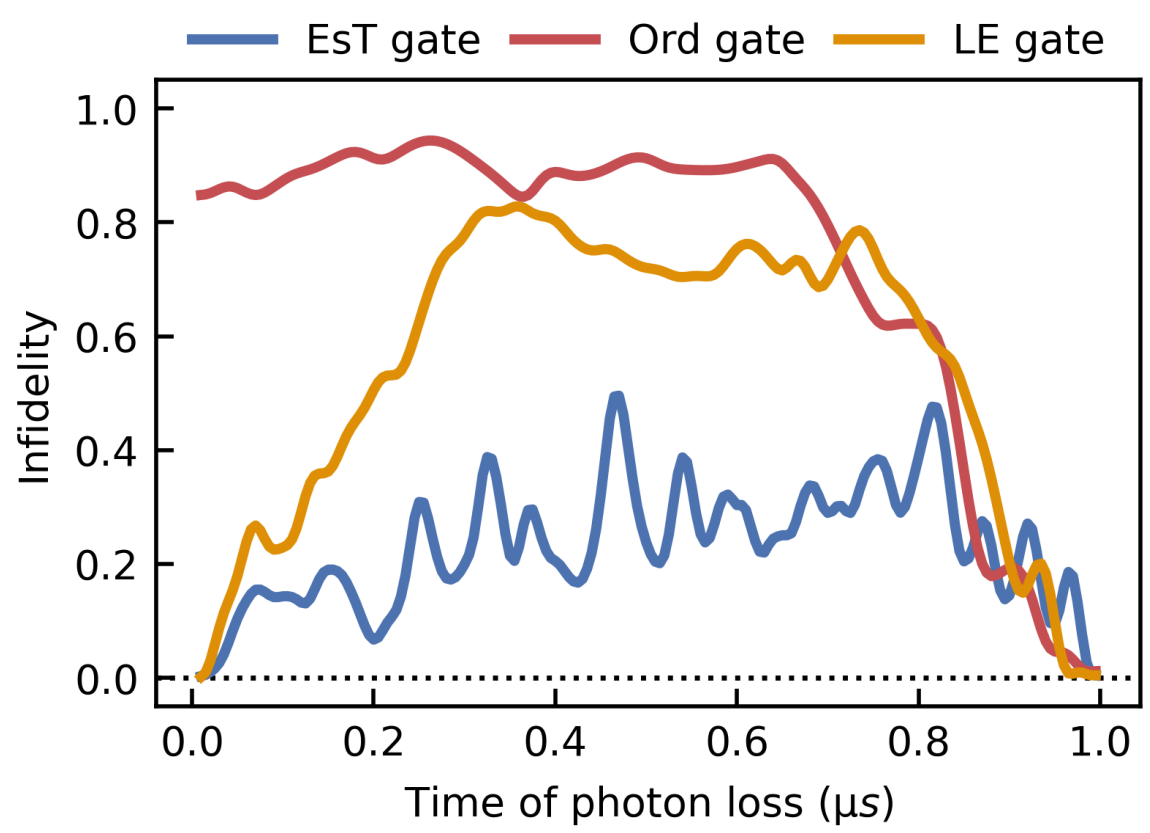

Supplementary Figure 4. **Gate fidelity for errors at a specific time:** We plot the infidelity in the error space at the end of the operation if a single photon loss happens at a specific time during the gate. Final infidelity is plotted as a function of this time of photon loss for the X gate. We see that EsT gate has lowest infidelity owing to the ET and error space fidelity optimization. Ord gate performs worst and LE gate performance is in between Ord and EsT gates.

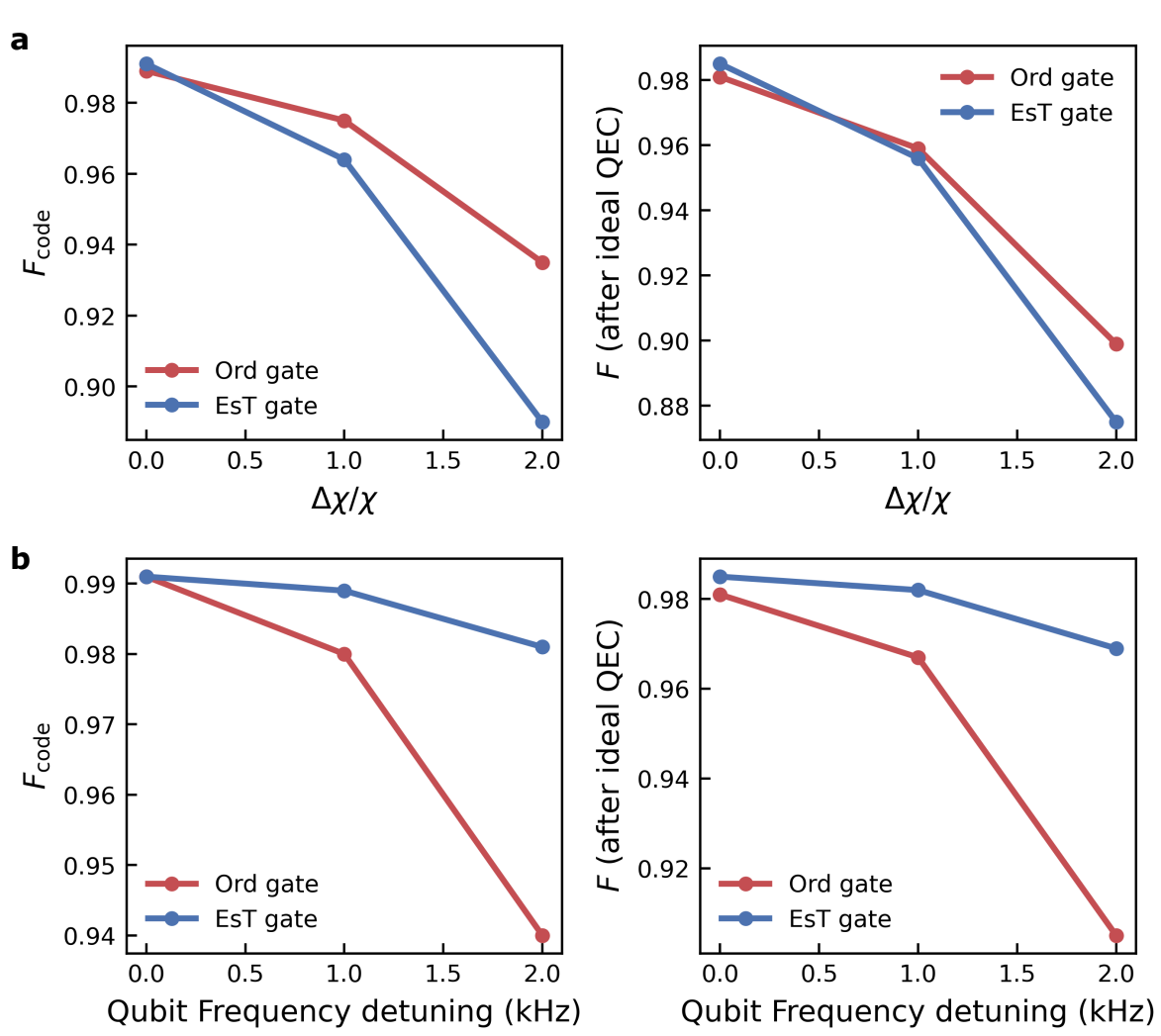

Supplementary Figure 5. **Robustness comparison:** We compare the robustness of EsT and Ord H gate by plotting fidelity with variation of (a) the dispersive coupling χ and (b) Qubit frequency detuning. Left column shows the fidelity of the operation in code space and the right side shows the fidelity of the operation after application of ideal QEC. EsT gate is more sensitive to χ variation but less sensitive to qubit detuning variation.

Finally, we isolate the sources of error in our gate operations using numerical simulation and models fitted to experimental data. The X and H gate fidelities for various scenarios are summarized in Supplementary Table. 2. We observe excellent agreement between simulated and experimentally extracted fidelities. The slight mismatch primarily arises from control errors and Hamiltonian parameter fluctuations. Below we point a few key observations from the table. Fidelities calculated without qubit errors are similar to those without oscillator errors, indicating that both loss channels are significant limits to gate performance. There is a clear enhancement in ET fidelity, validating our claim that error-transparency optimization substantially improves overall gate performance. We note that the experimental number for error space fidelity is extracted from the fit to experimental data using our model. If the fidelity of operation in error space was extracted from a process tomography experiment of the gate in error space, it may slightly differ from experimental value noted in this table.

To further understand the effect of qubit T_1 and T_2 errors, we simulate the fidelities of EsT and ordinary gates, taking into account each possible combination of errors. The simulation is performed with a master equation solver and the results are summarized in Supplementary Table. 3. From these results, we conclude that ancilla T_1 contributes slightly more to the gate infidelity than ancilla T_2, but their contributions are of the same order of magnitude for our device coherence times.

For comparison across coherence times or other schemes, we can define a simplified average fidelity by averaging code and error space fidelities (for error space we consider the fidelity in error space when error has happened during the gate and the state ends up in error space) weighted by the probabilities of being in those spaces. With simulations, we verify that EsT gate has a better average gate fidelity compared to Ord gate for a range of coherence times for qubit T_{1q}, T_{2q} ranging from 50 to 1000 μs and oscillator T_{1c}, T_{2c} ranging from 200 to 2000 μs.

Below we provide a brief summary of results from previous works on ET and path independent phase gates on binomial code and qualitatively compare them with this work. Ref. [15] implemented ET $R_z(\theta)$ (where, θ is dictated by gate duration) and Ref. [13] implemented $R_Z(\pi/2)$ operation. It is not the same as the T gate implemented in this work, but we can expect different phase gates to have qualitatively similar performance for a specific implementation. Besides, the coherence times for these works are also very different. So we can not make a comparison with these schemes directly. Nevertheless we compile some of the relevant numbers here. The ET Z gate was 120 μs long in Ma *et al* with a fidelity of 0.78 in code space and 0.68 in error space. The path-independent gate was greater than 2 μs long with net fidelity of 0.976. Our T gate is 600 ns long, and has measured fidelities of 0.988 in the code space and 0.85 in the error space if photon loss happens during the operation. This results in a net fidelity of 0.983 (average of code and error space fidelities weighted by the probability of being in the respective subspace). For the coherence times reported in the previous work, we also obtain fidelities of 0.993 and 0.994 for our T gate in simulations done with a master equation solver taking into account all measured device parameters and loss rates. Noticeably, the gate length in this work is shorter because there is no requirement of adiabaticity for the EsT scheme, compared to previous

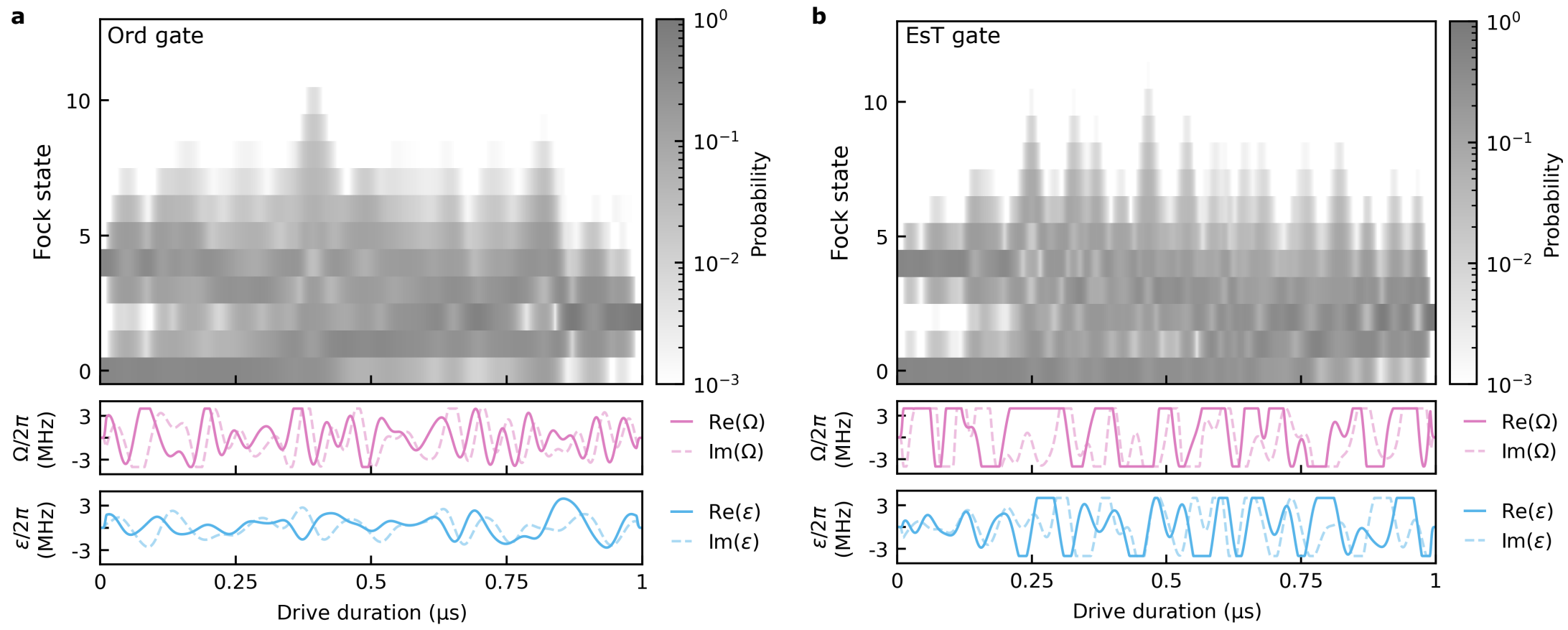

Supplementary Figure 6. **Dynamics in the driven oscillator:** This plot shows the dynamics of the oscillator during X gate on the binomial code and the optimized pulse shapes applied on the qubit and oscillator. (a) It shows the occupation of Fock states during the operation as color for Ord gate where gray means occupied and white means unoccupied. Bottom two panels show the complex amplitudes applied on qubit (top) and oscillator (bottom). (b) This plot also shows the oscillator Fock state occupation and complex drive amplitudes for the EsT gate.

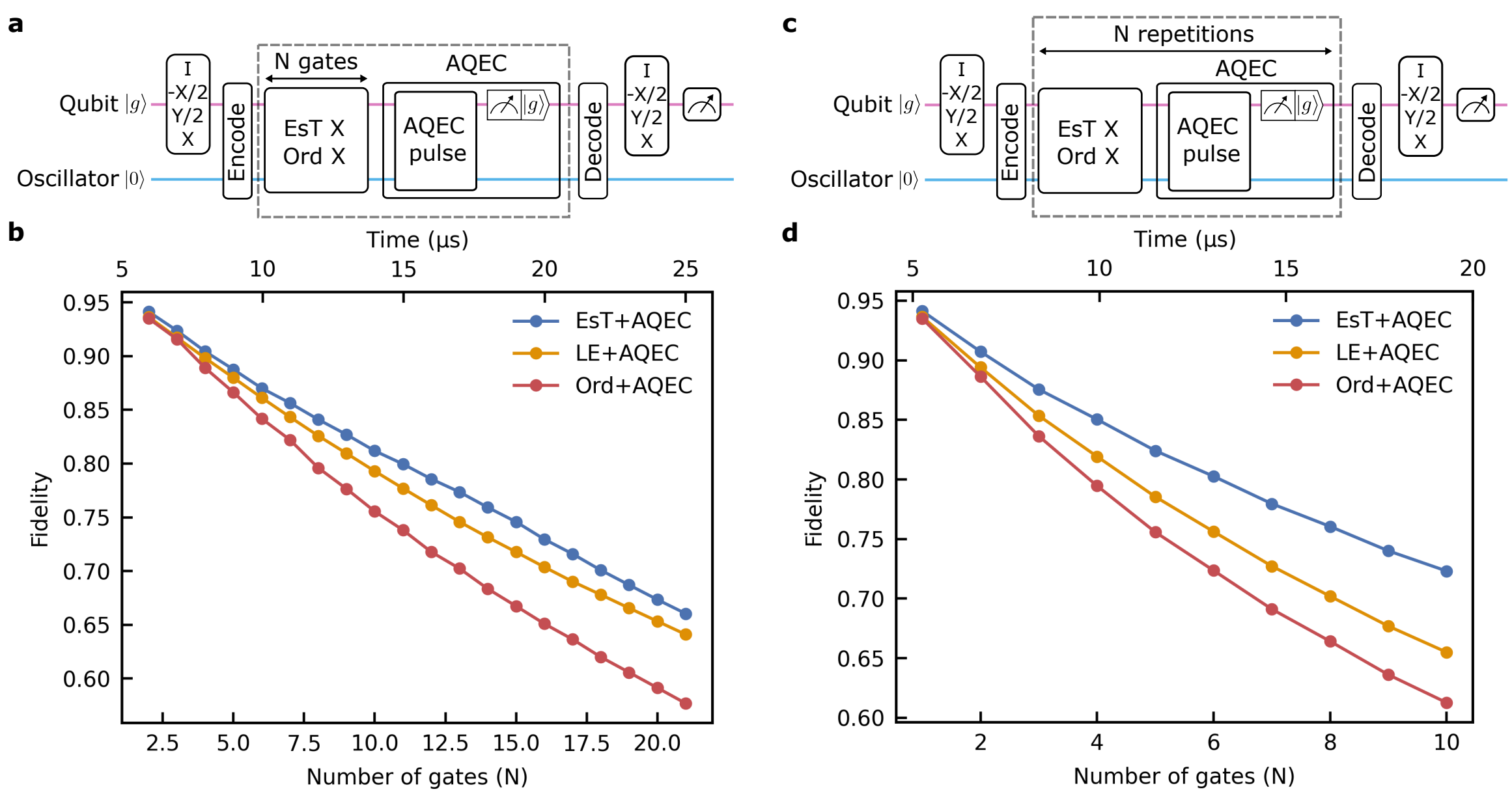

Supplementary Figure 7. **Gate performance with AQEC:**(a) Pulse schematic used for simulation results in (b). We apply N gates before applying a single AQEC pulse. (b) This plot shows the performance scaling for EsT, LE, Ord gates where AQEC pulse is applied once after N gates. This plot uses coherence parameter and pulses used in our experiment. (c) Pulse schematic used for simulation results in (d). We apply an AQEC pulse after each gate. (d) This plot shows the performance scaling for EsT, LE, Ord gates where AQEC pulse is applied after each gate. This plot uses coherence parameter and pulses used in our experiment. These results are a proof of performance improvement of EsT gate without control errors. The dashed square boxes in the pulse schematic are the pulses that are simulated with losses for obtaining the fidelity decay plots.

works where gate implementations required adiabaticity to maintain ET or path independence.

We also try to estimate sources of error for the encode-decode process and AQEC pulse with help of a master equation solver. We find that incoherent errors coming from the oscillator and qubit losses degrade fidelity by 4.5% . About 2.3% error comes from state preparation (where 1.6% error contribution comes from the thermal population in qubit and oscillator and about 0.7% comes from qubit state preparation pulse). The readout has about 1.2% assignment error. The remaining 1% error is attributed to control errors.

For the AQEC pulse, 5% error comes from oscillator and qubit losses and 2% from active reset process (readout error and qubit π pulse errors). We estimate about 2% control errors, totalling to 9% error for the AQEC pulse.

Metric	X Gate						H Gate			
	Simulation			Experiment			Simulation		Experiment	
	**EsT**	**LE**	**Ord**	**EsT**	**LE**	**Ord**	**EsT**	**Ord**	**EsT**	**Ord**
**With all errors**										
Net fidelity	0.974	0.974	0.975	–	–	–	0.975	0.977	–	–
Code space fidelity	0.990	0.988	0.987	0.985	0.971	0.984	0.991	0.989	0.988	0.986
Error space fidelity	0.992	0.990	0.190	0.960	0.930	0.200	0.992	0.218	0.967	0.200
ET fidelity	0.676	0.476	0.430	0.750	0.400	0.350	0.669	0.347	0.750	0.350
Ideal QEC	0.983	0.980	0.978	–	–	–	0.985	0.981	–	–
**No qubit errors**										
Net fidelity	0.985	0.986	0.984	–	–	–	0.987	0.987	–	–
Code space fidelity	0.996	0.995	0.992	0.985	0.980	0.983	0.997	0.995	0.986	0.984
Error space fidelity	0.997	0.996	0.350	0.960	0.960	0.400	0.997	0.350	0.969	0.350
ET fidelity	0.843	0.638	0.533	0.850	0.500	0.500	0.898	0.381	0.880	0.400
Ideal QEC	0.994	0.991	0.986	–	–	–	0.996	0.989	–	–
**No oscillator errors**										
Net Fidelity	0.985	0.987	0.986	–	–	–	0.987	0.989	–	–

Supplementary Table 2. **Fidelity comparison of X and H gates in experiment and simulation:** The fidelity values noted for simulations are obtained from propagating the states with master equation solver for the gate Hamiltonian with all device parameters. It does not account for any state preparation or measurement loss. Values in the experiment sections are extracted from models that fit the experimental data.

**Gate**	**Error**	Net fidelity	Code space fidelity	Error space fidelity	ET fidelity	Ideal AQEC
**EsT**	No ancilla T_1	0.980	0.994	0.995	0.760	0.989
	No ancilla T_2	0.979	0.993	0.994	0.740	0.988
	No ancilla T_1 & T_2	0.985	0.996	0.997	0.843	0.994
**Ord**	No ancilla T_1	0.978	0.989	0.345	0.463	0.982
	No ancilla T_2	0.980	0.990	0.346	0.475	0.983
	No ancilla T_1 & T_2	0.984	0.992	0.350	0.533	0.986

Supplementary Table 3. **Error budget for ancilla T_1 and T_2 errors for EsT and Ord X gate**

The AQEC pulse has extra error compared to idling (2.7%) for the following reasons. During the AQEC pulse the oscillator states are entangled with the qubit and hence qubit errors cause error in the oscillator state. During the 1 µs pulse the oscillator has a slightly higher photon loss probability ($\bar{n} = 2.2$) compared to idling. If the qubit is not fully disentangled with the cavity at the end of the operation, the readout projection will dephase cavity states. Further, any readout assignment errors will wrongly map the ancilla state, and this error will propagate through the decode pulse. The ancilla reset π pulse has finite bandwidth. So, depending on the cavity state, the ancilla may end up having a small population in excited state, and the cavity will accumulate ancilla state-dependent phase. To achieve the maximum gain in fidelity, the AQEC pulse should only be applied when accumulated idling error (or gate errors) is larger than these extra errors the AQEC process introduces.

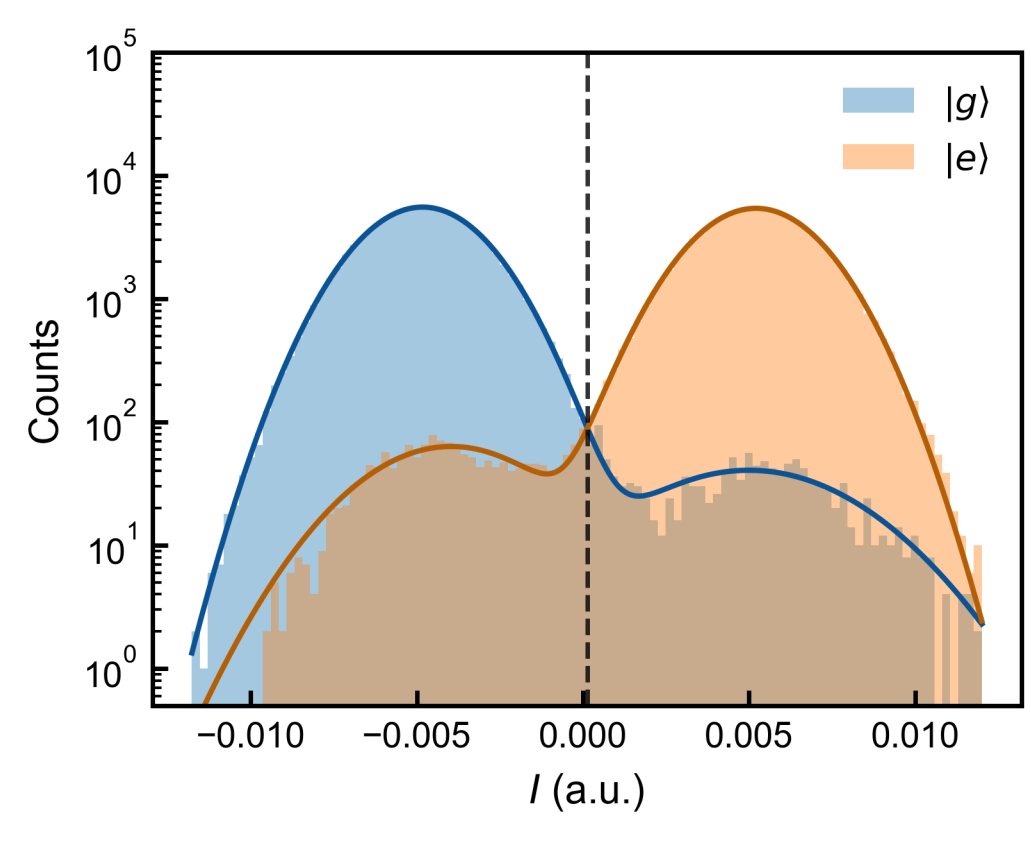

Supplementary Figure 8. **Readout fidelity:** Readout histogram showing the qubit prepared in ground and excited state and the threshold (vertical dashed line) used to discriminate between ground and excited states for single shot readout.

V. ADDITIONAL EXPERIMENTAL DATA

In this section, we present extended dataset that was used for main text figures and additional experimental data that supports our claims.

For measuring readout fidelity, we prepare the qubit in ground state (by waiting for $> 10T_{1q}$) and excited state (applying a $R_X(\pi)$ rotation) and readout the state. We choose a threshold for readout signal for single-shot determination of the readout outcomes that maximizes the average readout fidelity. We obtain a readout fidelity for $|g\rangle$ and $|e\rangle$ states to be 0.993 and 0.983 respectively. This gives us an average readout fidelity of 0.988. The single shot readout histogram is shown in Supplementary Fig. 8. After the AQEC pulse a readout is performed to project the ancilla state and subsequently a conditional π pulse is applied to the qubit to reset it to ground state. The condition of whether to play the pulse is evaluated directly on the FPGA based on whether the received signal from the readout resonator is greater than the pre-determined threshold for the qubit being in excited state. This condition evaluation has a latency of 300 ns. The AQEC pulse takes into account the oscillator Kerr related evolution during this latency delay and the preceding readout of the qubit that takes another 900 ns.

Similarly, we characterize the fidelity of our parity measurement sequence $R_X(\pi/2)$ − wait $1/2\chi - R_X(\pi/2)$ after preparing code and error space states. Note that, for our code and error space process fidelity experiments, we have performed separate experiments for extracting code space and error space process fidelities. So the second pulse in the parity measurement is a $R_X(\pi/2)$ pulse for detecting error space states and a $-R_X(\pi/2)$ pulse for detecting code space states. This maps the qubit to ground state at the end of pulse and the same decoding pulse can be used for all experiments. This also eliminates the asymmetry in parity measurement arising from the qubit being in ground or excited state during the readout. However, a small asymmetry still arises due to the finite bandwidth of the pulse and difference in spread of weights in Fock states between code and error subspaces. This leads to a parity measurement fidelity 94.4% and 95.2% for code and error space states. This error includes the error of the parity measurement, and the readout preceding the parity measurement. Thus the average error in the parity measurement process is 94.8%. For simplicity, we note this net error of $\sim 5\%$ as parity mis-assignment error (ϵ_{parity}) in the error model analysis for code and error space process fidelity decay curves in Supplementary Sec. VI.

For calculating the process fidelity shown in Fig. 3-5 in main text, we first perform process tomography [25, 26]. For this we prepare the qubit in four cardinal points of the Bloch sphere: $\{|g\rangle, |e\rangle, (|g\rangle+|e\rangle)/\sqrt(2), (|g\rangle+i|e\rangle)/\sqrt(2)\}$. Then the information is encoded onto the logical Bloch sphere with the encode operation and our process (gates, QEC, parity measurement as applicable in the respective experiment) is performed. Finally, the information is decoded back to the ancilla with decode operation and the ancilla state is measured in all three Pauli basis. From this we obtain the 4×4 process matrix χ_G. The fidelity is defined as the overlap of the process matrix with the target process matrix χ_{target} as $F = \text{tr}(\chi_G \chi_{\text{target}})$. The encode-decode process (which should be an identity operation) has a process fidelity of $\sim 91\%$.

Supplementary Fig. 9 shows additional experimental data for code and error space process fidelity for H and T gate. Experimentally measured data is plotted as circles (for code space) and triangle (for error space). Solid lines for error space and dashed lines for code space are models that fit the experimental data. Shaded regions represent 1 standard deviation of fidelities measured in repeated experiments. Supplementary Fig. 9a and Supplementary Fig. 9b respectively shows the experimental data and model without parity selection errors for H gate. These two plots confirm similar code space performance for EsT and Ord gate but significant improvement in performance for EsT gate in error space over the Ord gate. Supplementary Fig. 9c shows the experimentally measured probabilities of qubit being in ground state at the end of the gate operation (square points) and the probability that the state is an odd parity state (crosses) i.e. in error space. The state is found in odd parity state if a photon loss error happens during the operation. This plot shows error rates for qubit relaxation and oscillator photon loss are comparable for the H gate. However, the ET gate has slightly higher probability of being in the odd state at the end of the operation than the Ord gate. This can be attributed to higher average photon number for the ET gate during the operation.

Similarly, Supplementary Fig. 9d and Supplementary Fig. 9e respectively shows the experimental data and model without parity selection errors for T gate. They also confirm the similar performance in code space and different performance in error space for the T gate. Supplementary Fig. 9f shows that probabilities of qubit error and oscillator photon loss error are comparable for both ET and Ord T gate. We do not fully understand the reason for the small mismatch of error space model and experimental data for the Ord T gate.

We then turn to experimentally verifying the performance of LE gate described in Supplementary Supplementary Fig. 10. This gate is optimized for maximizing fidelity in code and error space but not for error transparency. The code and error space performance is similarly plotted in Supplementary Fig. 10a alongside EsT and Ord gate data. The data for EsT and Ord gate is the same as in Fig. 3b in main text, and is presented here for ease of performance comparison. Supplementary Fig. 10b shows the modeled gate fidelities without parity selection errors. This data confirms that LE gate performance is better than Ord gate as the operation is high fidelity in error space but is worse than EsT gate as it is not optimized for error transparency. Hence a photon loss during the operation still causes a significant loss in fidelity of the operation. It signifies the importance of the error trans-

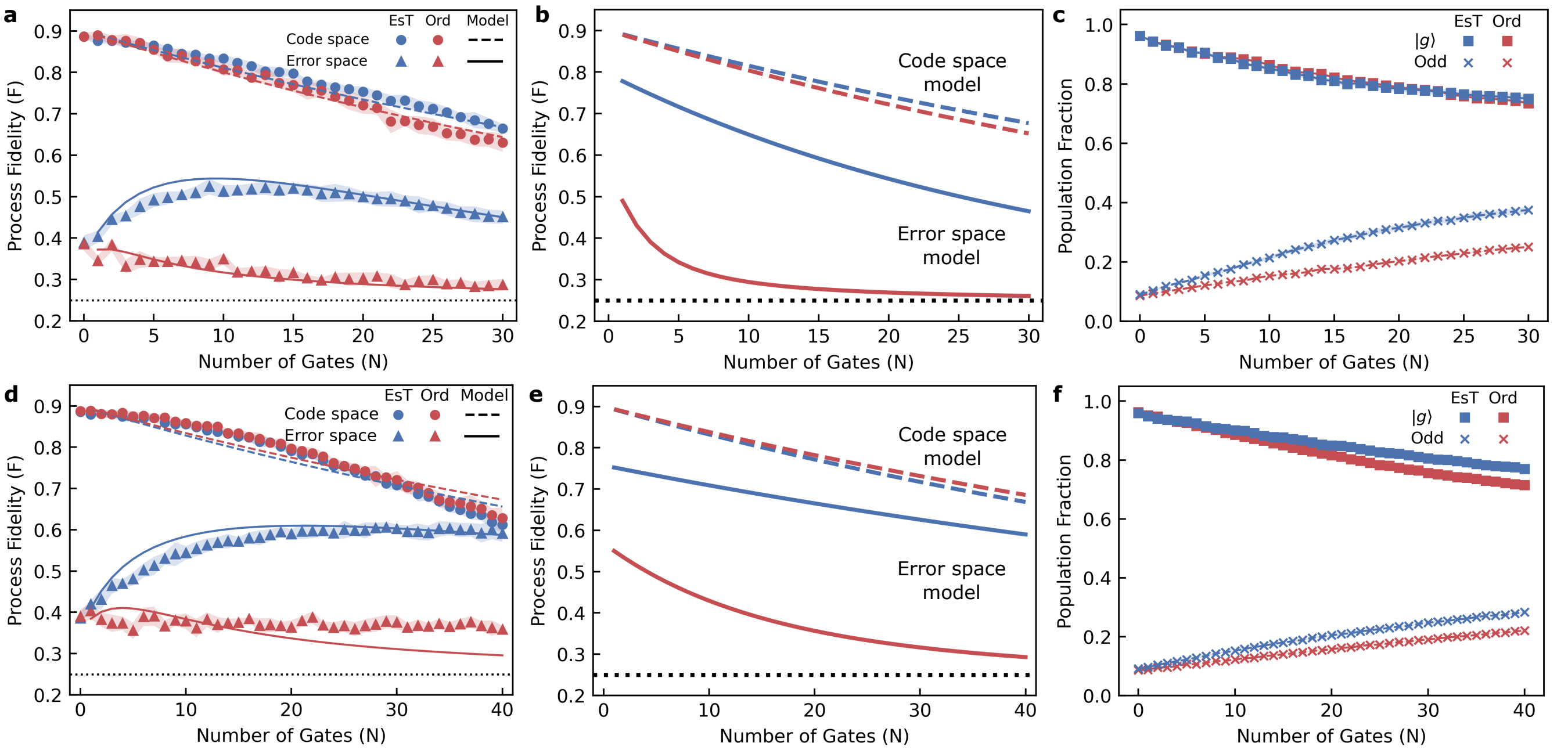

Supplementary Figure 9. **Code and error space process fidelity:** (a) Experimentally measured code and error space process fidelities with number of gates applied for H gate with modeled decay curves shown. (b) We plot the decay curves without parity selection error used for selecting code and error space states. (c) experimentally measured probability of finding qubit in ground state $|g\rangle$ and measuring a state in odd parity at the end of the gate operation for H gate. Similarly for T gates, (d) experimentally measured process fidelities and modeled decay curves, (e) decay curves without parity selection errors, and (f) experimentally measured probabilities finding qubit in ground state $|g\rangle$ and measuring a state in odd parity at the end of the gate operation are plotted. This data set and models has been used to extract the per-gate fidelity shown in Fig. 3(d) in main text.

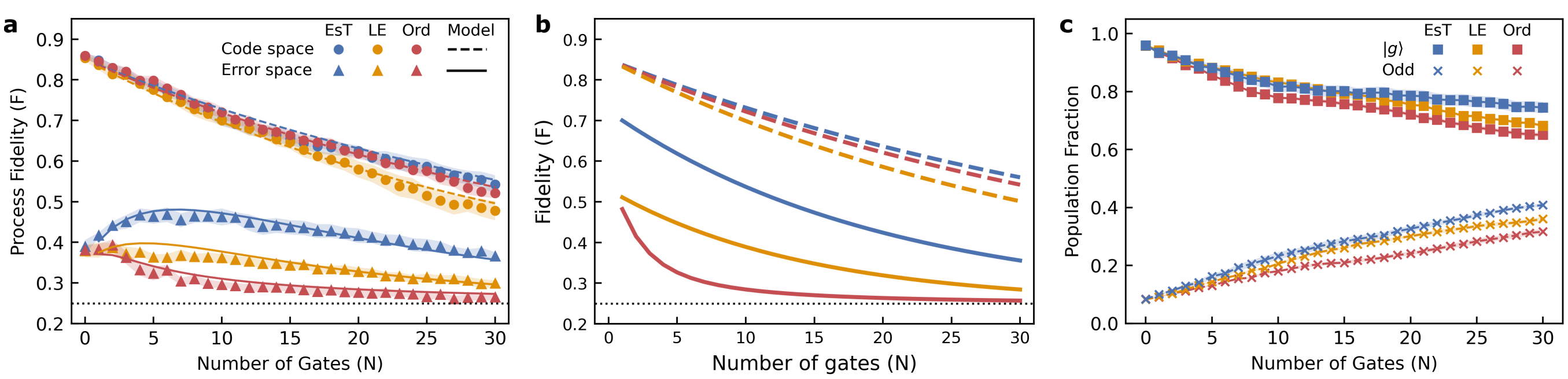

Supplementary Figure 10. **LE gate performance for X operation:** (a) Experimentally measured code and error space performance of LE gate, optimized for code and error space process fidelity but not ET, plotted alongside EsT and Ord gate experiments in Fig. 3(b) in main text. Modeled decay curves are shown as lines. The LE gate performance is similar to other gates in code space. In error space LE gate performs better than Ord gate as the fidelity of operation in error space is higher but it performs worse than EsT gates as it is not optimized for the ET condition. (b) Model decay curves without parity selection errors are plotted. (c) experimentally measured probabilities finding qubit in ground state $|g\rangle$ and measuring a state in odd parity at the end of the gate operation are plotted for all three gates.

parency cost defined in Eq. (15) for optimization. Similar to the previous figure, experimentally measured probabilities of qubit being in ground state and the probability of finding an odd parity state (an error space state) is shown in Supplementary Fig. 10c. The error rates due to qubit relaxation and oscillator photon loss are comparable and remain consistent for all three Ord, LE and EsT X gates.

The code and error space process fidelity data presented so far is post-selected on qubit being in ground state to avoid complications of qubit errors and those propagating into parity selection and decode operations. However, qubit errors are an integral part of these gate operations. So it is important to understand the gate dynamics when a qubit relaxation error happens during the gate. We can similarly perform code and error space process fidelity experiments unselective of qubit errors. Supplementary

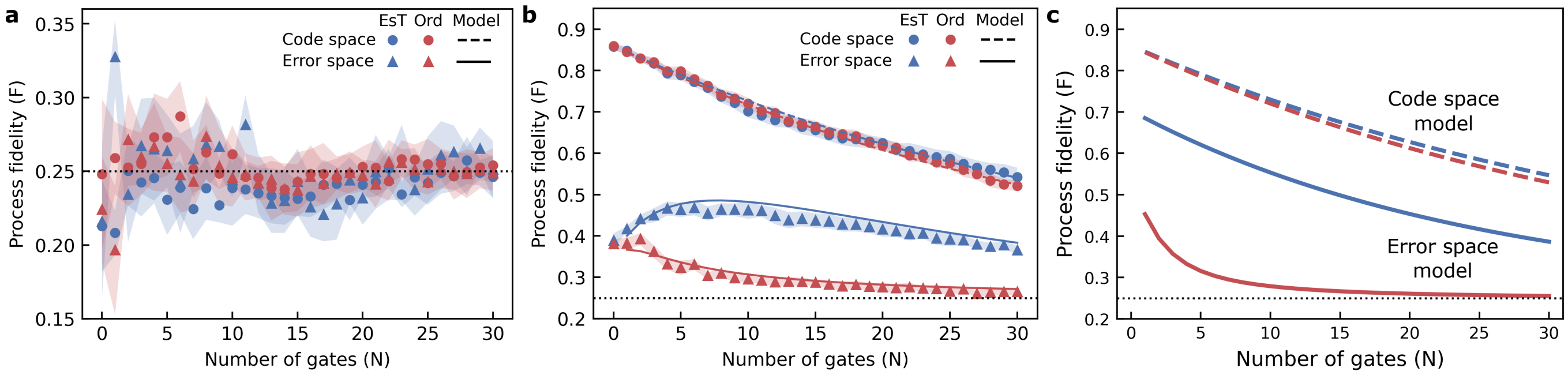

Supplementary Figure 11. **Code and error space process fidelity with qubit errors:** (a) code and error space process fidelities measured for X gate post-selected on the cases where qubit is found in the excited state $|e\rangle$ at the end of gate operations. It shows that a qubit error scrambles the quantum information completely. (b) We combine the cases of qubit in ground state or qubit in excited state after the gates to plot process fidelities unselective of qubit errors. The plot still shows a significant improvement of error space process fidelity for EsT gate compared to Ord gate although the absolute fidelity numbers are lower. (c) Modeled decay curve without parity selection error further validates the performance improvement of EsT gate.

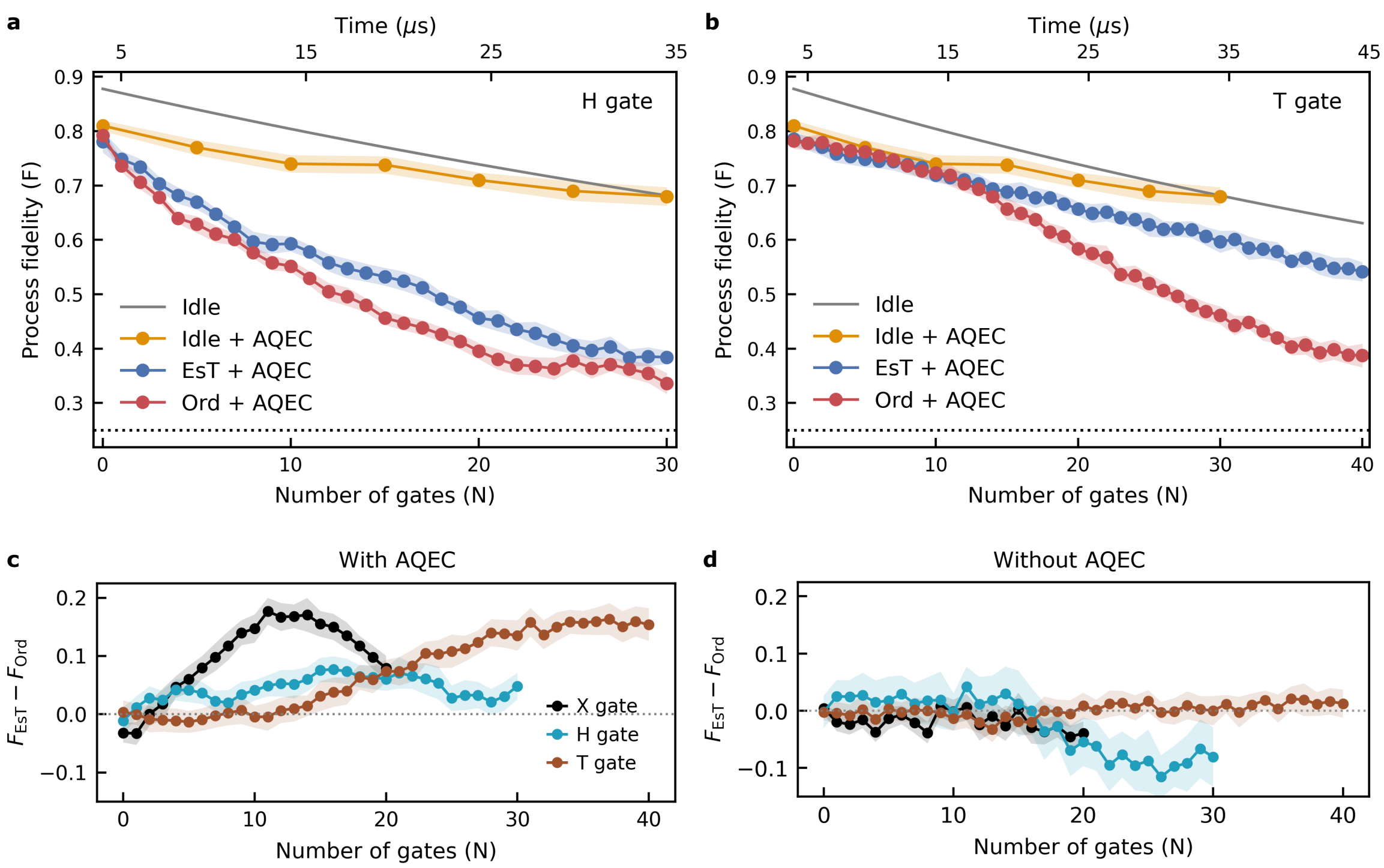

Supplementary Figure 12. **Gate performance improvement with AQEC:** Process fidelity after applying a single AQEC pulse after applying N gates for EsT and Ord (a) H gate and (b) T gate. Process fidelity decay for idling and for applying an AQEC pulse after idling for an equivalent duration of N gates. The horizontal dashed line at process fidelity of 0.25 indicates a completely incoherent process and shaded regions around the data points signify 1 standard deviation of fidelities for repeated measure-ments. (c) Performance improvement comparison of different gates as a function of number of gates applied before AQEC pulse. This is the same plot shown in Fig. 4(c) in main text. (d) Performance comparison of EsT and Ord gates with number of gates without AQEC. This shows that the EsT gates performed worse than Ord gate due to Hamiltonian parameter fluctuations. Nevertheless, after AQEC the EsT gate performance improves compared to Ord gates.

Fig. 11a shows the experimental data for EsT and Ord X gate performance for the cases when qubit is found in the excited state at the end of the operation. If these cases are considered, the absolute values of code and error space process fidelities will drop. The final fidelities for the EsT and Ord gate unselective of qubit errors is presented in Supplementary Fig. 11b, and the modeled decay curves without parity selection errors are shown in Supplementary Fig. 11c. This data shows that without selecting for qubit errors the absolute numbers for code and error space process fidelities are lower. However, the similarity in performance in code space and improvement

in performance in the error space for EsT gate remains true.

Finally we present additional data for AQEC sequence shown in Fig. 4a in main text for H and T gate respectively in Supplementary Fig. 12a and Supplementary Fig. 12b. This data was used to plot the performance improvement comparison of EsT and Ord gates for the three X, H and T operations shown in Fig. 4c in main text. The same plot is shown in Supplementary Fig. 12c for ease of comparison with Supplementary Fig. 12d which shows the performance difference of EsT and Ord gate without the AQEC pulse. As we can see the EsT gate performance is either worse or similar to the performance of Ord gate. This is primarily due to parameter fluctuations causing the EsT gate fidelity to be worse than Ord gate. However, despite worse performance of EsT gate without AQEC, the performance improvement with the AQEC pulse shows that EsT gate is preserving target state amplitude and phase coherence in the error space. In future, including robustness to parameter fluctuation as an optimization objective and doing hardware feedback based calibration can be used for removing such errors.

It is also worth noting that, while H gate shows the maximum performance improvement in error space fidelity for the EsT gate as shown in Fig. 3d in main text, it also has a higher probability of photon loss error during the operation as indicated by the probability of measuring odd parity state shown in Supplementary Fig. 9c. Our AQEC operation is also not very high fidelity. Hence the combination of higher fraction of states in the odd space, low AQEC fidelity and a low fidelity of the gate in absence of AQEC due to control errors causes the H gate performance with AQEC to not be significantly higher compared to Ord gate as presented in Fig. 4c in main text and Supplementary Fig. 12c.

VI. MODELING CODE AND ERROR SPACE FIDELITY

For the binomial 'kitten' code, code space states are even parity and error space states are odd parity. Therefore, measurements of Fock state parity, allows categorization of states as being potentially in the code or error spaces. This parity measurement has infidelity due to qubit relaxation or dephasing during the measurement, readout assignment error, and finite pulse bandwidth of the qubit $\pi/2$ pulses used for parity mapping and control errors. Besides this, due to highly asymmetric population in even and odd states for the binomial code, the sparse error space population means that the measurement infidelity contributes a particularly high proportion of the error in parity selection. We illustrate and analyze this issue in this subsection.

Before the parity measurement, probability that the state in the cavity is odd is written as $P(O)$. This depends on the intrinsic error rate of the oscillator, duration of the gate operation, mean photon number in the oscillator and importantly the number of gates applied. Parity mis-assignment error is termed as ϵ_{parity} which signifies the probability that parity selection maps to wrong parity state. This is assumed to be symmetric for odd and even states. For the odd parity state, we can write the probability that a truly odd state is measured to be odd (M_O) as

$$P(M_O|O) = 1 - \epsilon_{\text{parity}} \tag{23}$$

So the probability that a state measured in odd is actually odd is given by

$$P(O|M_O) = P(M_O|O)P(O)/P(M_O) \ , \tag{24}$$

where, $P(M_O) = P(M_O|O)P(O) + P(M_O|E)P(E) = P(M_O|O)P(O) + (1 - P(M_O|O))P(E)$. Here, $P(E) = 1 - P(O)$ is the probability of the state being even before the parity measurement and $P(M_O|E) = 1 - P(M_O|O)$ is the probability that an even parity state is measured to be odd parity. For the 'true' odd states passing through the selection, we label with fidelity F_{true}. For the even states selected as odd, we label with fidelity F_{alias}. So the measured fidelity will be,

$$F_{\text{measured}} = P(O|M_O)F_{\text{true}} + (1 - P(O|M_O))F_{\text{alias}} \ . \tag{25}$$

The decode operation used in our experiment is designed to map odd states from the error space Bloch sphere to the qubit Bloch sphere. So when this decode operation is applied on the even states no relevant quantum information is mapped to the qubit Bloch sphere. So F_{alias} is taken to be 0.25 in our model.

In the F_{true}, we also have two components. A fraction of odd states are generated due to photon loss during the gate operation. We define this probability as $P(\text{O}, \text{gate})$ and the corresponding fidelity as $F_{\text{O,gate}}$. The other fraction of odd states were odd because of states that were odd at the start of the gate because of error during the encode process. This is essentially a state preparation error. We define this probability as $P(\text{O}, \text{prep})$ and fidelity as $F_{\text{O,prep}}$. The readout error and loss during the parity measurement can be thought of as a parity mapping error and can be absorbed into ϵ_{parity}. So, we can write

$$F_{\text{true}} = P(\text{O}, \text{gate})F_{\text{O,gate}} + P(\text{O}, \text{prep})F_{\text{O,prep}} \ . \tag{26}$$

We are interested in the quantity $F_{\text{O,gate}}$. Here, $P_{\text{O,gate}}$ and $P(\text{O}, \text{prep})$ are normalized such that they add up to 1. If not normalized they should add up to $P(O)$.

A similar analysis can be done for the even states. However, it is worth noting that in our case of binomial 'kitten' code, when applying logical operations starting from code space (even parity states), we will most often end in even parity states. Only when a single photon loss error happens during the gate operation, we will end up in error space (odd parity states). Hence, this parity selection error massively affects the fidelity of states measured in the error space whereas fidelity of the states measured in code space is much closer to the true even parity population.

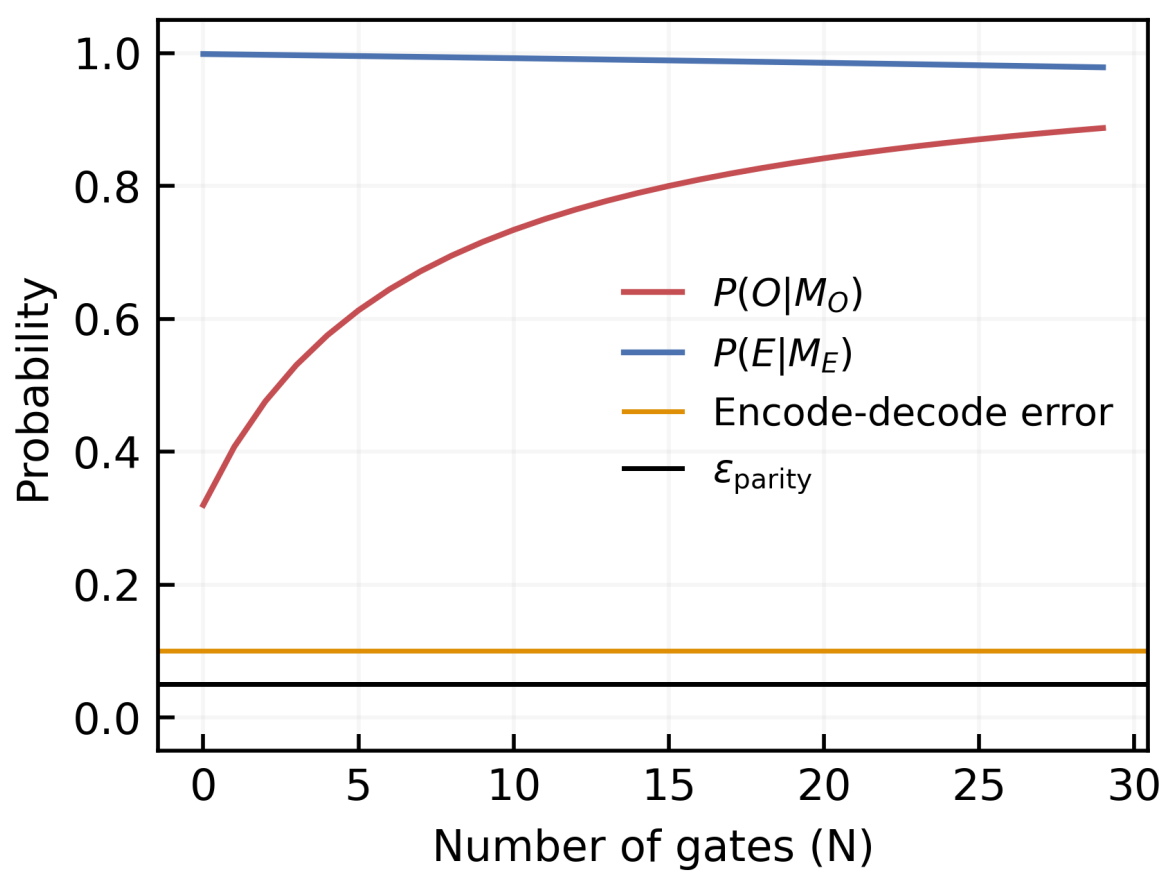

Supplementary Figure 13. **Error model for parity selection errors:** We plot the error probability of errors in parity measurement (ϵ_{parity}) and encode-decode process. We also plot the probability that a state is truly odd if it is measured odd ($P(O|M_O)$) and that a state is truly even if it is measured even ($P(E|M_E)$). This summarizes the contribution of various error channels in code and error space process fidelity measurement.

We show the probabilities of different errors in Supplementary Fig. 13. ϵ_{parity} is fixed at 0.05 (black horizontal line) for our experiment which is fixed for any number of gates applied. The error in the encode-decode process for preparing a logical state and reading it is about 9% is also shown as orange horizontal line. The plot shows $P(O|M_O)$ and $P(E|M_E)$ with number of applied gates. The numbers used in this model follow the device parameters for our experiment. The $P(O|M_O)$ as a function of number of gates show that the probability that a state measured odd is truly odd increases with number of gates as more number of gates mean longer duration of operation and hence more photon loss errors in the oscillator. And the large change in this probability is the reason for large difference in decay profile, between the experimentally measured fidelity and the parity-selection error free fidelities as seen in Fig. 3 in main text, Supplementary Fig. 9, 10, 11.

Next, we model the true code and error space process fidelities. For the code space, we model with an exponential fidelity decay due to coherence times and control errors. These can be combined to write the fidelity with number of gates as

$$F_{\text{code}}(N) = A \exp\left(-\gamma_C N\right) + B \ , \tag{27}$$

where, A and B are constants and γ_C is the error rate per gate in the code space. We set $B = 0.25$ as the point where the process is completely incoherent and choose A accordingly to match the process fidelity of encode-decode process measured in experiments. γ_C depends on the gate duration, coherence times of the cavity and qubit and control errors.

For the error space, we need a more complicated model as the fidelity measured at N-th gate includes photon loss at the k-th step for $k \leq N$ and error space gate fidelity for the $(N-k)$ gates. The photon loss is equally likely during each gate operation. For the N-th gate process fidelity measured in error space, the photon loss happened in between $k = 1$ to N gates with probability of loss $1/N$ per gate. if the photon loss happened at k-th step, then fidelity due to this branch will be

$$f_k = F_C^{k-1} F_{\text{err|jump}} F_E^{N-k} \ , \tag{28}$$

where, $F_C = e^{-\gamma_C}$ is the fidelity per gate in code space and $F_E = e^{-\gamma_E}$ is the fidelity per gate in error space and $F_{\text{err|jump}}$ is the fidelity when there is a jump from code to error space during the gate operation. γ_E is the error rate in error subspace which depends on the gate duration, coherence times of the cavity and qubit and control errors for the error space operation. $F_{\text{err|jump}}$ is related to the error transparency of our pulse: the more error transparent the pulse is, the higher will be $F_{\text{err|jump}}$. So the total fidelity in the error space for the N-th gate should be, $F_{\text{error}}(N) = D.\frac{1}{N}\sum_{k=1}^{N} f_k$. D is the scaling that includes encode decode process fidelity. This is a geometric series which can be summed to

$$F_{\text{error}}(N) = \frac{D F_{\text{err|jump}}}{N} \frac{F_C^N - F_E^N}{F_C - F_E} + G \ , \tag{29}$$

where, the constant $G = 0.25$ is the lower limit of fidelity and signifies a completely incoherent process. D is then chosen to match the encode-decode process fidelity measured in experiment in the error subspace. We choose $D = 0.6$ for our model.

This model works well for the Ord gate where F_C and F_E are quite different. But for ET gate, $F_C \approx F_E$ and we need to take the limit of this Eq. (29). Taking the limit $F_C - F_E \to 0$, for the ET gate, we have

$$F_{\text{error,ET}}(N) = D \cdot F_{\text{err|jump}} \cdot F_C^{N-1} + G \ . \tag{30}$$

We assume the same single photon loss rate for the driven system (during the gate) as while idling in this model. We have also not considered double photon loss errors but they remain smaller than 4% at the 30 µs timescale we have considered in our experiments. So our first order model fits the experimental data reasonably well (Fig. 3 in main text, Supplementary Fig. 9, 10, 11) and allows us to extract relevant error rates and fidelities.

[1] T. Tsunoda, J. D. Teoh, W. D. Kalfus, S. J. de Graaf, B. J. Chapman, J. C. Curtis, N. Thakur, S. M. Girvin, and R. J. Schoelkopf, Error-detectable bosonic entangling gates with a noisy ancilla, PRX Quantum **4**, 020354 (2023).

[2] O. C. Wetherbee, S. Roy, B. Royer, and V. Fatemi, A Mathematical Structure for Amplitude-Mixing Error-Transparent Gates for Binomial Codes, Quantum **9**, 1890 (2025).

[3] V. V. Albert, K. Noh, K. Duivenvoorden, D. J. Young, R. T. Brierley, P. Reinhold, C. Vuillot, L. Li, C. Shen, S. M. Girvin, B. M. Terhal, and L. Jiang, Performance and structure of single-mode bosonic codes, Phys. Rev. A **97**, 032346 (2018).

[4] S. Roy, A. Senanian, C. S. Wang, O. C. Wetherbee, L. Zhang, B. Cole, C. P. Larson, E. Yelton, K. Arora, P. L. McMahon, B. L. T. Plourde, B. Royer, and V. Fatemi, Synthetic high angular momentum spin dynamics in a microwave oscillator, Phys. Rev. X **15**, 021009 (2025).

[5] M. Reagor, W. Pfaff, C. Axline, R. W. Heeres, N. Ofek, K. Sliwa, E. Holland, C. Wang, J. Blumoff, K. Chou, M. J. Hatridge, L. Frunzio, M. H. Devoret, L. Jiang, and R. J. Schoelkopf, Quantum memory with millisecond coherence in circuit qed, Phys. Rev. B **94**, 014506 (2016).

[6] G. J. Dolan, Offset masks for lift-off photoprocessing, Applied Physics Letters **31**, 337 (1977).

[7] M. J. Persky, Review of black surfaces for space-borne infrared systems, Review of Scientific Instruments **70**, 2193 (1999), https://pubs.aip.org/aip/rsi/article-pdf/70/5/2193/19091542/2193_1_online.pdf.

[8] P. Guilmin, A. Bocquet, É. Genois, D. Weiss, and R. Gautier, Dynamiqs: an open-source python library for gpu-accelerated and differentiable simulation of quantum systems (2025).

[9] N. Khaneja, T. Reiss, C. Kehlet, T. Schulte-Herbrüggen, and S. J. Glaser, Optimal control of coupled spin dynamics: design of nmr pulse sequences by gradient ascent algorithms, Journal of Magnetic Resonance **172**, 296 (2005).

[10] J. Bradbury, R. Frostig, P. Hawkins, M. J. Johnson, C. Leary, D. Maclaurin, G. Necula, A. Paszke, J. VanderPlas, S. Wanderman-Milne, and Q. Zhang, JAX: composable transformations of Python+NumPy programs (2018).

[11] D. P. Kingma and J. Ba, Adam: A method for stochastic optimization, in *3rd International Conference on Learning Representations, ICLR 2015, San Diego, CA, USA, May 7-9, 2015, Conference Track Proceedings*, edited by Y. Bengio and Y. LeCun (2015).

[12] DeepMind, I. Babuschkin, K. Baumli, A. Bell, S. Bhupatiraju, J. Bruce, P. Buchlovsky, D. Budden, T. Cai, A. Clark, I. Danihelka, A. Dedieu, C. Fantacci, J. Godwin, C. Jones, R. Hemsley, T. Hennigan, M. Hessel, S. Hou, S. Kapturowski, T. Keck, I. Kemaev, M. King, M. Kunesch, L. Martens, H. Merzic, V. Mikulik, T. Norman, G. Papamakarios, J. Quan, R. Ring, F. Ruiz, A. Sanchez, L. Sartran, R. Schneider, E. Sezener, S. Spencer, S. Srinivasan, M. Stanojević, W. Stokowiec, L. Wang, G. Zhou, and F. Viola, The DeepMind JAX Ecosystem (2020).

[13] P. Reinhold, S. Rosenblum, W.-L. Ma, L. Frunzio, L. Jiang, and R. J. Schoelkopf, Error-corrected gates on an encoded qubit, Nature Physics **16**, 822 (2020).

[14] Q. Xu, P. Zeng, D. Xu, and L. Jiang, Fault-tolerant operation of bosonic qubits with discrete-variable ancillae, Phys. Rev. X **14**, 031016 (2024).

[15] Y. Ma, Y. Xu, X. Mu, W. Cai, L. Hu, W. Wang, X. Pan, H. Wang, Y. P. Song, C.-L. Zou, and L. Sun, Error-transparent operations on a logical qubit protected by quantum error correction, Nature Physics **16**, 827 (2020).

[16] D. T. McClure, H. Paik, L. S. Bishop, M. Steffen, J. M. Chow, and J. M. Gambetta, Rapid driven reset of a qubit readout resonator, Phys. Rev. Appl. **5**, 011001 (2016).

[17] V. V. Sivak, A. Eickbusch, H. Liu, B. Royer, I. Tsioutsios, and M. H. Devoret, Model-free quantum control with reinforcement learning, Phys. Rev. X **12**, 011059 (2022).

[18] Y. Baum, M. Amico, S. Howell, M. Hush, M. Liuzzi, P. Mundada, T. Merkh, A. R. Carvalho, and M. J. Biercuk, Experimental deep reinforcement learning for error-robust gate-set design on a superconducting quantum computer, PRX Quantum **2**, 040324 (2021).

[19] J. M. Gertler, B. Baker, J. Li, S. Shirol, J. Koch, and C. Wang, Protecting a bosonic qubit with autonomous quantum error correction, Nature **590**, 243 (2021).

[20] S. Shirol, S. van Geldern, H. Xi, and C. Wang, Passive quantum error correction of photon loss at breakeven (2025), arXiv:2510.19794.

[21] Z. Ni, L. Hu, Y. Cai, L. Zhang, J. Mai, X. Deng, P. Zheng, S. Liu, S.-B. Zheng, Y. Xu, and D. Yu, Autonomous quantum error correction beyond break-even and its metrological application (2025), arXiv:2509.26042.

[22] L. Sun, Y. Xu, Y. Zhou, Z. Hua, W. Wang, J. Zhou, Z. jie Chen, L. Z. de Paula, Q.-X. Jie, G. Xue, H. Yu, W. Cai, C.-L. Zou, and L. Sun, Extending coherence time beyond break-even point using only drives and dissipation (2025), arXiv:2509.22191.

[23] T. Propson, B. E. Jackson, J. Koch, Z. Manchester, and D. I. Schuster, Robust quantum optimal control with trajectory optimization, Phys. Rev. Appl. **17**, 014036 (2022).

[24] P. M. Poggi, G. De Chiara, S. Campbell, and A. Kiely, Universally robust quantum control, Phys. Rev. Lett. **132**, 193801 (2024).

[25] M. A. Nielsen and I. L. Chuang, *Quantum Computation and Quantum Information: 10th Anniversary Edition* (Cambridge University Press, Cambridge, 2010).

[26] A. Hashim, L. B. Nguyen, N. Goss, B. Marinelli, R. K. Naik, T. Chistolini, J. Hines, J. Marceaux, Y. Kim, P. Gokhale, T. Tomesh, S. Chen, L. Jiang, S. Ferracin, K. Rudinger, T. Proctor, K. C. Young, I. Siddiqi, and R. Blume-Kohout, Practical introduction to benchmarking and characterization of quantum computers, PRX Quantum **6**, 030202 (2025).
