# Supplementary material for: Error semitransparent universal control of a bosonic logical qubit
Source: Nat Commun. 2026 Oct 8;17:11441. doi: 10.1038/s41467-026-78317-y (PMC13737002; doi:10.1038/s41467-026-78317-y)
Supplement: Supplementary file 2 [file 41467_2026_78317_MOESM2_ESM.pdf]

nature portfolio

Peer Review File

Error Semitransparent Universal Control of a Bosonic Logical Qubit

Corresponding Author: Professor Valla Fatemi

This file contains all reviewer reports in order by version, followed by all author rebuttals in order by version.

Version 0:

Reviewer comments:

Reviewer #1

(Remarks to the Author)
In this paper, the authors demonstrated error semi-transparent (EsT) operations on a bosonic logical qubit to implement universal logical gate set: X, H and T. Previous demonstrations of ET gates were limited to logical phase rotations and implementing universal gate set that are transparent to photon loss errors was an open question. Despite the gates in this work not being completely transparent to photon loss (or being fault-tolerant to ancilla errors), they are still a very important result in the field, showing progress towards realizing fault-tolerant logical gates that are necessary to build quantum computers based on bosonic qubits.
The authors have implemented the EsT gates on a binomial kitten code and benchmarked their fidelity using three metrics that are stated in the theory. They observed a five-fold reduction in gate errors after photon jump compared to when using only ordinary gates. The key innovation is a framework based on dynamic encoding subspaces. Rather than requiring the code space and error space to remain fixed during the gate (the usual error-transparent approach), they allow these subspaces to evolve dynamically. This makes it possible to realize near-error-transparent gates using only standard linear control drives. I would recommend publishing this work in Nature Communications as it is an important experimental paper in the field that will be read by many people who work with bosonic qubits for quantum computing and move beyond using them only as a logical memory.

Some of the following questions to improve the manuscript:
1. The demonstrated EsT gates are implemented in the absence of continuous correction. Can the authors comment on whether the dynamic encoding framework can be combined with autonomous error-correction protocols operating simultaneously with the gate?
2. How does increasing the code size affect the optimization? Can I extend the gates to other codes like cat and GKP easily?
3. Fig.4a shows that the idle evolution with AQEC exhibits lower fidelity than the idle evolution without AQEC. Can the authors provide a quantitative breakdown of the dominant error mechanisms introduced by the AQEC protocol and explain why these outweigh the benefits of photon-loss correction in the idle experiment?
4. The recovery of process fidelities to 0.68 and 0.63 after AQEC. Could the authors provide a clear error budget table identifying the dominant sources of the remaining infidelity? This would help understand whether the current limitation arises from gate errors, decoherence, leakage, or the AQEC protocol itself.
5. Could the authors clarify how ancilla T1 and T2 specifically contribute to the EsT gate infidelity? In particular, it is not obvious how ancilla dephasing affects the gate operation.
6. In the Outlook section, the authors suggest using the ancilla g–f transition instead of the g–e transition, which could convert ancilla relaxation errors into detectable erasure errors. This is an interesting idea. Could the authors provide a rough quantitative estimate of the expected improvement in gate fidelity from this approach? In particular,how much of the current infidelity is attributed to ancilla relaxation, and what level of performance enhancement could realistically be achieved by using the g–f transition?
7. Could the authors discuss the contributions of thermal population and readout errors to the reported infidelities of the X, H, and T gates?
8. The authors suggest that the observed two-frequency behavior of the qubit g-e transition frequency is caused by the presence of some TLS. Could the authors comment on whether the qubit lifetime is also affected or limited by coupling to these TLS?

Reviewer #2

(Remarks to the Author)
In their manuscript, Roy, Wetherbee, and Fatemi address an important problem in fault-tolerant computation based on bosonic codes: the realization of fault-tolerant single-qubit gate set. The authors approach the problem for a binomial code (kitten code) for which they extend the concept of error transparent (ET) gates to a variety of single-qubit gates for which the established theory does not apply. Specifically, ET gates are found using numerical optimization constrained on the errors to commute at all times with the time-dependent unitary realizing the gate. The authors' \textit{error semi-transparency} (EsT) extends/changes the constraints to minimize (1) QEC violation (i.e. Knill-Laflamme conditions), (2) leakage outside of code's error subspaces, (3) difference in the coherent evolution between the logical and the error subspaces. This is the main contribution of the work. The authors then proceed to experimentally demonstrate the performance of this method, showing an improvement in the gate infidelity by a factor of 3-6 compared to the ET scheme in case of a photon loss event and that an improvement in the process fidelity when these gates are followed by one round of error correction.

I find that the manuscript makes valuable theoretical contributions to the study of fault tolerance of bosonic codes. Before recommending for the manuscript's publication in Nature Communication, I would appreciate the authors' answers to the following questions and, if relevant, modifications in the manuscript.

(a) The authors developed the EsT scheme for gates that fall outside the standard error-transparency formalism which only encompasses phase gates. Though, the T gate is a phase gate and must then be realizable using the ET scheme. I suspect that this explains its better performance compared to the H and X gates which must mix amplitudes. If the authors agree with this statements, I would suggest emphasizing this aspect which shows that EsT scheme is superior to the ET one even for phase gates.

Along the same line, I would appreciate to see the ``EsT, LE and Ord gate comparison'' (i.e. Fig. A3) for both H and T gates. Moreover for the latter, it would be valuable to have a direct comparison between ET and EsT schemes where the former would be realized using previously demonstrated approaches [7,8]. It would also be interesting to see the performance of ET and EsT schemes for the Z gate which is another instance of phase gates.

(b) On page 6 (line 316), the authors mention that they model the fidelity as a function of the number of gates. Even though it is explained in the SM, I encourage the authors to add a short explanation of how the model is derived in the main text. Something like ``The model assumes that a single photon loss event is equally likely to occur during any gate, and heuristically accounts for the encode-decode fidelity of 60\%'' would be sufficient.

(c) Given that the EsT scheme includes additional constraints compared to the Ord and ET schemes, is the optimization task always convergent, or does it get stuck in local minimas? How robust are the obtained pulses to parameter fluctuations?

(d) How do the code states look like throughout the optimized pulse sequence? How does their structure evolve? Do they become more vulnerable to other errors during this time? In other words, would the logical information be lost in case of some (probably unlikely) error events during the pulse sequence and what would these errors be (e.g., transmon dephasing, two or three photon losses)?

(e) The authors showed the gate and process fidelities assuming the system is either in the logical space or in the single-photon error subspace. Although these are relevant metrics for the main message of the work, what will ultimately matter is the averaged performance of the gates. It would therefore be interesting to determine the average gate fidelity for both Ord and EsT schemes and compare their scaling with respect to error rates (and eventually compare them against the ET results from Ref. [8]). How does the performance of the gates vary for different photon-loss and dephasing rates in these cases? Adding such a numerical comparison in the Supplemental Material would be helpful.

(f) Figure 4 benchmark the gates when these are repeated N times and followed by one round of error correction. It would be more insightful to see the performance of repeated application of gate + AQEC. Although Fig. A6(d) presents a simulation of such a benchmark, what did limit you from performing it in experiment?

Along the same line, I suspect the pulse sequence to be imperfect such that some population will get stuck in higher excited Fock states. Could you measure (or estimate) the amount of population outside of the $\{\ket{0_L},\ket{1_L}\}$ and $\{\ket{0_E},\ket{1_E}\}$ subspaces? Would one require an operation, similar to the leakage reduction units, that brings the leaked population back into the code space?

(g) Regarding broader use cases of the EsT scheme, could (and how would) it be applied to multi-qubit qubit gates?

Reviewer #3

(Remarks to the Author)
In the manuscript by Saswata Roy et al., the authors propose a method for error semi-transparent (EsT) operations. By incorporating instantaneous error-transparent (ET) conditions into the numerical optimization procedure, they show that approximately fault-tolerant logical operations on a bosonic logical qubit can be implemented using only linear drives on the bosonic mode and the ancillary qubit. The experimental results demonstrate that, after AQEC, logical operations

implemented using the EsT method achieve higher fidelities than non-fault-tolerant logical operations implemented by conventional methods followed by AQEC. This clearly supports the feasibility of the proposed approach. Moreover, the method only requires the addition of an extra cost function during the numerical optimization of the drive pulses, without requiring additional hardware overhead or extra external drives to engineer an effective Hamiltonian.

This work is clearly important and innovative. Its significance includes, but is not limited to, the following points.
First, to the best of my knowledge, this is the first experimental realization of fault-tolerant amplitude-mixing logical gates on a bosonic logical qubit. In particular, the manuscript demonstrates fault-tolerant logical X and H gates. Previous experiments had realized fault-tolerant logical phase gates, whereas this work extends the experimental capability toward a fault-tolerant universal gate set.
Second, the work addresses a long-standing difficulty in realizing fault-tolerant logical operations through numerical optimization. Numerical optimization is widely used across different physical platforms because it can implement target unitaries with shorter pulse durations. In superconducting bosonic systems, numerically optimized target unitaries can be faster than conventional SNAP + displacement constructions and can therefore yield higher fidelities. However, because the system dynamics during numerically optimized pulses are highly complex, it has been difficult to ensure fault-tolerant properties in such operations. This work overcomes this challenge by introducing instantaneous ET conditions and incorporating the corresponding cost function into the numerical optimization, thereby achieving logical operations that are both fast and approximately fault-tolerant. In addition, the experimental results shown in the manuscript likely do not represent the ultimate limit of this method. In the future, the performance could potentially be further improved by incorporating nonlinear operations, such as multi-photon drives.

For the reasons above, I support publication of this work in Nature Communications.

Below are several questions and comments that I would like the authors to address.
1. Could the authors provide numerical simulation results showing whether a strict ET gate against single-photon-loss error a can be achieved if multi-photon drives on the cavity are included? Under what conditions would this be possible? Alternatively, can one obtain logical operations with better performance than those demonstrated in the present work? In this question, I am not considering the additional noise that may be introduced by the nonlinear elements required to implement such multi-photon drives.
2. In Eq. (1), can the no-jump evolution be incorporated into the definition, so that a more rigorous instantaneous code space is defined?
3. In Appendix A, the authors mention that if the commutator between the Hamiltonian and the noise operator a is correctable, then the error-closure condition [10] is satisfied. The commutator between displacement and a, or equivalently the transformation of a under displacement, gives a linear combination of a and I, which should be correctable. Does this mean that the reason exact ET operations cannot be realized in the current implementation is mainly the presence of the self-Kerr term and higher-order cross-Kerr or dispersive terms? If so, the statement in the Discussion and Outlook that the linear drive and photon loss do not commute may not be the fundamental reason why errors occurring during the logical gate become uncorrectable. Based on this point, could the authors provide simulation results showing whether linear drives alone can achieve good ET behavior against photon-loss errors in the absence of self-Kerr and higher-order cross-Kerr or dispersive terms? Equivalently, what logical gate fidelity can be achieved in simulations if self-Kerr and higher-order dispersive shifts are neglected?
4. According to Appendix A, “error-closure [10]” is a generalization of error transparency. Should the definition of instantaneous leakage in Eq. (7) of the main text be modified to accommodate this more general ET condition? In my understanding, leakage should correspond to the population outside the sum of all correctable subspaces. For example, for photon loss a and displacement, the transformed error (commutator) contains both a and I components. Therefore, in addition to the target error manifold, part of the state can appear in the code space, which is still correctable. As a result, even when the commutator between the error and the Hamiltonian is correctable and satisfies the generalized ET condition, the quantity defined in Eq. (7) may not vanish.
5. After the AQEC operation, the entropy in the ancilla is removed through measurement-based conditional reset. However, the manuscript does not explain how this is implemented in hardware, for example on the FPGA. Could the authors provide more information on the feedback latency, the single-shot readout histograms of the ancilla, and how the threshold is chosen to discriminate between the (|g⟩) and (|e⟩) states?
6. In Appendix C, lines 317–320, the authors state: “Further using multi-level ancilla, ancilla ET can be similarly defined and included in the optimization directly. These will be explored in future works.” I am curious how ET for the dominant ancilla noise channels can be realized in this situation. For a single transmon qubit, even when higher levels are included, there is no quantum error-correcting code satisfying the Knill–Laflamme conditions. Moreover, for a strongly nonlinear transmon, damping processes between different levels, such as (|f⟩ \to |e⟩) and (|e⟩ \to |g⟩), as well as dephasing processes, are generally regarded as independent noise channels. This means that introducing higher levels also requires consideration of additional independent noise processes. Could the authors clarify how ancilla ET would be defined and implemented in this setting?
7. In Appendix A, lines 21–24, the authors write: “Consequently, a photon loss event during evolution under these terms of the Hamiltonian maps the final logical state |ψ_L⟩ to the corresponding error space state a|ψ_L⟩, up to a deterministic phase.” I may be misunderstanding this point, but it seems that this is not simply “up to a deterministic phase,” because according to the ET formula around line 85 of the main text, the phase can depend on the jump time (t). Is the reason that this time-dependent phase is a global phase within the error space and therefore does not affect the final result? The authors also mention around line 88 of the main text that it is a global phase. It would be helpful if this point could be clarified.
8. Can the AQEC pulse and the encode pulse, which are also implemented using numerical optimization, be modified to include the EsT condition? Why was EsT not incorporated for these pulses in the present work? If it cannot be incorporated, what are the potential obstacles? If EsT could be included, then in a repetitive AQEC sequence, later AQEC cycles could in

principle correct photon-loss errors that occur during earlier AQEC operations.
9. Why is the encode-decode fidelity only 91%? Could the authors provide more discussion of the dominant error sources limiting this fidelity?
10. Could the authors provide independent experimental characterization data for the parity measurement fidelity? The fidelities for even- and odd-parity measurements are not necessarily identical. For simplicity, in the model analysis in Appendix F, it may be acceptable to use a single value, such as the average of the experimentally characterized even- and odd-parity measurement fidelities, but the independent characterization data should be provided.

Minor comments:
1. In Eq. (1), the word “span” should be added.
2. In Eq. (7), ρ(t) should carry a subscript, i.e., ρ_j (t), corresponding to the error space (E_j).
3. In Appendix C, the index labeling the six different logical initial states, the index labeling different time steps, and the index labeling different cost functions in Eq. (C8) should be denoted by different letters to avoid confusion.
4. In the Discussion and Outlook section, lines 509–517, the authors mention the possibility of using the (|f⟩) state of the ancilla and employing path-independent or error-detectable methods to handle ancilla decoherence. I am curious whether the authors have considered the following issue. Suppose that in the future the method proposed in this work is extended to higher-order bosonic QEC codes, enabling logical gates and correction operations that can tolerate higher-order oscillator errors. After path-independent or error-detectable methods are used to handle first-order ancilla noise, how would second-order ancilla noise be treated, for example a sequence of damping events (|f⟩ to |e⟩) followed by (|e⟩ to |g⟩)? Would an even higher-dimensional ancilla be required?

Reviewer #4

(Remarks to the Author)
I co-reviewed this manuscript with one of the reviewers who provided the listed reports. This is part of the Nature Communications initiative to facilitate training in peer review and to provide appropriate recognition for Early Career Researchers who co-review manuscripts.

Version 1:

Reviewer comments:

Reviewer #1

(Remarks to the Author)
The authors have addressed my questions and revised the manuscript accordingly. I am pleased to recommend the revised manuscript for publication in Nature Communications.

Reviewer #2

(Remarks to the Author)
The authors provided satisfactory answers to my and other referees' questions and remarks. I thereby recommend the manuscript for publication.

Prior to publication, I would appreciate if the authors:
(a) Add a paragraph in the main text summarizing their following comment:
"We would also like to point out that the average gate fidelity in the above table is a simple calculation of average gate fidelity. When the cavity starts in the error space, the EsT operation accomplishes the logical gate with as high of a fidelity as it would have if it started in the code space (conditioned on no additional photon loss). Therefore, an error correction operation need not be applied after each gate operation, which is attractive as the error correction operations will have their own infidelities. In this scenario, when an error correction operation is applied after certain number of gates, the fidelity of operation in error space and the number of gates after which error is corrected needs to be considered in calculation of average gate fidelity. We suspect that the improvement in performance due to EsT gate will be even higher than Ord gate in this scenario, but we did not do a quantitative analysis."
In my opinion, this represents an important aspect of the EsT gate schemes compared to previous ones which must be brought to the reader's attention.

(b) Add the figure "Robustness comparison" and the associated explanation to the supplementary text, as I find it valuable for expert audience.

Reviewer #3

(Remarks to the Author)
The authors have revised the manuscript according to my suggestions, and I therefore recommend its publication in Nature Communications.

Reviewer #4

(Remarks to the Author)
I co-reviewed this manuscript with one of the reviewers who provided the listed reports. This is part of the Nature Communications initiative to facilitate training in peer review and to provide appropriate recognition for Early Career Researchers who co-review manuscripts.

Open Access This Peer Review File is licensed under a Creative Commons Attribution 4.0 International License, which permits use, sharing, adaptation, distribution and reproduction in any medium or format, as long as you give appropriate credit to the original author(s) and the source, provide a link to the Creative Commons license, and indicate if changes were made.
In cases where reviewers are anonymous, credit should be given to 'Anonymous Referee' and the source.
The images or other third party material in this Peer Review File are included in the article's Creative Commons license, unless indicated otherwise in a credit line to the material. If material is not included in the article's Creative Commons license and your intended use is not permitted by statutory regulation or exceeds the permitted use, you will need to obtain permission directly from the copyright holder.
To view a copy of this license, visit https://creativecommons.org/licenses/by/4.0/

In the resubmitted manuscript, we have used blue color for all new and modified text.

REPLY TO THE FIRST REFEREE

Referee:
In this paper, the authors demonstrated error semi-transparent (EsT) operations on a bosonic logical qubit to implement universal logical gate set: X, H and T. Previous demonstrations of ET gates were limited to logical phase rotations and implementing universal gate set that are transparent to photon loss errors was an open question. Despite the gates in this work not being completely transparent to photon loss (or being fault-tolerant to ancilla errors), they are still a very important result in the field, showing progress towards realizing fault-tolerant logical gates that are necessary to build quantum computers based on bosonic qubits. The authors have implemented the EsT gates on a binomial kitten code and benchmarked their fidelity using three metrics that are stated in the theory. They observed a five-fold reduction in gate errors after photon jump compared to when using only ordinary gates. The key innovation is a framework based on dynamic encoding subspaces. Rather than requiring the code space and error space to remain fixed during the gate (the usual error-transparent approach), they allow these subspaces to evolve dynamically. This makes it possible to realize near-error-transparent gates using only standard linear control drives. I would recommend publishing this work in Nature Communications as it is an important experimental paper in the field that will be read by many people who work with bosonic qubits for quantum computing and move beyond using them only as a logical memory.

Reply: We thank the Referee their thorough reading and positive remarks on the manuscripts. Below we answer the referee's questions and update the manuscript as necessary.

Referee:
1. The demonstrated EsT gates are implemented in the absence of continuous correction. Can the authors comment on whether the dynamic encoding framework can be combined with autonomous error-correction protocols operating simultaneously with the gate?

Reply: Autonomous quantum error correction (AQEC) protocols described in ref. [1] use passive always on drives and dissipation to continuously correct for errors. Such a scheme could in principle be combined in the optimization with EsT gates to continuously correct for errors. The complex drive amplitudes for the AQEC operation will also need to be optimized to perform error correction from the dynamical error space to the dynamical code space. While theoretically feasible, experimentally modulating the drive of parametric processes to obtain high fidelity target operations remains an outstanding research area.

Referee:
2. How does increasing the code size affect the optimization? Can I extend the gates to other codes like cat and GKP easily?

Reply: The dynamic codespace framework and optimization scheme used in this work is general and hence applicable to other codes like cat and GKP code and larger size binomial codes. For larger codes that can correct for other errors (such as dephasing), a new cost function needs to be added to the optimization for each error. However, for infinite support codes like cat codes and GKP codes, it may be difficult to obtain a high ET and fidelity simultaneously in the current optimization scheme. Additionally, for cat codes with small average photon number, the code and error space has partial overlap, which increases the QEC violation and may set the ceiling for achievable error transparency. So, while it is easy to extend our framework, the ultimately achievable performance is not yet clear.

Referee:
3. Fig.4a shows that the idle evolution with AQEC exhibits lower fidelity than the idle evolution without AQEC. Can the authors provide a quantitative breakdown of the dominant error mechanisms introduced by the AQEC protocol and explain why these outweigh the benefits of photon-loss correction in the idle experiment?

Reply: We thank the referee for raising this important point. First, we would like to point out that the experiment

we have done in Fig. 4a is slightly different than how bosonic memory experiments [2–6] plot the idling curves with and without AQEC operation. In a bosonic memory experiment, an AQEC operation is applied repeatedly after fixed interval of time whereas in our experiment we have applied AQEC only once after waiting for certain amount of time to be able to compare the performance with gates. The AQEC operation introduces errors due to decoherence, readout and qubit pulse errors and control errors. At shorter times the probability of photon loss error is small and hence applying an additional AQEC pulse reduces the net fidelity. At longer times, the probability of photon loss error is larger and hence the AQEC pulse, despite its own errors, improves the net fidelity. We would like to point to Fig. A10b, that shows experimental data that application of AQEC after 34 µs of idling, performs as well as the idling without AQEC and we can expect waiting for longer duration before applying AQEC would improve the idling with AQEC fidelity further.

Compared to idling, the AQEC protocol introduces additional error in few different ways:

1. During the 1 µs pulse, the average photon number in the oscillator ($\bar{n} = 2.2$) is higher than idling ($\bar{n} = 2$) leading to faster oscillator decay and decoherence.
2. During the AQEC operation, oscillator states are entangled with the qubit and hence susceptible to qubit errors, which is not the case for idling.
3. If a qubit error event happens after the AQEC pulse but during the active reset process (readout + conditional qubit reset), it can introduce an error in the (decoded) oscillator state.
4. Readout error wrongly maps the ancilla state which propagates into the decoding pulse. The ancilla reset π pulse has finite bandwidth. So, depending on the cavity state, the ancilla may end up having a small population in excited state, and the cavity will accumulate ancilla state-dependent phase.
5. If the qubit is not fully disentangled at the end of the operation, the readout projection can cause the cavity states to dephase.
6. We estimate about 2% control error in the AQEC pulse, which introduces unwanted dynamics and leakage.

These are the major sources of extra error in the AQEC process that outweigh the benefit of photon-loss correction. Based on simulation of the Lindbladian with all experimentally measured device parameters and losses, we find that about 2% error comes from the active reset process (readout, qubit pi pulse errors), and 5% from decoherence (as opposed to 2.7% for idling). These factors lead to the additional loss in fidelity relative to idling. We have added this discussion to Appendix D.

Referee:
4. The recovery of process fidelities to 0.68 and 0.63 after AQEC. Could the authors provide a clear error budget table identifying the dominant sources of the remaining infidelity? This would help understand whether the current limitation arises from gate errors, decoherence, leakage, or the AQEC protocol itself.

Reply: As explained in the main text, the simulated fidelities for the whole sequence along with AQEC pulse is 0.86 and 0.82 respectively for EsT and Ord gates. This simulation does not include the encode-decode fidelity which is the state preparation and measurement fidelity. In our experiment, encode-decode infidelity is 9%. An error budget for this process is described in third referee's question 9 and newly added text in Appendix D. Beyond this, the AQEC process introduces additional 2% error from readout and active reset process and an estimated 2% control error. From master equation simulations, we estimate that leakage contribution is about $\sim 3\%$. The remaining 2-3% error comes from the coherent errors within the logical subspace from this 8 gate sequence. Control errors and leakage are a major limitation in our present experiments. This is likely due to fluctuation of Hamiltonian parameters and coherence. In future works, optimization can take into account robustness of the pulse to parameter fluctuations and improve this.

Referee:
5. Could the authors clarify how ancilla T1 and T2 specifically contribute to the EsT gate infidelity? In particular, it is not obvious how ancilla dephasing affects the gate operation.

Reply: We performed simulation of gate fidelities for the X gate in absence of ancilla T_1 errors, ancilla T_2 errors and absence of both errors with the help of a master equation solver. The results are summarized in the table below. From these results, we conclude that ancilla T_1 contributes slightly more to the gate infidelity than ancilla T_2, but

their contributions are of the same order of magnitude for our device coherence times. We have included this table and discussion in Appendix D.

Gate	Error	Net fidelity	Code space fidelity	Error space fidelity	ET fidelity	Ideal AQEC
**EsT**	No ancilla T_1	0.980	0.994	0.995	0.760	0.989
	No ancilla T_2	0.979	0.993	0.994	0.740	0.988
	No ancilla T_1 & T_2	0.985	0.996	0.997	0.843	0.994
**Ord**	No ancilla T_1	0.978	0.989	0.345	0.463	0.982
	No ancilla T_2	0.980	0.990	0.346	0.475	0.983
	No ancilla T_1 & T_2	0.984	0.992	0.350	0.533	0.986

TABLE I. Error budget for ancilla T_1 and T_2 errors for EsT and Ord X gate.

Referee:
6. In the Outlook section, the authors suggest using the ancilla g–f transition instead of the g–e transition, which could convert ancilla relaxation errors into detectable erasure errors. This is an interesting idea. Could the authors provide a rough quantitative estimate of the expected improvement in gate fidelity from this approach? In particular,how much of the current infidelity is attributed to ancilla relaxation, and what level of performance enhancement could realistically be achieved by using the g–f transition?

Reply: If the ancilla $|g\rangle$-$|f\rangle$ transition is used instead of the $|g\rangle$-$|e\rangle$ transition, the hypothesis would be that a single T_1 error in the ancilla will lead to the ancilla being in the $|e\rangle$ state at the end of the operation. Hence, (assuming ideal operations) all first order qubit relaxation errors can be eliminated via post-selection. The improved fidelity can be expected to be similar to the row of 'No ancilla T_1' fidelities reported in Table I above. Such a drive scheme and gate fidelity has been analyzed in a recent work by Cai *et al.* [7], which is Ref. [51] in the manuscript.

Referee:
7. Could the authors discuss the contributions of thermal population and readout errors to the reported infidelities of the X, H, and T gates?

Reply: In the reported numbers, thermal population and readout errors contribute 1.6% and 1.2% to infidelity, respectively. These infidelities together decrease the encode-decode fidelity and count as state preparation and measurement (SPAM) errors. SPAM errors remain constant irrespective of the number of gates applied. So, with repetitive application of the gate, gate error free from SPAM errors can be learned. Infidelities reported in the manuscript for X, H and T gate are these SPAM free errors. However, the plots with process fidelities include this SPAM error in measured process fidelity.

Referee:
8. The authors suggest that the observed two-frequency behavior of the qubit g-e transition frequency is caused by the presence of some TLS. Could the authors comment on whether the qubit lifetime is also affected or limited by coupling to these TLS?

Reply: As reported in Table I of the supplementary section, the qubit has a Ramsey $T_{2q,R}$ of 30 µs (with a beating pattern in oscillations in Ramsey experiments) and a Hahn-echo $T_{2q,E}$ of 84 µs. This suggests the presence of TLS(s), and hence the fluctuating qubit frequency is decreasing the T_2 time of the qubit. However, it is not obvious that the qubit T_{1q} is limited by the presence of the same TLS.

REPLY TO THE SECOND REFEREE

Referee:
In their manuscript, Roy, Wetherbee, and Fatemi address an important problem in fault-tolerant computation based on bosonic codes: the realization of fault-tolerant single-qubit gate set. The authors approach the problem for a binomial code (kitten code) for which they extend the concept of error transparent (ET) gates to a variety of single-qubit gates for which the established theory does not apply. Specifically, ET gates are found using numerical optimization constrained on the errors to commute at all times with the time-dependent unitary realizing the gate. The authors' *error semi-transparency* (EsT) extends/changes the constraints to minimize (1) QEC violation (i.e. Knill-Laflamme conditions), (2) leakage outside of code's error subspaces, (3) difference in the coherent evolution between the logical and the error subspaces. This is the main contribution of the work. The authors then proceed to experimentally demonstrate the performance of this method, showing an improvement in the gate infidelity by a factor of 3-6 compared to the ET scheme in case of a photon loss event and that an improvement in the process fidelity when these gates are followed by one round of error correction.

I find that the manuscript makes valuable theoretical contributions to the study of fault tolerance of bosonic codes. Before recommending for the manuscript's publication in Nature Communication, I would appreciate the authors' answers to the following questions and, if relevant, modifications in the manuscript.

Reply: We thank the referee for the overall positive review and their helpful remarks. Below we answer to their comments point by point and modify the manuscript as necessary.

Referee: (a) The authors developed the EsT scheme for gates that fall outside the standard error-transparency formalism which only encompasses phase gates. Though, the T gate is a phase gate and must then be realizable using the ET scheme. I suspect that this explains its better performance compared to the H and X gates which must mix amplitudes. If the authors agree with this statements, I would suggest emphasizing this aspect which shows that EsT scheme is superior to the ET one even for phase gates.

Reply: The referee is right on their understanding. The T gate does not mix the amplitudes of the logical states and hence it can satisfy the Knill-Laflamme condition at all times (or in other words QEC violation can be zero at all times, see Fig. 1 below and updated Fig. A3 in the manuscript supplementary information.). The T gate is also 0.6 µs long whereas the X and H gates are 1 µs long. Due to these two reasons, T gate performance is better than X and H gate.

Previous ET [8] and path-independent [9] schemes required adiabatic gate operations. This required the gates to be slower than our implementation and hence the fidelities in those implementations were lower as they are more susceptible to loss. These gates were also not strictly adiabatic in their implementation and hence could not maintain complete error transparency. Our EsT scheme does not require maintaining adiabaticity due to the dynamical logical space framework and the complete dynamics is captured in the numerical simulation and optimization. This allows us to perform the gate much faster, specifically accomplishing non-Clifford T gate in 600 ns. In comparison, the ET Z gate was 120 µs long in Ma *et al* with a fidelity of 0.78 in code space and 0.68 in error space if photon loss happens during the operation. The path-independent gate was greater than 2 µs long with net fidelity of 0.976. Our T gate has measured fidelities of 0.988 in the code space and 0.85 in the error space if photon loss happens during the operation and a net fidelity of 0.983 (average of code and error space fidelities weighted by the probability of being in the respective subspace). It is worth noting that these previous works had different device coherence, which can be summarized as the other works having better cavity coherence and similar or slightly worse qubit coherence compared to the device in this work. These previous works implemented ET $R_z(\theta)$ (where, θ is dictated by gate duration) and path independent $R_Z(\pi/2)$ operation. It is not the same as our T gate, but we can expect different phase gates to have qualitatively similar performance for a specific implementation.

Therefore, we think a quantitative comparison of different schemes is unlikely to be rigorous enough to include directly in the manuscript. But we agree with the referee that the benefits of EsT scheme compared to ET scheme for phase gates need to be highlighted. So we have added this sentence in main text "We also note that, compared to previous ET and path-independent phase gate implementations [8, 9], these EsT gates can be much faster and higher fidelity as there is no requirement of adiabaticity." We have also added a short discussion regarding this in Appendix D.

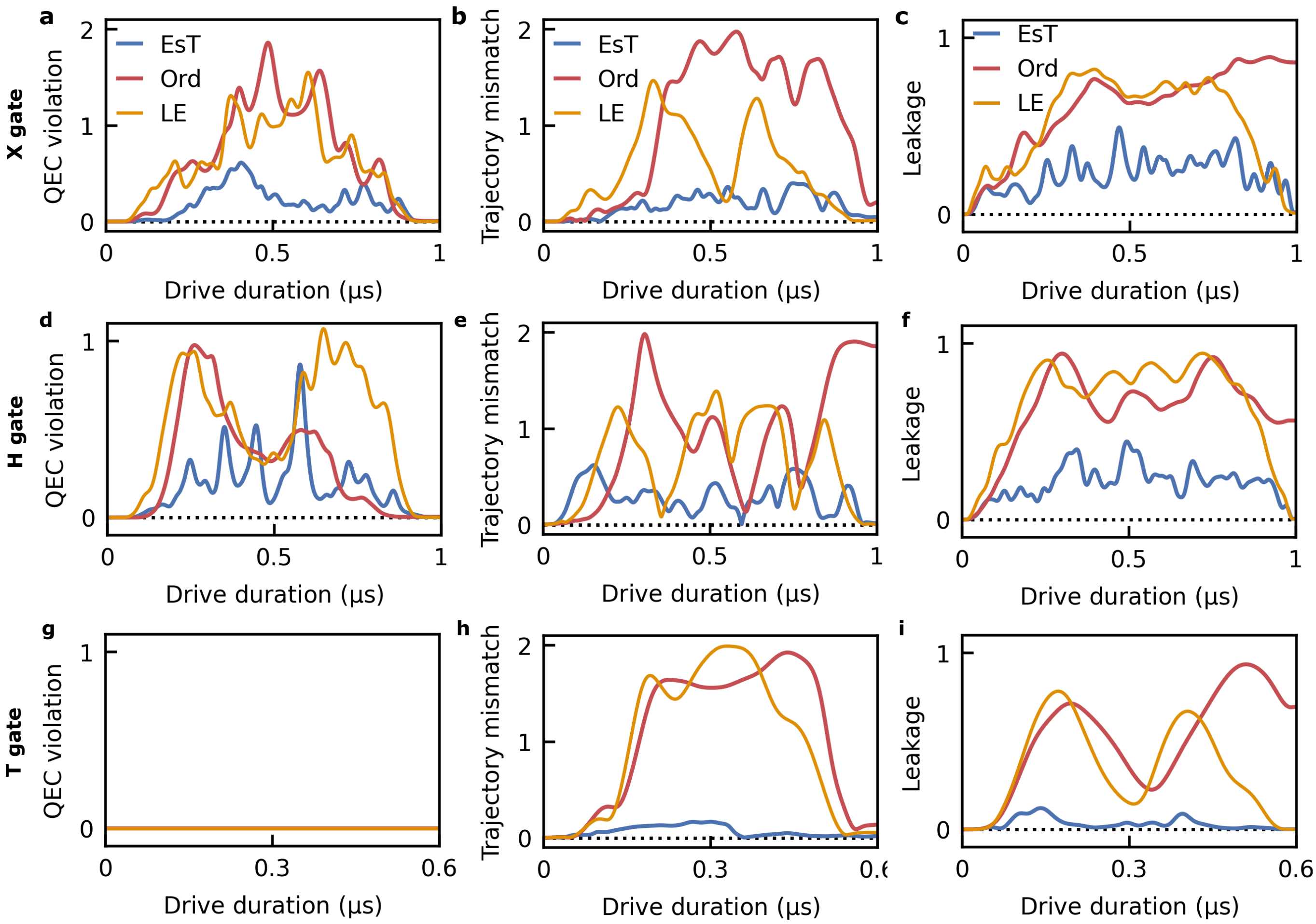

FIG. 1. **EsT, LE and Ord gate comparison:** QEC violation, trajectory mismatch and leakage are calculated from loss less simulations of EsT, LE and Ord gates for X gate (a, b, c), H gate (d, e, f) and T gate (g, h, i). The trajectory mismatch and leakage metrics are calculated for an initial state $|0_L\rangle$ for X gate and initial state $|+Y_L\rangle$ for H and T gates.

Referee:
Along the same line, I would appreciate to see the "EsT, LE and Ord gate comparison" (i.e. Fig. A3) for both H and T gates. Moreover for the latter, it would be valuable to have a direct comparison between ET and EsT schemes where the former would be realized using previously demonstrated approaches [7,8]. It would also be interesting to see the performance of ET and EsT schemes for the Z gate which is another instance of phase gates.

Reply: As per referee's suggestion, we have added the comparison of EsT, LE and Ord gate violation metrics for X, H and T gate in Appendix Fig. A3 of the manuscript, reproduced in this report as Fig. 1. We have included the metrics for an initial state of $|0_L\rangle$ for the X gate and $|+Y_L\rangle$ for H and T gates. As pointed out before the T gate maintains a zero QEC violation, and shows best EsT gate performance.

Ref. [8] (Ref. [7] in original manuscript) uses the PASS gate to accomplish the ET phase gate. In the ideal adiabatic limit the ET gate described in this paper will be 0 at all times for all three metrics. But for a finite 120 µs long gate the adiabatic condition is not strictly satisfied. The transmon often gets excited at the 1-2% level depending on the drive strength, and additional drive induced decoherence of the Fock states is expected. So the three metrics can be estimated to have some violation for the ET gate implementation.

Ref. [9] (Ref. [8] in original manuscript) performs a path independent gate which is fault-tolerant but different than error transparent operation. Also it protects the gate operation from qubit errors and not oscillator errors. So a comparison of these metrics for this kind of gate will not be informative.

These previous works also implemented different gates - ET $R_z(\theta)$ (where, θ is dictated by gate duration) and path independent $R_Z(\pi/2)$ operation. While a comparison of same phase gate such as Z will be a great way to compare the

performance of different schemes, it is difficult to compare schemes that vary widely in terms of their protection against losses and duration. We can expect that different phase gates will have similar fidelity for a specific implementation and compare the operation speed and fidelities qualitatively. We have tried to make such a comparison in our answer to question (a) above.

Referee:
(b) On page 6 (line 316), the authors mention that they model the fidelity as a function of the number of gates. Even though it is explained in the SM, I encourage the authors to add a short explanation of how the model is derived in the main text. Something like "The model assumes that a single photon loss event is equally likely to occur during any gate, and heuristically accounts for the encode-decode fidelity of 60%" would be sufficient.

Reply: We thank the referee for this suggestion. We have added the following sentence in the main text of the review "The model assumes that a single photon loss event is equally likely to occur during any gate, ignores the small probability of two photon loss errors and accounts for state preparation and measurement errors by using a 91% experimentally observed encode-decode fidelity (see App. F for details)."

Referee:
(c) Given that the EsT scheme includes additional constraints compared to the Ord and ET schemes, is the optimization task always convergent, or does it get stuck in local minimas? How robust are the obtained pulses to parameter fluctuations?

Reply: The referee is right to point out that additional constraints may add complications for numerical optimization. While different seeds have different pulses, average photon number, etc., the effect of different initial seeds for convergence is similar for EsT and Ord gate. However for EsT gate, the weights for ET metric and fidelity required careful tuning for convergence with high gate fidelity while simultaneously maximizing ET fidelity. Details of this weight tuning and optimization to simultaneously achieve high gate fidelity and ET fidelity is described in Appendix C, in the paragraph below Eqn. C8. In future works, finding the optimal weights can be made part of the optimization to improve this aspect.

We simulate the H gate operation with our device parameters and losses with a master equation solver and plot fidelity of the gate operations in code space and net fidelity after application of an ideal QEC operation with varying parameters in Fig. 2. We observe that EsT gate is more sensitive to dispersive shift (χ) fluctuation but less sensitive to qubit frequency (ω_q) fluctuation compared to Ord gate. This is only specific to the pulse used in our experiment - other pulses starting from different seeds may have different robustness. In the future, robustness can also be included in the optimization as an additional cost function [10, 11].

Referee:
(d) How do the code states look like throughout the optimized pulse sequence? How does their structure evolve? Do they become more vulnerable to other errors during this time? In other words, would the logical information be lost in case of some (probably unlikely) error events during the pulse sequence and what would these errors be (e.g., transmon dephasing, two or three photon losses)?

Reply: We thank the referee for this interesting question. While evolving, the code states have slightly higher average photon number. This leads to a higher probability of photon loss. With increased photon loss probability, the probability of two or three photon losses also increases. Those are uncorrectable errors even for the idling kitten code and hence we have not considered those in our work. This higher probability is small ($< 1\%$), and it is similar for EsT and Ord gates. As shown in Fig. 1 in this report and Fig. A3 in the Appendix, the QEC condition is also violated to some extent during the operation, leading to loss of some information in case of photon loss in oscillator. This violation is higher for Ord gates compared to EsT gate. We also calculate the Von Neumann entanglement entropy of the oscillator-qubit system. We find that EsT and Ord gates have an average Von-Neumann entropy of 0.44 and 0.47 respectively, indicating they are similarly susceptible to qubit errors. So during the operation the gates are more vulnerable to qubit errors compared to idling but there is no qualitative difference in the nature of this error between the two gates.

In Fig. 3, we also show the code space evolution by plotting Wigner function at different points during the operation for EsT and Ord X gate, conditioned on qubit ground state, for an initial state $|0_L\rangle$. While this shows difference

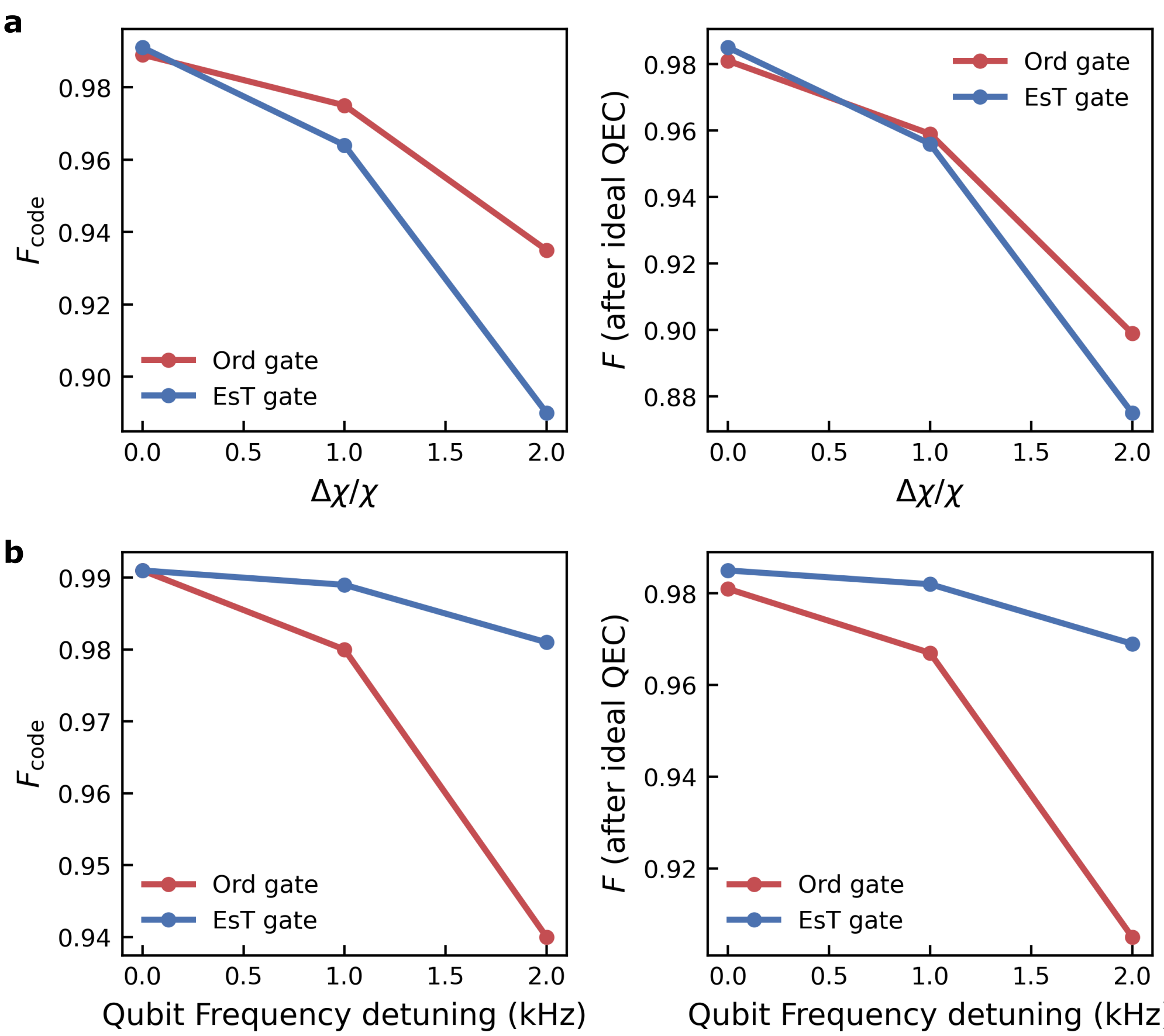

FIG. 2. **Robustness comparison:** We compare the robustness of EsT and Ord H gate by plotting fidelity with variation of (a) the dispersive coupling χ and (b) Qubit frequency detuning. Left column shows the fidelity of the operation in code space and the right side shows the fidelity of the operation after application of ideal QEC. EsT gate is more sensitive to χ variation but less sensitive to qubit detuning variation.

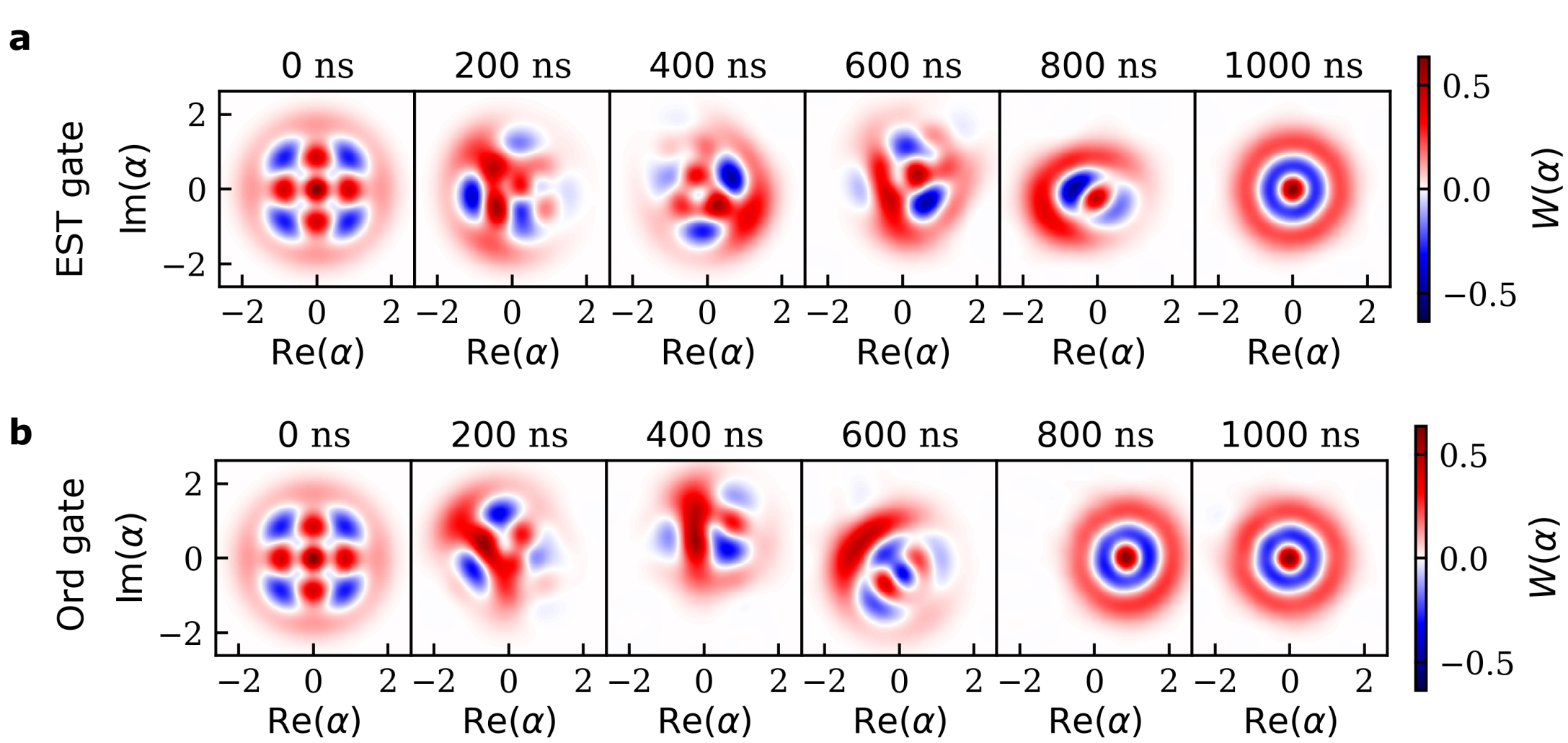

FIG. 3. **Code states during gate operation:** We plot the Wigner function during a X gate operation on an initial state $|0_L\rangle$ for (a) EsT and (b) Ord gate. The Wigner function of the cavity state is calculated conditioned on the qubit being in ground state.

in states during EsT and Ord evolution (as expected), there is no obvious qualitative difference between the two evolutions.

Referee:
(e) The authors showed the gate and process fidelities assuming the system is either in the logical space or in the single-photon error subspace. Although these are relevant metrics for the main message of the work, what will ultimately matter is the averaged performance of the gates. It would therefore be interesting to determine the average gate fidelity for both Ord and EsT schemes and compare their scaling with respect to error rates (and eventually compare them against the ET results from Ref. [8]). How does the performance of the gates vary for different photon-loss and dephasing rates in these cases? Adding such a numerical comparison in the Supplemental Material would be helpful.

Reply: We agree with the referee that an average performance of the gate is the metric that ultimately matters for implementation in any use-case. The main benefit of the EsT gate compared to Ord gate and prior demonstrations is that it keeps the fidelity of the operation in error space intact. We can define an average fidelity by averaging code and error space fidelities (for error space we consider the fidelity in error space when error has happened during the gate and the state ends up in error space and not the fidelity of operation in error space) weighted by the probabilities of being in those spaces. Below we tabulate these fidelity numbers for a few different cavity qubit coherence times for H gate and T gate.

**Qubit Coherence**		**Oscillator Coherence**		**Avg. Gate Fidelity**			
				**H Gate**		**T Gate**	
$\mathbf{T_1}$ (μs)	$\mathbf{T_2}$ (μs)	$\mathbf{T_1}$ (μs)	$\mathbf{T_2}$ (μs)	**EsT**	**Ord**	**EsT**	**Ord**
50	50	200	200	0.983	0.979	0.991	0.988
100	100	200	200	0.989	0.983	0.994	0.99
50	50	500	500	0.987	0.985	0.993	0.991
100	100	500	500	0.992	0.989	0.996	0.994
250	250	500	500	0.995	0.992	0.997	0.995
50	50	1000	1000	0.988	0.988	0.994	0.992
100	100	1000	1000	0.992	0.991	0.996	0.995
250	250	1000	1000	0.996	0.995	0.998	0.997
500	500	1000	1000	0.997	0.996	0.9985	0.997
500	500	2000	2000	0.998	0.997	0.9989	0.998
1000	1000	2000	2000	0.999	0.997	0.9991	0.998

TABLE II. Average gate fidelities for different error rates in qubit and oscillator showing the expected performance improvement with EsT gate in comparison to Ord gate.

We observe that EsT gate always outperforms the Ord gate fidelity in operation. For very high cavity coherence compared to qubit coherence the improvement for EsT gate gets smaller because of two reasons: 1. Qubit errors dominate here, and they start to take some of the logical state to the error space, 2. Coherent control errors also bring states to error space, as we chose to optimize our pulses only up to 0.999 fidelity due to the finite device coherence times. To alleviate these issues, in the future, one could optimizing to higher fidelities, and use the g-f levels of the qubit to post-select on the qubit not experiencing relaxation. The latter strategy is what was implemented in Ref. [7].

We would also like to point out that the average gate fidelity in the above table is a simple calculation of average gate fidelity. When the cavity *starts* in the error space, the EsT operation accomplishes the logical gate with as high of a fidelity as it would have if it started in the code space (conditioned on no additional photon loss). Therefore, an error correction operation need not be applied after each gate operation, which is attractive as the error correction operations will have their own infidelities. In this scenario, when an error correction operation is applied after certain number of gates, the fidelity of operation in error space and the number of gates after which error is corrected needs to be considered in calculation of average gate fidelity. We suspect that the improvement in performance due to EsT gate will be even higher than Ord gate in this scenario, but we did not do a quantitative analysis.

Referee:
(f) Figure 4 benchmark the gates when these are repeated N times and followed by one round of error correction. It would be more insightful to see the performance of repeated application of gate + AQEC. Although Fig. A6(d) presents a simulation of such a benchmark, what did limit you from performing it in experiment?

Along the same line, I suspect the pulse sequence to be imperfect such that some population will get stuck in higher excited Fock states. Could you measure (or estimate) the amount of population outside of the $\{|0_L\rangle, |1_L\rangle\}$ and $\{|0_E\rangle, |1_E\rangle\}$ subspaces? Would one require an operation, similar to the leakage reduction units, that brings the leaked population back into the code space?

Reply: Each of our gates has 1-2% error from oscillator and qubit losses whereas an AQEC process has about 5% error from these losses. This extra error for AQEC pulse is primarily due to its longer duration (including the active reset process). The AQEC has additional 2% error from the active reset process and a 2% control error owing to its complex dynamics. In comparison, each gate has about 1% control error.

The decay curve in an experiment like this, in which AQEC is applied after each gate operation, will be dominated by AQEC (and control) errors rather than individual gate fidelities. Because the AQEC pulse was not our focus and was developed elsewhere (i.e., Ref. [8]), it will be difficult to extract performance improvement between the EsT and Ord gates in this kind of experiment. Therefore, we have not performed this experiment.

With the large error in AQEC pulse, it will also be counterproductive to apply an AQEC operation after every gate operation for an useful experiment. This is because (as noted above) when the cavity *starts* in the error space, the EsT operation accomplishes the logical gate with as high of a fidelity as it would have if it started in the code space (conditioned on no additional photon loss). Therefore, an error correction operation need not (or, should not) be applied after each gate operation. This is attractive because the error correction operations will have their own infidelities.

With master equation solvers, we estimated that about 0.7% probability weight ends up outside of the $\{|0_L\rangle, |1_L\rangle\}$ and $\{|0_E\rangle, |1_E\rangle\}$ subspaces after application of an X gate. From the measured Wigner function we can also reconstruct the states and get an estimate of the leakage population, but the Wigner measurement process is noisy and measured only at a few points in phase space. So estimations from this method are not accurate. From Wigner functions measured before and after application of X gates, we estimate a maximum of 1% leakage per gate operation.

One can imagine applying leakage reduction pulse to remove leakage population. However, simply moving that population to the code space will not be useful as those will remain uncorrectable errors for the binomial kitten code. If they could be separately detected and then reset, then they could become erasure errors in a larger error correction circuit in which the bosonic code is a sub-logical unit. Hardware feedback based pulse calibration [6, 12] can be another approach for reducing leakage for gates in bosonic qubits.

Referee:
(g) Regarding broader use cases of the EsT scheme, could (and how would) it be applied to multi-qubit qubit gates?

Reply: We thank the referee for this interesting question about broader applicability. While our current work focuses on single qubit-oscillator platform, the EsT scheme and dynamic subspace framework is mathematically general enough to be applied to multi-qubit gates. We have not explored this in detail, and we believe it goes beyond the scope of the current manuscript. Nonetheless, we speculate here on potential broader use cases. For multi-qubit gates between bosonic logical qubits, the gates can be optimized for the error set $\{\hat{E}_{i,j}\}$ where, $\hat{E}_{i,j}$ is i-th error we want to be transparent to for the j-th bosonic qubit. This will be a direct implementation of the current framework. For dual-rail erasure qubits and biased noise qubits [13–16], an analogous framework can be used to explore the tradeoff between fast operation and noise bias of the qubit, though this may require more significant adjustments to the cost functions. For biased noise architectures, maintaining strong bias while executing fast and high fidelity gate operations remains a significant challenge. Conceptually our approach to traversing dynamic, intermediate subspace can be merged with space-time control for robust qubit operations and dynamical error reshaping shown in in Ref. [17, 18], and thereby be applied to qubit-qubit operations. Finally, applying this EsT formalism to larger scale concatenated codes, like the surface code, introduces substantial new complexity for tracking stabilizers.

REPLY TO THE THIRD REFEREE

Referee: In the manuscript by Saswata Roy et al., the authors propose a method for error semi-transparent (EsT) operations. By incorporating instantaneous error-transparent (ET) conditions into the numerical optimization procedure, they show that approximately fault-tolerant logical operations on a bosonic logical qubit can be implemented using only linear drives on the bosonic mode and the ancillary qubit. The experimental results demonstrate that, after AQEC, logical operations implemented using the EsT method achieve higher fidelities than non-fault-tolerant logical operations implemented by conventional methods followed by AQEC. This clearly supports the feasibility of the proposed approach. Moreover, the method only requires the addition of an extra cost function during the numerical optimization of the drive pulses, without requiring additional hardware overhead or extra external drives to engineer an effective Hamiltonian.

This work is clearly important and innovative. Its significance includes, but is not limited to, the following points. First, to the best of my knowledge, this is the first experimental realization of fault-tolerant amplitude-mixing logical gates on a bosonic logical qubit. In particular, the manuscript demonstrates fault-tolerant logical X and H gates. Previous experiments had realized fault-tolerant logical phase gates, whereas this work extends the experimental capability toward a fault-tolerant universal gate set. Second, the work addresses a long-standing difficulty in realizing fault-tolerant logical operations through numerical optimization. Numerical optimization is widely used across different physical platforms because it can implement target unitaries with shorter pulse durations. In superconducting bosonic systems, numerically optimized target unitaries can be faster than conventional SNAP + displacement constructions and can therefore yield higher fidelities. However, because the system dynamics during numerically optimized pulses are highly complex, it has been difficult to ensure fault-tolerant properties in such operations. This work overcomes this challenge by introducing instantaneous ET conditions and incorporating the corresponding cost function into the numerical optimization, thereby achieving logical operations that are both fast and approximately fault-tolerant. In addition, the experimental results shown in the manuscript likely do not represent the ultimate limit of this method. In the future, the performance could potentially be further improved by incorporating nonlinear operations, such as multi-photon drives. For the reasons above, I support publication of this work in Nature Communications. Below are several questions and comments that I would like the authors to address.

Reply: We thank the Referee for their detailed and positive assessment of our manuscript. We reply to their comments point-by-point below.

Referee:
1. Could the authors provide numerical simulation results showing whether a strict ET gate against single-photon-loss error a can be achieved if multi-photon drives on the cavity are included? Under what conditions would this be possible? Alternatively, can one obtain logical operations with better performance than those demonstrated in the present work? In this question, I am not considering the additional noise that may be introduced by the nonlinear elements required to implement such multi-photon drives.

Reply: We thank the referee for this important question. Theoretically, a strict amplitude-mixing ET gate is possible to achieve with multi-photon drives. The Hamiltonian and conditions required for such operations are described in the Wetherbee *et al* [19]. In Appendix E of Wetherbee *et al*, a specific drive Hamiltonian required for an exact ET gate has been described. This drive Hamiltonian has an adiabaticity requirement that, in real experiments, would require slow drives. Hence this approach to full ET is not practical, although it does show an existence proof. We have added a sentence in the discussion section with a reference to the above work to give clarity to the reader.

In this situation, numerical optimization of a time dependent drive amplitude of the nonlinear drive will be required to get a fast operation with the same cost function described in the present manuscript for maintaining error transparency. Such fast operations with nonlinear drives are also more likely to occupy higher states of the oscillator and increase the probability of a photon loss. So, we anticipate a trade-off between the speed of the operation, probability of photon loss, and the extent to which error transparency can be achieved for numerically optimized nonlinear drives. We will explore this idea in future works.

Referee:
2. In Eq. (1), can the no-jump evolution be incorporated into the definition, so that a more rigorous instantaneous code space is defined?

Reply: Since the code may only correct no-jump evolution to a given order in the error-rate (e.g., first-order for the binomial kitten code), directly incorporating the no-jump evolution into Eq. (1) may result in a deformed instantaneous code space that reduce correctability of the relevant errors. Instead, the most straightforward way to include no-jump effects within the formalism is to expand the ET error set to include the correctable $\hat{n}$-type errors arising from the no-jump evolution, as was done in the static case in Ref. [19]. Since in this work we focused on the smallest binomial code, which only corrects first-order errors, we did not include mention of these expanded error sets, since the no-jump errors only become relevant at second-order (assuming the instantaneous codewords have equal mean photon number, which is already captured by the KL conditions in Eq. (3)). We have added a remark about including correctable no-jump errors in the error set in Appendix A.

Referee:
3. In Appendix A, the authors mention that if the commutator between the Hamiltonian and the noise operator a is correctable, then the error-closure condition [10] is satisfied. The commutator between displacement and a, or equivalently the transformation of a under displacement, gives a linear combination of a and I, which should be correctable. Does this mean that the reason exact ET operations cannot be realized in the current implementation is mainly the presence of the self-Kerr term and higher-order cross-Kerr or dispersive terms? If so, the statement in the Discussion and Outlook that the linear drive and photon loss do not commute may not be the fundamental reason why errors occurring during the logical gate become uncorrectable. Based on this point, could the authors provide simulation results showing whether linear drives alone can achieve good ET behavior against photon-loss errors in the absence of self-Kerr and higher-order cross-Kerr or dispersive terms? Equivalently, what logical gate fidelity can be achieved in simulations if self-Kerr and higher-order dispersive shifts are neglected?

Reply: The referee is correct to point out that linear drives map $\hat{a}$ errors to linear combinations of $\hat{a}$ and $\hat{I}$ errors, which are both correctable. So linear drives satisfy the error closure condition, though they are not formally 'error transparent' in the strict sense of the term. This may complicate the matter of gate optimization and error correction, and so we leave this to future work.

We simulated H EsT gate without self-Kerr, higher order self-Kerr and higher order dispersive shift. We find that the qubit error post-selected ET fidelity goes up to 0.94 (without self-Kerr etc) from 0.89 (with self-Kerr etc) for H gate. With all errors included in simulation and an ideal QEC, the gate fidelity is 0.988 (without self-Kerr etc) compared to 0.985 (with self-Kerr etc). Both these numbers for ET and gate fidelity with ideal QEC are slightly higher than the simulated values with all Hamiltonian terms considered. The remaining error in error transparency is coming from the continuous code space distortion due to no-jump errors, and the QEC violation because of not adding an extra cost function for this.

We agree with the referee that the explanation in the manuscript is not fully accurate. So we have modified the language in the discussion section, renamed the 'limitations of linear drive' subsection in Appendix A to 'implications of linear drive' and added relevant text there.

Referee:
4. According to Appendix A, "error-closure [10]" is a generalization of error transparency. Should the definition of instantaneous leakage in Eq. (7) of the main text be modified to accommodate this more general ET condition? In my understanding, leakage should correspond to the population outside the sum of all correctable subspaces. For example, for photon loss a and displacement, the transformed error (commutator) contains both a and I components. Therefore, in addition to the target error manifold, part of the state can appear in the code space, which is still correctable. As a result, even when the commutator between the error and the Hamiltonian is correctable and satisfies the generalized ET condition, the quantity defined in Eq. (7) may not vanish.

Reply: We agree that if our dynamic error-transparency construction were generalized to dynamic error-closure, the instantaneous leakage metric, Eq. (7), would have to be modified in the way the referee mentions. However, a generalization to dynamic error-closure would also require changing our defining ET conditions in Eq. (4) and Eq. (5), the trajectory mismatch metric in Eq. (8), and the cost functions in Appendix C. We decided to only consider the restricted definition of error-transparency, instead of error-closure, since our loss function targets error-transparency, not error-closure. We imagine future works could generalize our construction and optimization method to the full error-closure conditions, but allowing the extra freedom provided by these relaxed conditions would likely result in a more complicated loss function that may be less straightforward to optimize. We plan to investigate this in a future work, and, given the chosen scope of our paper, we believe that a dynamic error-closure idea is beyond the scope of

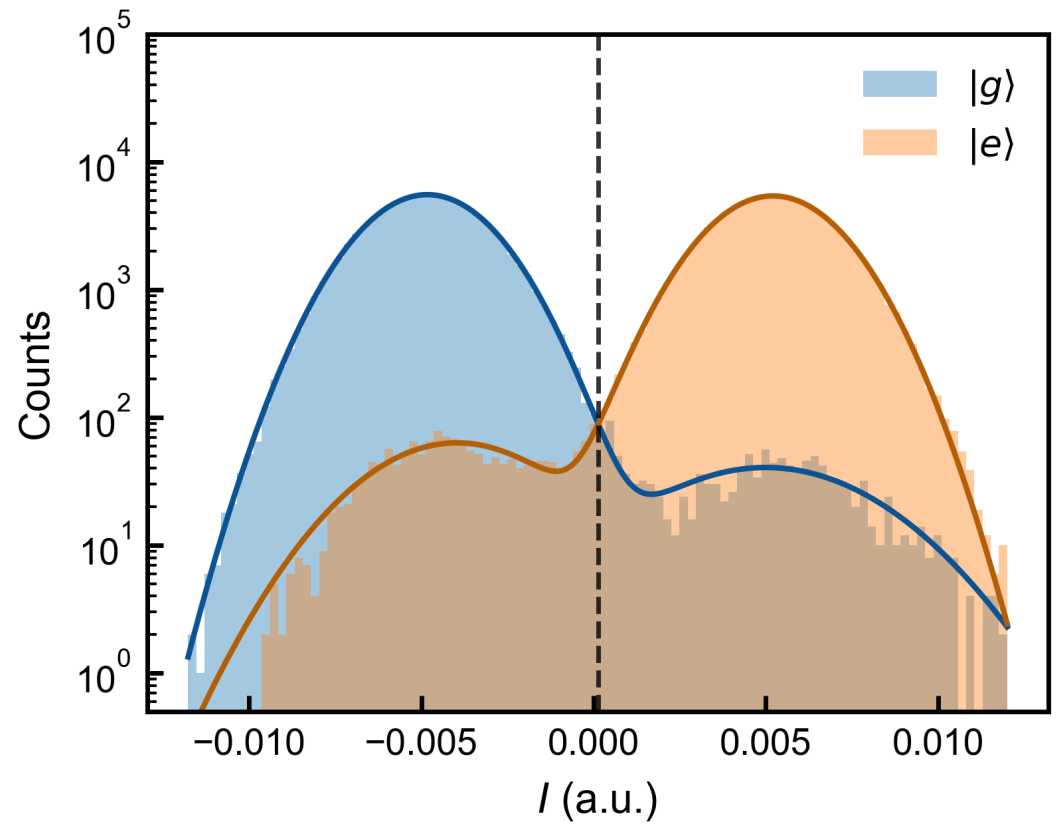

FIG. 4. Readout histogram showing the qubit prepared in ground and excited state and the threshold used to discriminate between ground and excited states for single shot readout.

our current manuscript.

Referee:
5. After the AQEC operation, the entropy in the ancilla is removed through measurement-based conditional reset. However, the manuscript does not explain how this is implemented in hardware, for example on the FPGA. Could the authors provide more information on the feedback latency, the single-shot readout histograms of the ancilla, and how the threshold is chosen to discriminate between the ($|g\rangle$) and ($|e\rangle$) states?

Reply: The ancilla is reset to ground state through measurement-based conditional reset with the conditional π pulse on the qubit. The condition of whether to play the pulse is evaluated directly on the FPGA based on whether the received signal from the readout resonator is greater than the pre-determined threshold for qubit being in excited state. This condition evaluation has a latency of 300 ns. The AQEC pulse takes into account the oscillator Kerr related evolution during this latency delay and the preceding readout of the transmon that takes another 900 ns. In Fig. 4 of this reply document, we show the single-shot readout histogram of the ancilla along with the threshold (black dashed vertical line) that determines whether a specific experimental shot has qubit in ground state or excited state. The readout fidelity for measuring qubit in ground state is 0.993 and for measuring qubit in excited state is 0.983. The average readout fidelity is 0.988. The threshold is determined to maximize the average readout fidelity to discriminate between $|g\rangle$ and $|e\rangle$ states with highest accuracy. We have added this detail in Appendix E of the manuscript.

Referee:
6. In Appendix C, lines 317–320, the authors state: “Further using multi-level ancilla, ancilla ET can be similarly defined and included in the optimization directly. These will be explored in future works.” I am curious how ET for the dominant ancilla noise channels can be realized in this situation. For a single transmon qubit, even when higher levels are included, there is no quantum error-correcting code satisfying the Knill–Laflamme conditions. Moreover, for a strongly nonlinear transmon, damping processes between different levels, such as (—f$\rangle$ to —e$\rangle$) and (—e$\rangle$ to —g$\rangle$), as well as dephasing processes, are generally regarded as independent noise channels. This means that introducing higher levels also requires consideration of additional independent noise processes. Could the authors clarify how ancilla ET would be defined and implemented in this setting?

Reply: In this sentence, we are not referring to a new quantum error-correcting code in the ancilla, but rather are stating that the logical operations on the original binomial code in the oscillator could be made error-transparent to ancilla errors, in addition to photon loss errors. Note that ancilla errors that occur when the ancilla is unentangled from the oscillator are trivially correctable for the binomial code. However, during operations, the ancilla becomes entangled with the oscillator, so ancilla errors occurring during the gate are generically no longer correctable after the operation. So, we have the same notion of error-transparency as for oscillator errors – we want the ancilla errors to pass through the operation so that they remain correctable no matter when they occur during the gate. Such

ancilla ET, AKA "path-independent", operations utilizing multi-level ancilla have already been derived previously in Refs. [9, 19, 20], and we expect that approximately ancilla ET operations could be achieved with our construction by similarly using a multi-level ancilla and including terms in the loss function corresponding to ancilla errors. We have reworded this sentence in the appendix to more generally refer to "fault-tolerance with respect to ancilla errors" rather than "ancilla ET," and have also added references to some of these previous works.

Referee:
7. In Appendix A, lines 21–24, the authors write: "Consequently, a photon loss event during evolution under these terms of the Hamiltonian maps the final logical state $|\psi_L\rangle$ to the corresponding error space state $a|\psi_L\rangle$, up to a deterministic phase." I may be misunderstanding this point, but it seems that this is not simply "up to a deterministic phase," because according to the ET formula around line 85 of the main text, the phase can depend on the jump time (t). Is the reason that this time-dependent phase is a global phase within the error space and therefore does not affect the final result? The authors also mention around line 88 of the main text that it is a global phase. It would be helpful if this point could be clarified.

Reply: We agree with the referee's assessment of this point. The phase acquired depends on the time of photon loss as indicated by the $\phi_j(t)$ in the exact ET condition described in main text. This phase is a global phase within the error space and acts equally on all the logical error space states. Therefore it does not affect the final result. This sentence in Appendix A is now modified to correctly describe the global phase effect: "Consequently, a photon loss event during evolution under these terms of the Hamiltonian maps the final logical state $|\psi_L\rangle$ to the corresponding error space state $\hat{a}|\psi_L\rangle$, with a global phase on the error space states that depends on the time of photon loss.".

Referee:
8. Can the AQEC pulse and the encode pulse, which are also implemented using numerical optimization, be modified to include the EsT condition? Why was EsT not incorporated for these pulses in the present work? If it cannot be incorporated, what are the potential obstacles? If EsT could be included, then in a repetitive AQEC sequence, later AQEC cycles could in principle correct photon-loss errors that occur during earlier AQEC operations.

Reply: With the current optimization strategy used in this work, for an operation to be EsT, an error space must be defined for the corresponding initial and final states. For the encode operation, we can not clearly define an orthogonal error space at the start of the operation. So obtaining a good EsT operation will be difficult as the QEC violation will be high. One could change the optimization protocol to account for different trajectories for photon loss at different time-points to end up in a deterministic error space state to make the encode pulse protected against errors. But this is different than EsT pulse construction and also computationally expensive to optimize. Part of the AQEC pulse that maps codespace back to codespace can be made EsT with respect to photon loss. But when taking error space states to code space state, there is no orthogonal second error subspace for the binomial kitten code considered here. So the AQEC pulse also can not be fully EsT. For larger binomial codes, one could speculate about making the AQEC pulse EsT just for the code space and first error space states.

Our main goal in this work was to show the EsT framework and the improvement in gate performance with EsT optimization. So we did not want complicate the experimental results or error models and include EsT or robustness to other pulses. It is worth noting that all pulses can also be made insensitive to qubit relaxation by using g-f levels of the ancilla instead of g-e levels and post-selection. This can further improve the fidelity of all operations.

Referee:
9. Why is the encode-decode fidelity only 91%? Could the authors provide more discussion of the dominant error sources limiting this fidelity?

Reply: The encode-decode process involves the following steps - qubit state preparation, a 600 ns long encode pulse that maps qubit Bloch sphere to logical Bloch sphere, a 600 ns long decode pulse that maps the logical Bloch sphere to qubit Bloch sphere, and readout of the qubit state. Due to oscillator and qubit relaxation the encode decode pulse suffers about 4.5% incoherent error. The qubit state preparation stage contributes about 2.3% error which includes 0.9% error from qubit thermal population, 0.7% error from oscillator thermal population and 0.7% error in the qubit state preparation pulse. The readout contributes 1.2% error. The remaining 1% error comes from pulse mis-calibration or coherent errors, which can be attributed to fluctuating device parameters and unaccounted for higher-order terms in the system Hamiltonian. This discussion is added in Appendix D.

Referee:
10. Could the authors provide independent experimental characterization data for the parity measurement fidelity? The fidelities for even- and odd-parity measurements are not necessarily identical. For simplicity, in the model analysis in Appendix F, it may be acceptable to use a single value, such as the average of the experimentally characterized even- and odd-parity measurement fidelities, but the independent characterization data should be provided.

Reply: The referee is right to point out that parity measurement fidelity for odd and even states are not necessarily identical. This is because of finite bandwidth of the $R_X(\pi/2)$ pulse used in the parity measurement and that parity measurement maps the odd and even parities to separate excited and ground state of the qubit.

In our experiment, code space and error space fidelity measurements are done as two separate experiments where the second $R_X(\pi/2)$ pulse for the parity measurement is either $-R_X(\pi/2)$ (for even parity state) or $R_X(\pi/2)$ (for odd parity state) such that the parity being measured is mapped to the ground state of the qubit. Then we can use the same decode operation for both cases (and all other experiments) which assumes qubit starting in ground state. We separately prepare code and error space states and measure the fidelity of parity measurement to be 94.4% and 95.2% respectively, so indeed the two parities have unequal measurement fidelity. This error includes the error of the parity measurement, and the readout preceding the parity measurement. Thus the average fidelity of the complete error detection process is 94.8%. We approximate this as 5% error and name it parity mis-assignment error in Appendix F. For simplicity, in our model analysis we use a single averaged value of this parity measurement fidelity.

Referee:
Minor comments: 1. In Eq. (1), the word "span" should be added.
2. In Eq. (7), ρ(t) should carry a subscript, i.e., ρ_j (t), corresponding to the error space (E_j).
3. In Appendix C, the index labeling the six different logical initial states, the index labeling different time steps, and the index labeling different cost functions in Eq. (C8) should be denoted by different letters to avoid confusion.

Reply: We thank the referee for these valuable suggestions. We have updated the manuscript with these corrections.

Referee:
4. In the Discussion and Outlook section, lines 509–517, the authors mention the possibility of using the (—f⟩) state of the ancilla and employing path-independent or error-detectable methods to handle ancilla decoherence. I am curious whether the authors have considered the following issue. Suppose that in the future the method proposed in this work is extended to higher-order bosonic QEC codes, enabling logical gates and correction operations that can tolerate higher-order oscillator errors. After path-independent or error-detectable methods are used to handle first-order ancilla noise, how would second-order ancilla noise be treated, for example a sequence of damping events (—f⟩ to —e⟩) followed by (—e⟩ to —g⟩)? Would an even higher-dimensional ancilla be required?

Reply: We thank the referee for this insightful question. Achieving error correction gain and logical gates that can tolerate multiple oscillator errors with higher order bosonic QEC, handling ancilla errors with path-independence or error detection pose great experimental challenges that need to be overcome in future. So we have not considered a concrete example for how to tackle second order ancilla errors, and prefer not to speculate too far ahead on these points.

SUMMARY OF MAJOR CHANGES

- We have additional experimental data for characterization of readout and parity measurement errors in Appendix E.

- We have added additional analysis of error budget in Appendix D pointing out sources of error for encode-decode operation, AQEC pulse, and benefits of EsT scheme for different loss rates of the device.

---

[1] S. Shirol, S. van Geldern, H. Xi, and C. Wang, Passive quantum error correction of photon loss at breakeven (2025), arXiv:2510.19794.

[2] Z. Ni, S. Li, X. Deng, Y. Cai, L. Zhang, W. Wang, Z.-B. Yang, H. Yu, F. Yan, S. Liu, C.-L. Zou, L. Sun, S.-B. Zheng, Y. Xu, and D. Yu, Beating the break-even point with a discrete-variable-encoded logical qubit, Nature **616**, 56 (2023).

[3] L. Hu, Y. Ma, W. Cai, X. Mu, Y. Xu, W. Wang, Y. Wu, H. Wang, Y. P. Song, C.-L. Zou, S. M. Girvin, L.-M. Duan, and L. Sun, Quantum error correction and universal gate set operation on a binomial bosonic logical qubit, Nature Physics **15**, 503 (2019).

[4] N. Ofek, A. Petrenko, R. Heeres, P. Reinhold, Z. Leghtas, B. Vlastakis, Y. Liu, L. Frunzio, S. M. Girvin, L. Jiang, M. Mirrahimi, M. H. Devoret, and R. J. Schoelkopf, Extending the lifetime of a quantum bit with error correction in superconducting circuits, Nature **536**, 441 (2016).

[5] B. L. Brock, S. Singh, A. Eickbusch, V. V. Sivak, A. Z. Ding, L. Frunzio, S. M. Girvin, and M. H. Devoret, Quantum error correction of qudits beyond break-even, Nature **641**, 612 (2025).

[6] V. V. Sivak, A. Eickbusch, B. Royer, S. Singh, I. Tsioutsios, S. Ganjam, A. Miano, B. L. Brock, A. Z. Ding, L. Frunzio, S. M. Girvin, R. J. Schoelkopf, and M. H. Devoret, Real-time quantum error correction beyond break-even, Nature **616**, 50 (2023).

[7] W. Cai, Z.-J. Chen, M. Li, Q.-X. Jie, X.-B. Zou, G.-C. Guo, L. Sun, and C.-L. Zou, Error-detectable universal control for high-gain bosonic quantum error correction (2026), arXiv:2601.21838.

[8] Y. Ma, Y. Xu, X. Mu, W. Cai, L. Hu, W. Wang, X. Pan, H. Wang, Y. P. Song, C.-L. Zou, and L. Sun, Error-transparent operations on a logical qubit protected by quantum error correction, Nature Physics **16**, 827 (2020).

[9] P. Reinhold, S. Rosenblum, W.-L. Ma, L. Frunzio, L. Jiang, and R. J. Schoelkopf, Error-corrected gates on an encoded qubit, Nature Physics **16**, 822 (2020).

[10] T. Propson, B. E. Jackson, J. Koch, Z. Manchester, and D. I. Schuster, Robust quantum optimal control with trajectory optimization, Phys. Rev. Appl. **17**, 014036 (2022).

[11] P. M. Poggi, G. De Chiara, S. Campbell, and A. Kiely, Universally robust quantum control, Phys. Rev. Lett. **132**, 193801 (2024).

[12] Y. Baum, M. Amico, S. Howell, M. Hush, M. Liuzzi, P. Mundada, T. Merkh, A. R. Carvalho, and M. J. Biercuk, Experimental deep reinforcement learning for error-robust gate-set design on a superconducting quantum computer, PRX Quantum **2**, 040324 (2021).

[13] K. S. Chou, T. Shemma, H. McCarrick, T.-C. Chien, J. D. Teoh, P. Winkel, A. Anderson, J. Chen, J. C. Curtis, S. J. de Graaf, J. W. O. Garmon, B. Gudlewski, W. D. Kalfus, T. Keen, N. Khedkar, C. U. Lei, G. Liu, P. Lu, Y. Lu, A. Maiti, L. Mastalli-Kelly, N. Mehta, S. O. Mundhada, A. Narla, T. Noh, T. Tsunoda, S. H. Xue, J. O. Yuan, L. Frunzio, J. Aumentado, S. Puri, S. M. Girvin, S. H. Moseley, and R. J. Schoelkopf, A superconducting dual-rail cavity qubit with erasure-detected logical measurements, Nature Physics **20**, 1454 (2024).

[14] A. Kubica, A. Haim, Y. Vaknin, H. Levine, F. Brandão, and A. Retzker, Erasure qubits: Overcoming the T_1 limit in superconducting circuits, Phys. Rev. X **13**, 041022 (2023).

[15] H. Levine, A. Haim, J. S. C. Hung, N. Alidoust, M. Kalaee, L. DeLorenzo, E. A. Wollack, P. Arrangoiz-Arriola, A. Khalajhedayati, R. Sanil, H. Moradinejad, Y. Vaknin, A. Kubica, D. Hover, S. Aghaeimeibodi, J. A. Alcid, C. Baek, J. Barnett, K. Bawdekar, P. Bienias, H. A. Carson, C. Chen, L. Chen, H. Chinkezian, E. M. Chisholm, A. Clifford, R. Cosmic, N. Crisosto, A. M. Dalzell, E. Davis, J. M. D'Ewart, S. Diez, N. D'Souza, P. T. Dumitrescu, E. Elkhouly, M. T. Fang, Y. Fang, S. Flammia, M. J. Fling, G. Garcia, M. K. Gharzai, A. V. Gorshkov, M. J. Gray, S. Grimberg, A. L. Grimsmo, C. T. Hann, Y. He, S. Heidel, S. Howell, M. Hunt, J. Iverson, I. Jarrige, L. Jiang, W. M. Jones, R. Karabalin, P. J. Karalekas, A. J. Keller, D. Lasi, M. Lee, V. Ly, G. MacCabe, N. Mahuli, G. Marcaud, M. H. Matheny, S. McArdle, G. McCabe, G. Merton, C. Miles, A. Milsted, A. Mishra, L. Moncelsi, M. Naghiloo, K. Noh, E. Oblepias, G. Ortuno, J. C. Owens, J. Pagdilao, A. Panduro, J.-P. Paquette, R. N. Patel, G. Peairs, D. J. Perello, E. C. Peterson, S. Ponte, H. Putterman, G. Refael, P. Reinhold, R. Resnick, O. A. Reyna, R. Rodriguez, J. Rose, A. H. Rubin, M. Runyan, C. A. Ryan, A. Sahmoud, T. Scaffidi, B. Shah, S. Siavoshi, P. Sivarajah, T. Skogland, C.-J. Su, L. J. Swenson, J. Sylvia, S. M. Teo, A. Tomada, G. Torlai, M. Wistrom, K. Zhang, I. Zuk, A. A. Clerk, F. G. S. L. Brandão, A. Retzker, and O. Painter, Demonstrating a long-coherence dual-rail erasure qubit using tunable transmons, Phys. Rev. X **14**, 011051 (2024).

[16] A. Grimm, N. E. Frattini, S. Puri, S. O. Mundhada, S. Touzard, M. Mirrahimi, S. M. Girvin, S. Shankar, and M. H. Devoret, Stabilization and operation of a kerr-cat qubit, Nature **584**, 205 (2020).

[17] E. Piliouras, D. Lucarelli, and E. Barnes, An automated geometric space curve approach for designing dynamically corrected gates, npj Quantum Information **12**, 46 (2026).
[18] F. Dakis, S. Puri, S. E. Economou, and E. Barnes, Dynamical error reshaping for dual-rail erasure qubits (2025), arXiv:2510.08416 [quant-ph].
[19] O. C. Wetherbee, S. Roy, B. Royer, and V. Fatemi, A Mathematical Structure for Amplitude-Mixing Error-Transparent Gates for Binomial Codes, Quantum **9**, 1890 (2025).
[20] Q. Xu, P. Zeng, D. Xu, and L. Jiang, Fault-tolerant operation of bosonic qubits with discrete-variable ancillae, Phys. Rev. X **14**, 031016 (2024).
